# Characterization of long G4-rich enhancer-associated genomic regions engaging in a novel loop:loop ‘G4 Kissing’ interaction

**DOI:** 10.1093/nar/gkaa357

**Published:** 2020-05-08

**Authors:** Jonathan D Williams, Dominika Houserova, Bradley R Johnson, Brad Dyniewski, Alexandra Berroyer, Hannah French, Addison A Barchie, Dakota D Bilbrey, Jeffrey D Demeis, Kanesha R Ghee, Alexandra G Hughes, Naden W Kreitz, Cameron H McInnis, Susanna C Pudner, Monica N Reeves, Ashlyn N Stahly, Ana Turcu, Brianna C Watters, Grant T Daly, Raymond J Langley, Mark N Gillespie, Aishwarya Prakash, Erik D Larson, Mohan V Kasukurthi, Jingshan Huang, Sue Jinks-Robertson, Glen M Borchert

**Affiliations:** 1 Department of Molecular Genetics and Microbiology, Duke University, Durham, NC 27708, USA; 2 School of Biological Sciences, Illinois State University, Normal, IL 61790, USA; 3 Department of Pharmacology, University of South Alabama, Mobile, AL 36688, USA; 4 Department of Biology, University of South Alabama, Mobile, AL 36688, USA; 5 Department of Biochemistry and Molecular Biology, University of South Alabama, Mitchell Cancer Institute, Mobile, AL 36688, USA; 6 Department of Biomedical Sciences, Western Michigan University Homer Stryker MD School of Medicine, Kalamazoo, MI 49007, USA; 7 School of Computing, University of South Alabama, Mobile, AL 36688, USA

## Abstract

Mammalian antibody switch regions (∼1500 bp) are composed of a series of closely neighboring G4-capable sequences. Whereas numerous structural and genome-wide analyses of roles for minimal G4s in transcriptional regulation have been reported, Long G4-capable regions (LG4s)—like those at antibody switch regions—remain virtually unexplored. Using a novel computational approach we have identified 301 LG4s in the human genome and find LG4s prone to mutation and significantly associated with chromosomal rearrangements in malignancy. Strikingly, 217 LG4s overlap annotated enhancers, and we find the promoters regulated by these enhancers markedly enriched in G4-capable sequences suggesting G4s facilitate promoter-enhancer interactions. Finally, and much to our surprise, we also find single-stranded loops of minimal G4s within individual LG4 loci are frequently highly complementary to one another with 178 LG4 loci averaging >35 internal loop:loop complements of >8 bp. As such, we hypothesized (then experimentally confirmed) that G4 loops within individual LG4 loci directly basepair with one another (similar to characterized stem–loop kissing interactions) forming a hitherto undescribed, higher-order, G4-based secondary structure we term a ‘G4 Kiss or G4K’. In conclusion, LG4s adopt novel, higher-order, composite G4 structures directly contributing to the inherent instability, regulatory capacity, and maintenance of these conspicuous genomic regions.

## INTRODUCTION

Non-coding DNA comprises over 98% of the human genome (1,2) and is predominantly repetitive in nature (3,4). While traditional concepts hold that such repetitive elements generally lack biochemical functionality, current estimates are that over 80% of the genome has some function (5). Some repetitive elements, especially guanine rich (G-rich) sequences derived from transposable elements, are capable of forming transient non B-form secondary structures that have regulatory functions (6).

**Figure 1.**
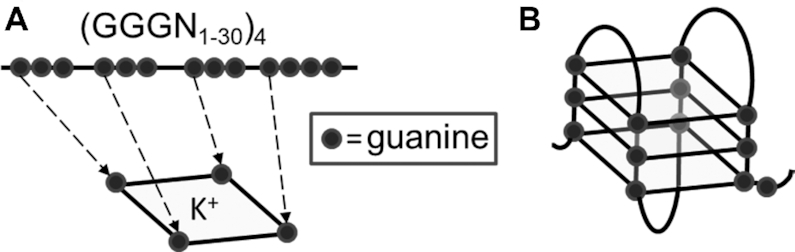
G4 DNA. (**A**) Illustration of guanine quartet with each guanine engaged in four hydrogen bonds and a central potassium cation coordinately bound. (**B**) Structural illustration depicting unimolecular antiparallel G4 DNA.

One prominent secondary structure found in repetitive DNA is G-quadruplex (G4), which can form under physiological conditions. G4 is a four-stranded, highly thermostable, square-planar nucleic acid structure in which guanine repeats are stabilized by Hoogsteen bonds (7–10) (Figure 1). DNA replication and transcription require that stretches of DNA adopt a single-stranded state, which allows for G4 structures to form, and resolution of these structures involves the action of DNA helicases (11). Sequences that support G4 conformations have proven highly variable although the minimum criteria to form intra-molecular G4 DNA have classically been described by the following motif: GGGnGGGnGGGnGGG. Here, G represents guanines that are participating in G4 structure formation, while n denotes DNA spacers of variable length and nucleotide composition (12,13). That said, the definition of G4s has recently been expanded to include structures containing bulges, guanine vacancies, and or mismatches (14). In addition, the spacers separating the guanine repeats can vary in size (*n* = 1–24), and the number of tandem guanines can go well beyond the minimum of three described above (15). Using a stringent loop definition of *n* = 1–7 in search algorithms, over 300,000 putative intra-molecular G4-capable sequences have been identified in the human genome (16,17). More recently, an innovative genome-wide mapping of DNA polymerase stalling under structure-permissive conditions compared to non-permissive conditions identified over 700 000 potential G4 loci (18). Clearly, the sheer number of putative G4 motifs represents an obstacle to studying and fully understanding their impact on the human genome, although insights can be gained from studying individual examples and meta-analyses.

Recent evidence suggests that G4s participate in multiple genomic events. Computational analysis of the human genome indicates that regions capable of G4 formation are not randomly dispersed and are significantly associated with promoters, 5′ untranslated regions, and introns (12,16,19,20). Recently, over 10 000 unique G4 structures were physically isolated from HaCaT cells and ∼1000 G4 structures from NHEK cells using a G4-specific antibody for ChIP-seq analysis (21). Interestingly, G4s from both cell lines were highly enriched in nucleosome-depleted promoter and 5′UTR regions of highly transcribed genes, and the divergence in number detected per cell line suggests that G4 formation and resolution can be tightly controlled based on cell type. Notably, G4 sequences have been reported to play a role during transcription (22), translation (23), recombination (24), replication initiation (25), aptamer binding (26), telomere maintenance (27) and mRNA processing (28). G4 sequences are thought to be tightly controlled during specific cellular processes and may be particularly versatile in regulation due to the variety of different structures they can assume and the potential interplay between structure formation, ligand stabilization, and helicase resolution (12,13).

While accurate maintenance of G4-supportive sequences appears crucial for proper cellular function, these loci are characteristically associated with genomic instability. Both computational analyses and investigations in model systems have documented the susceptibility of G4 sequences to mutagenesis. Computational analysis of human genome small sequence variation databases has shown an increase of small nucleotide variations in sequences that support G4 formation (29). Further, these regions are associated with significant expression variation of downstream genes. Analysis of cancer genomes has also demonstrated that somatic copy-number variations are significantly enriched at regions that stall DNA polymerase under G4-permissive conditions (18). In addition, analysis of cancer translocations has shown that ∼70% of translocation breakpoints are capable of G4 formation (30), with structure formation demonstrated *in vitro* at *c-MYC* t (8;14), *HOX11* t (10;14), *BCL2* (t14;18), and *TCF3* t (1;19) breakpoints (22,30–32). In yeast, the *TCF3*-breakpoint G4 sequence was further shown to lead to loss of a chromosomal arm under conditions that support G4 formation including high transcription and increased negative supercoiling (32). Detection of gross chromosomal rearrangements using other human G4 sequences in yeast was magnified by ligand stabilization of G4, increasing G4 repeat number or removal of G4 helicase Pif1 (33–35).

To date, only a small number of G4 loci have been examined, and the number of potential G4 loci (∼300 000–700 000) constitutes a significant obstacle to identifying biologically relevant loci that are prone to instability and/or regulation. Accordingly, we developed a novel approach and open-source program (LG4ID) that identifies putative G4-forming sequences using search parameters modeled after the size and composition of human immunoglobulin switch region, laboratory-validated G4 sequences (Supplementary Information 1). We further describe the experimental evaluation, functional analysis and disease associations of 301 large-G4 (LG4) sequences thus identified, and document a novel secondary structure assumed by these sequences. We conclude that LG4ID is highly successful in identifying biologically relevant, large guanine-dense G4 sequences in the human genome.

## MATERIALS AND METHODS

### Python 3-based program for identification of LG4s–LG4ID

In order to identify long G4-capable regions (LG4s) present in the human genome, we wrote a Python 3 program to search a FASTA formatted sequence file for long G-triplet regions likely to form G4. The program identifies LG4 motifs based on the density of G-triplets within 1.5 kb sequence windows sliding one base pair per iteration and does not account for loop length. In order to define the minimal density of G-triplets needed to call a LG4, we modeled our search program after the G4-capable sequence density within the human immunoglobulin mu (*Sμ*) switch region (11–13,24,36). Sliding windows were applied, with a minimal output threshold of (GGG) X 121 for every 1.5 kb of sequence. To identify G-rich sequences on the + and – stands of the genome, CCC density was also determined as above then combined with the GGG search output data (for more details see Supplementary Information 1). LG4ID was used to identify all LG4s (with parameters stated above) in the human genome (hg38) after which each LG4’s location and identity were confirmed manually. A Web-hosted LG4ID LG4 search tool and LG4ID source code are available at: http://omnisearch.socsouthalabama.edu:8080/g4search#

### Detailing LG4 genomic locations, putative regulatory abilities, COSMIC and FusionGDB associations and Hi-C interactions

Output of the LG4 identification program was used to map each individual LG4 location on Ensembl Release 69 (hg19) (37) and further confirmed for LG4 genes (genes containing an LG4 sequence in their transcript or within 10kb of their UTR) on Release 77 (hg38) (38). Statistics for genomic location with respect to transcription were analyzed using chi-square. The enrichment for chromosome location used one-way ANOVA followed by individual unpaired two-tailed t-tests. Significant enrichment of LG4 at the distal end of the chromosome was calculated using an unpaired two-tailed *t*-test. Potential regulatory functions and ChIP-Seq pull down data was obtained using Ensembl77 with the Regulatory Build filter turned on (39). Significance for regulatory ability was calculated using chi-square. Full COSMIC (40) and FusionGDB (41) translocation and gene fusion datasets were downloaded and significant LG4 associations calculated using an unpaired two-tailed *t*-test. To enumerate reported interactions between LG4s and other regions of the genome, chromosomal interactions between LG4 loci (or randomly selected, size matched control loci) and other genomic locations were identified in UCSC Genome Browser Hi-C and Micro-C tracks providing chromatin folding data from Micro-C XL and Hi-C experiments examining HFFc6 (foreskin fibroblasts) and H1-hESC (embryonic stem cell) cell lines (42–44).

### Identification of potential loop interactions

Internal LG4 G4 loops were initially identified by extracting all unique sequences (>4 nt and <40 nt) located between neighboring GGGs within an individual LG4. Reverse complements of LG4 G4 loops sequences were then identified by Blast+ (2.2.27) alignment of all unique loop sequences extracted from a single LG4 against all other unique loop sequences located within the same LG4 using –strand minus, -evalue 10 000, and -word_size five parameters (45). Putative internal LG4 loop interactions between distinct G4 loops were required to be at least 6 bp in length with reported 6–9 bp alignments also requiring 100% complementary and >9 bp alignments requiring ≥90% complementarity.

### Database for Annotation, Visualization and Integrated Discovery (DAVID) analysis

All LG4 proteins were analyzed on the Database for Annotation, Visualization and Integrated Discovery (DAVID), a web based program that provides annotation tools for researchers to understand the biological meaning behind large list of genes identified in microarray or bioinformatic studies (46). DAVID can be used to identify protein interactions, common genetic locations, common pathways, disease relevance and multiple other analyses. Genes were entered into the web interface by their Ensembl gene ID and then analyzed using the functional annotation tool to investigate any statistically significant similarities and relationships between our genes.

### Identification of disease genes

Genes that are potentially regulated by a LG4 (associated enhancer, gene promoter, or within the transcribed gene sequence) were evaluated for known human disease association. Each corresponding gene was searched on Malacards database (47) and Wikigenes literature search (48) to identify any potential involvement in disease.

### G4 density calculation

A program called QGRS mapper (49) was used to determine the potential of each sequence to form G4. This was accomplished using the following filters: A max motif length of 45 nucleotides, minimum G group of 3, and a loop size 0–36 nucleotides (selects for intra-molecular G4 only). The output of the analysis was mapped to the location of the LG4, and the number of individual non-overlapping G4 motifs per kb (G4 density) was calculated for each LG4. Additionally, positions directly adjacent of LG4, and control loci were also calculated in this method. Statistics were calculated using one-way ANOVA followed by unpaired two-tailed t-tests.

### Identification of human genome variation densities

The location of all individual SNPs, insertions and deletions were obtained from the dbSNP database (50) and mapped to LG4s found in protein transcript regions as well as surrounding introns (exons excluded from analysis) using Ensembl release 69 (37). The density of small sequence variants was calculated by number of SNPs, insertion, or deletion events per 100 base pairs (bp) for LG4 and regions 1–2000 bp away from LG4s. Statistics were calculated for each type of mutation using unpaired two-tailed *t*-tests.

Copy number variations (CNVs) for LG4s found in protein transcript regions were downloaded from the database of genomic structural variation (dbVAR) on NCBI.org (51). The density of CNV breakpoints was calculated using the exact reference points as the density of LG4 motifs above, as well as the surrounding region in 1 kb increments up to 3 kb, and the rest of the transcript containing the LG4. Statistics were calculated using one-way ANOVA followed by individual unpaired two-tailed *t*-test.

### Circular dichroism

Oligonucleotides for circular dichroism (CD) studies were designed by using representative repeat units found in LG4 sequences and synthesized by Operon (Eurofins MWG operon LLC, Huntsville, AL, USA). CD analysis was performed using an Aviv model 215 CD spectrometer at 37°C. Spectra were taken in 1 cm path quartz cells containing 12 μM G4 or GCA oligonucleotide in 10 mM Tris–HCl, pH 7.6, 1 mM EDTA and 100 mM KCl. The molar ellipticity was measured from 220–300 nm and recorded for 3 scans in 1 nm increments at a 1 s average time.

### Primer extension assays

LG4 containing phagemids for extension assays were obtained by cloning PCR amplified genomic fragments, or cloned from amplification products using overlapping primers in a standard PCR reaction. PCR products were gel purified and TOPO cloned (Invitrogen) into pCR2.1. Fragments were cloned in both orientations and were verified by Sanger sequencing (University of Illinois Core Sequencing Center). Templates for extension assays are shown in Supplementary Table S4 and range from 120–1300 bp. The size variation is due to the inability for some larger LG4 sequences to be cloned. Closed-circular single-stranded DNA was obtained using M13K07 helper phage (NEB) according to the manufacturer's instructions.

Klenow Polymerase extension assays were performed as described in (52) and developed from previous G4 assays (53,54). A ^32^P 5′ end labeled forward primer (standard M13 F) was annealed to the single-stranded phagemid templates. In addition to the manufacture's buffer (NEB), KCl or LiCl was added to a final concentration of 25 mM. After annealing, Klenow extension reactions were performed at 37°C for 8 min, then stopped by the addition of an equal volume of 90% formamide and 1 mM EDTA followed by heating to 90°C for 20 min. Products of polymerase extension were resolved by 8% denaturing PAGE (19:1) with 7 M urea and 0.5× TBE, at 700 V at room temperature. Gels were then dried and images were captured by phosphorimaging using a Molecular Dynamics Storm 840 phosphorimager (Amersham/GE). Each template was assayed at least twice.

### Yeast strain construction

Yeast strains for the *LYS2* reversion assay were derived from W303 (*MATa, leu2–3,112 ura3 his3–11,15 ade2–1, trp-1*) (55). A130 bp *DIP2C* LG4 fragment or GCA repeats was used to replace the CORE fragment (56) in the *LYS2* reversion window to create a +1 or –1 frameshift allele (previously described in (57)). The orientation and integrity of the inserted sequences for all strains was verified by Sanger sequencing (Eton bio) before further analysis. *PIF1*, *RRM3*, *LIG4* and *RAD51* were removed by one-step allele replacement with a PCR-generated cassette containing hygromycin or kanamycin selectable markers. High transcription was driven by the *LYS2* promoter with a galactose-regulated (*GAL1*) promoter linked to a selectable marker (58).

### Mutation rates and spectra

Cultures inoculated from single colonies were grown to saturation (3 days) in YEP-GE (1% yeast extract, 2% Bacto-peptone, 2% glycerol and 2% ethanol). To induce high transcription in strains containing the *GAL1* promoter, 2% galactose was used instead of glycerol and ethanol. Lys^+^ revertants were selected on synthetic complete media lacking lysine (0.17% yeast nitrogen base, 0.5% ammonium sulfate, 2% agar and 0.13% Hartwell's complete amino acid mix lacking lysine). The total number of cells in each culture was determined by plating on non-selective YPD (1% yeast extract, 2% bacto-peptone, 2% dextrose and 2% agar) medium. Mutation rates were calculated using method of the median (59), and 95% intervals confidence intervals determined as previously described (60). The rates of a specific mutation type was calculated using its proportion in the corresponding mutation spectrum; associated confidence intervals were calculated using the right-triangle rule (61). The mutation spectrum was generated by isolating genomic DNA independent mutants, followed by PCR amplification and sequencing of the *LYS2* reversion window. Mutation rates were based on 24 independent cultures and spectra on the analysis of 42 independent revertants.

### *In vitro* G4-DNA formation and native polyacrylamide gel electrophoresis

Commercially synthesized G4-DNA oligonucleotides (Integrated DNA Technologies) were rehydrated using ultra-pure DNA grade H_2_O to a final concentration of 0.25 mM. To form tetramolecular G4-DNA, aliquots of each sample were boiled in a thermalcycler at 98°C for 10 min, and subsequently held at 80°C as previously described (62). Pre-heated KCl solution (folded G4) or an equal volume of pre-heated ultrapure H_2_O (unfolded controls) was added to each aliquot. The final concentration of KCl varied from 50 to 250 mM. The samples were then allowed to slowly cool to room temperature and stored at –4°C until needed (max 3 days). The formation of tetramolecular G4-DNA structures was confirmed via gel electrophoresis using 10 and 20% non-denaturing polyacrylamide gel and Tris/boric acid buffer. To visualize the oligonucleotides and differentiate between G4/ssDNA and dsDNA, resulting gels were stained first with ethidium bromide, imaged on UV Transilluminator FBTIV-88 (Fisher Scientific), then re-stained with Thioflavin T and imaged again.

## RESULTS

### Identification of large G-triplet dense G4 regions in the human genome

**Figure 2.**
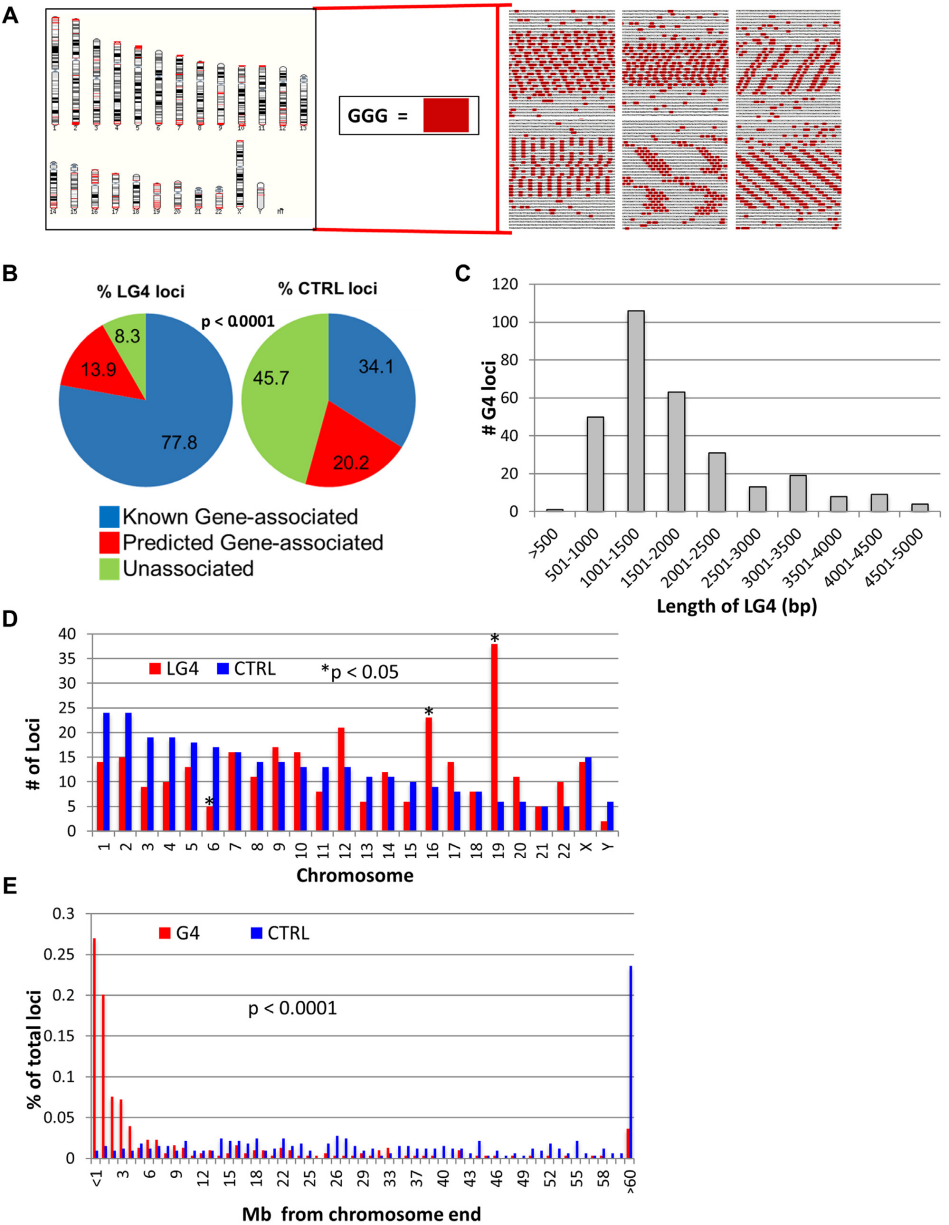
LG4s in the Human Genome. (**A**) Genome-side distribution of LG4 (red bars) on the left and examples of hits on the right with >3 bp G-repeats that are highlighted red. (**B**) Distribution of locations with respect to annotated genes for LG4 and control loci. (**C**) The length distribution of LG4 loci. (**D**) The number of LG4 and control loci on each chromosome, with asterisks indicating a significant difference. (**E**) Distribution of LG4 loci with respect to the distance from the ends of each chromosome.

Most G4-predicting programs utilize an algorithm based on a minimal definition that solely identifies individual G4 motifs (14). In contrast, next generation G4 search tools employ more complex pattern-based rules (recently reviewed in (63)). As an example, G4Hunter considers the relative likelihood of both canonical and non-canonical structures (64). That said, both traditional and next generation strategies predict hundreds of thousands of minimal G4 capable sequences across the human genome (14). Because long guanine-rich minisatellites (33) and guanine-rich immunoglobulin switch regions (13,24) both adopt G4 structures and are associated with DNA breaks, we reasoned that a high density of guanine repeats within an ∼1 kb window could likely have similar impacts on the genome. These criteria would identify sequences that are not overly abundant, thus making an in-depth analysis possible. To identify a panel of loci containing extensive G4 sequence motifs, we searched for large genomic stretches significantly enriched for guanine-triplets (G-triplets) instead of focusing on shorter, more rigidly defined G4 motifs. G-triplets were counted because they are the basic sequence necessary for G4 structure formation. Further search parameters (e.g. window size) were based on the immunoglobulin switch region *Sμ* (Supplementary Information 1), which is a G4-forming recombination site recognized by mismatch repair factors (24,52). The *Sμ* guanine density of 120 G-triplets/1.5 kb window, which is a much lower density of G-triplets compared to other well-known G4s such as telomeres (65), was used to train our analyses. Modeling our LG4ID search program on these parameters, we identified 301 loci containing a density of at least 80 GGG repeats/kb (Figure 2A). The 301 long G4-capable regions (LG4s) we identified in the human genome ranged from 199 to 4973 bp in length (subset shown in Figure 2A). Although the initial search window was 1.5 kb, several smaller length regions contained a high density of G-triplets surrounding the larger repetitive unit and, therefore, met our minimal G-triplet requirements.

LG4 and control loci (301 sequences of identical lengths randomly selected from the human genome) were categorized based on their relative locations to known genes including: overlap with or within 5 kb of a known gene (Known Gene-associated), overlap with or within 5 kb of a GENSCAN-predicted gene (66) (Predicted Gene-associated), or unassociated with gene transcripts (Unassociated). Similar to previous reports (19,67), LG4s were significantly (*P* < 0.00001) enriched within and around known genes with 77.7% of LG4s versus 33.9% of controls occurring in annotated loci (Figure 2B, Supplementary Table S1). The LG4s associated with known genes were primarily located in introns (∼77%) or upstream of the transcription start site (∼12%). One exonic LG4 was identified and located in homeobox gene *TPRX1*, and comprised a majority of the coding region. Intronic LG4s were found to occur in fairly similar percentages on transcribed (56.5% CCC mRNA) and non-transcribed strands (43.5% GGG mRNA), and although beyond the scope of the current work, a more thorough evaluation of these loci will ultimately be necessary to determine if this difference is statistically significant (68). In contrast, LG4s located within 5 kb upstream of an annotated TSS are more than 3.5 times more likely to occur on the transcribed strand (Supplementary Table S2).

All LG4 regions identified by LG4ID were visually confirmed in human genomic sequence, and the endpoints of G-triplet containing repetitive units used to refine locus length. All but three loci identified were over 500 bp in length; the largest was 4973 bp and the average size was 1843 bp (Figure 2C). The chromosomal distribution of LG4 loci was not random with significant enrichment on Chromosomes 16 and 19, and depletion on Chromosome 6 (Figure 2D). In addition, LG4s within and directly flanking gene transcripts, referred to hereafter as ‘LG4 genes’, were evaluated in depth using the Database for Annotation, Visualization and Integrated Discovery (DAVID) interface (46). Similar to the occurrence of LG4 loci, we found Chromosome 16 and 19 both significantly enriched for LG4 genes (Supplementary Table S3.1). Cytogenetic bands are specific genetic regions that can be detected using stains on metaphase chromosome spreads (69,70). DAVID analysis also identified significant enrichments of LG4 genes in 18 cytogenetic bands on 14 chromosomes (Supplementary Table S3.2). Finally, we also found LG4 loci significantly enriched at the ends of chromosomes, with 46% located less than two Megabases (Mb) from telomeres, and 67% within 6 Mb (Figure 2E). To fully ensure the bias towards chromosomal ends is not simply due to the occurrence of expanded telomeric repeats, human telomeric repeat (TTAGGG) sequences (71) were identified in each LG4. While eight LG4s found close to chromosomal ends do average 43.5 TTAGGGs/1000 bp suggesting a potential relationship between (or origin from) these eight LG4s and their neighboring telomeres, over 97% (293/301) of the LG4s described in this work average only 0.6 TTAGGGs/1000 bp. Taken together, the significant enrichment of LG4s with defined genetic features indicates that their distribution in the human genome is non-random and of likely functional relevance.

### LG4 repeats are capable of G4 formation

**Figure 3.**
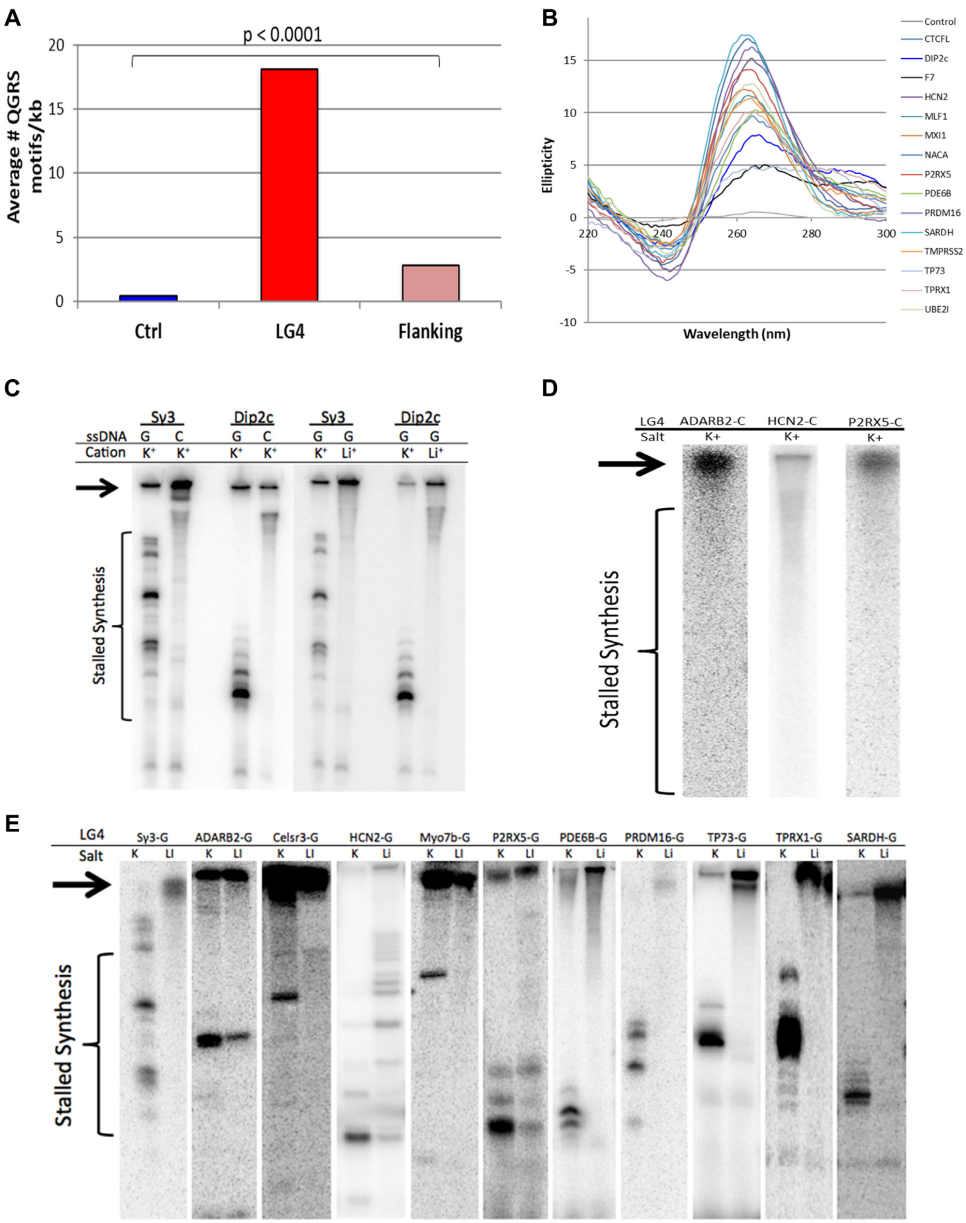
LG4s are capable of G4 formation. (**A**) The average number of non-overlapping G4 motifs predicted by QGRS mapper per kb (G4 motif density) in LG4s, regions directly flanking LG4s (flanking) and control loci (Ctrl). (**B**) Circular dichroism ellipticities of oligonucleotides representing LG4s. (**C**) Klenow DNA polymerase primer-extension reactions of G- or C-rich single stranded *Sγ3* or *DIP2C* DNA templates in buffer containing K^+^ or Li^+^. (**D**) Primer extension reactions of the C-rich LG4 strand of loci shown to stall polymerase in G4 supportive and non-supportive conditions. Reactions were in G4-supportive conditions (K^+^) (**E**) Klenow primer-extension reactions on LG4 G-rich templates in different G4-permissive conditions. LG4 sequences are denoted at the top of lanes; areas of stalled DNA synthesis is denoted by the brackets and full-length replication products are denoted by arrows. *Sγ3* and *Sμ* are G4 model sequences previously shown to form G4 *in vitro* (24,52).

Quadruplex forming G-Rich Sequences (QGRS) mapper is a web-based program for identifying individual G4 motifs in a given DNA sequence (49). We used QGRS mapper to corroborate how successful our program (LG4ID) was at identifying loci containing a dense concentration of G4 motifs. All individual LG4s, 1.5 kb on the 5′ and 3′ sides of the LG4 sequence, and control loci were queried in both orientations with QGRS, and the average number of non-overlapping G4 motifs/kb (G4 motif density) was calculated. On average, LG4s contained 18 individual G4 motifs/kb, a 45-fold increase compared to control loci, and a 6.4-fold increase compared to sequences directly flanking LG4 (*P* < 0.0001) (Figure 3A). Notably, the increase in the density of G4 motifs in the sequences directly flanking LG4 compared to control loci indicates LG4 are found in G-rich areas compared to control loci. To confirm these findings, we elected to employ another G4 prediction algorithm (G4IPDB G4 predictor tool) (72) as a second means of evaluating G4s density. We find the programs largely in agreement with QGRS and G4IPDB respectively predicting an average of 18.3 and 21.7 putative G4s/1000 bp within LG4 loci versus 0.4 and 1.3 in control sequences. Also of note, we find three additional in-silico G4 prediction tools, ImGQFinder (14), Quadparser (73) and AllQuads (74) each independently predict the formation of at least one G4 secondary structure by sequences within each of the LG4s described in this work, and furthermore, that 100% of the LG4s we find embedded within known protein coding loci were previously identified as likely forming G4 in vitro by Chambers *et al.* (75) via a high-resolution sequencing-based G4-sequencing (G4-seq) approach based on the fact that G4s can block polymerases.

While computer algorithms can predict the G4 folding potential of LG4 sequences, the actual ability of these sequences to form structures must be confirmed *in vitro*. Although the LG4s described in this work average over 1800 bp in length, they are composed of shorter minimal G4-capable sequences much like telomeres whose minimal G4 motif G4 structures have been extensively verified (65). As such, we have employed two separate verifications that individual minimal G4 motifs within LG4s do in fact form G4 secondary structures similar to minimal G4 motifs in telomeres. To achieve this, subsets of transcribed LG4s were selected for experimental validation by two distinct methods. Firstly, minimal G4 motifs found in 15 LG4s (chosen due to their diverse G-repeat sequence composition and being located in frequently transcribed regions) (Supplementary Table S4) were assayed using circular dichroism (CD). CD measures the differential absorption of left and right polarized light from chiral molecules in solution in order to identify structural conformations (76). G4 can adopt two different conformations: parallel and anti-parallel, which describes the directionality of the DNA strands composing the structure (15). Parallel G4 DNA results in a CD spectrum (ellipticity) with a peak at ∼260 nm and dip at ∼240 nm. Anti-parallel G4 structures show a peak at ∼295 nm and dip at ∼260 nm (77,78). Notably, all LG4 oligonucleotides tested produced spectra characteristic of parallel G4, although there was also evidence of anti-parallel G4 formation for *F7* LG4 (black line peak 295, Figure 3B). These results are not that surprising and corroborate a study demonstrating that parallel G4s are abundant throughout the human genome (79).

Next, as a second, independent confirmation that LG4 sequences can form G4 DNA *in vitro*, 11 transcribed LG4 sequences were selected for testing by a polymerase extension assay. Sequences representing LG4 repeats ranging from 120 to 1300 bp were cloned and closed-circular single-stranded templates generated (Supplementary Table S5). In polymerase extension assays, polymerase pausing at G4 is monovalent-cation dependent and occurs only when the guanine-rich strand serves as the template (53,80). There is a hierarchy of monovalent cations able to stabilize G4 that is dependent on the temperature, cation concentration, specific sequence, and therefore structure. K^+^ ions strongly promote G4 assembly while other monovalent ions such as Li^+^ are poorer stabilizers (81–85). For the *Sγ3* control sequence, extension by Klenow polymerase was blocked in an orientation and K^+^-dependent manner, which indicates that G4 formation on the template strand blocks DNA synthesis (52). Similarly, a 130 bp segment of the *DIP2C* LG4 sequence, which was used in subsequent yeast genetic assays, also stalled Klenow extension in orientation- and K^+^-dependent manners (Figure 3C). Notably, all LG4 sequences examined exhibited K^+^-associated stalling relative to Li^+^, although to varying degrees; three of these LG4 sequences (*P2RX5*, *HCN2* and *ADARB2*) were also able to stall Klenow in Li^+^, reflecting the capacity of this ion to weakly support G4 folding. In order to rule out stalling due to non-G4 conformations, such as hairpin DNA, the reverse complement C-rich strands of these three LG4s were assayed in K^+^, and we found none were capable of stalling Klenow extension (Figure 3D). As such, we conclude that the LG4 G-rich strands stalled polymerase advancement (Figure 3E), consistent with the formation of G4 structures in the template.

Finally, as an initial examination of the ability of LG4 sequences to form G4s in vivo, we asked if the number of LG4s immunoprecipitated (IP’d) in existing G4 ChIP-Seq datasets (obtained using a G4-specific antibody) (86) were enriched over matched control loci. Notably, we find a statistically significant (*P* < 0.0001) enrichment for LG4 loci sequences (29.1%) over control loci sequences (4.9%) in G4 IPs (Supplementary Table S6).

### LG4s are enriched for regulatory sequences

As previous genome-wide analyses (19) have found G4 sequences highly enriched in promoters, we next asked whether LG4 sequences are associated with known regulatory elements. Because of its comprehensive nature, we initially selected the Ensembl Regulatory Build Database (38,39) (based on publicly available, experimentally derived data sets from DNase1-Seq, FAIRE-Seq and ChIP-Seq studies) to examine potential regulatory roles of all gene-associated LG4s (along with size matched control loci) and found LG4s associated with regulatory elements two-fold more often than control loci (*P* < 0.001) (Supplementary Table S6). We also examined available NCBI SRA datasets (87,88) to determine if LG4 sequences in available transcription factor (TF) ChIP-Seq datasets were significantly enriched over controls. We found a significant (*P* < 0.0001) enrichment for LG4-loci sequences (44/198) over control-loci sequences (6/144) and identified over 80 interactions at 44 LG4 loci involving 26 different TFs (Supplementary Table S6). The most prominent LG4-interacting TF identified was the DNA damage-associated protein Early Growth Response 1 (*EGR1*), with significant enrichments for 32 distinct LG4s observed in *EGR1* IPs. The TF with the second most LG4 enrichments was Specificity Protein 1 (*SP1*), which has previously been reported as associating with G4 promoter regions (67).

To further explore putative roles for LG4s in transcriptional regulation, we next examined their potential association with known, human-specific super-enhancers (SE) and super-enhancer elements (SEL). SEs are multi-enhancer clusters that are characterized by higher TF density and broader regulatory impact than typical enhancers. Using a comprehensive SE-SEL database containing 331 601 unique super-enhancers (89), we found that LG4 loci were 70.4% (±12.9%) more likely to overlap super enhancers and/or their elements than randomly selected, same-sized, control genomic loci (*n* = 3). Interestingly, only a subset (137/301) of LG4s are within close proximity to these regulatory clusters, while others are mostly intronic or in proximal promoters, which further signifies their importance to transcriptional regulation. To better examine the relationship between LG4s and enhancers, we next correlated our data with GeneHancer (90), a novel database of 285 000 candidate human enhancers (covering 12.4% of the genome) integrating a total of 434 000 reported enhancers from four different genome-wide databases: the Encyclopedia of DNA Elements (ENCODE), the Ensembl regulatory build, the functional annotation of the mammalian genome (FANTOM) project and the VISTA Enhancer Browser. Strikingly, 180 of our LG4 sequences had either fully or partially overlap with an annotated GeneHancer human enhancer (Supplementary Table S7) as compared to an average of only 84 overlaps (*n* = 5) between matched controls and enhancers.

### LG4s have increased small and large-scale genome variation

**Figure 4.**
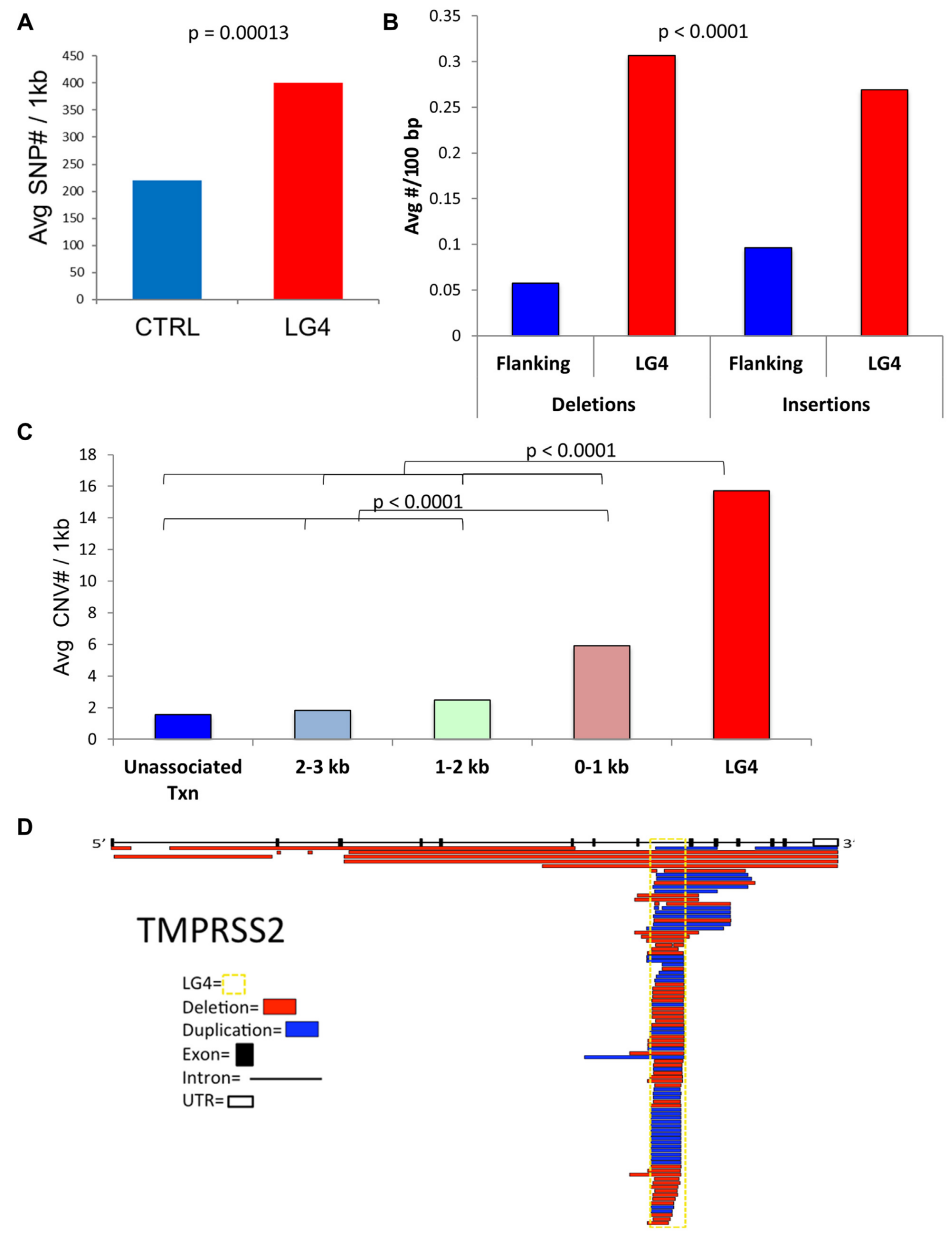
LG4s are associated with increased small and large-scale genome variation. (**A**) Entries from the dbSNP database for LG4s and size matched control regions. (**B**) Entries from the dbSNP database for LG4 and intronic regions directly surrounding LG4 (flanking). (**C**) CNV sizes from dbVAR and the average number of breakpoints/kb (y-axis) was calculated for each transcribed LG4, in 1kb increments away from LG4, and the rest of the transcript not directly associated with LG4 (Unassociated TXN). (**D**) Schematic diagram of the location and size of copy-number variants with respect to the predominant *TMPRSS*2 transcript. Introns, exons and UTRs denoted by lines, solid boxes, or open boxes, respectively. The yellow dashed box highlights the location of the *TMPRSS2* LG4 and individual duplications (blue boxes) and deletions (red boxes) are shown.

A recent analysis of single G4 motifs demonstrated enrichment for small nucleotide variations that include single nucleotide polymorphisms (SNPs) and insertions or deletions (indels) less than 50 bp, although the two were not distinguished in this study (29). SNPs have been shown to disrupt the regulatory ability of G4, indicating that regions prone to single base-pair changes have a high potential to loose regulatory ability (22,28). Using identified variations from genome-wide sequence studies available in the dbSNP database (50), the number of SNPs for each LG4 was counted and the average per 1000 bp calculated. There was a significant ∼82% enrichment (*P* < 0.00001) of SNPs in LG4s compared to randomly selected, size-matched control regions (Figure 4A, Supplementary Table S2), suggesting that LG4s, much like conventional G4 motifs (91), may be more prone to base damage or have a decreased ability to be accurately repaired. To determine if LG4s are similarly prone to indels, the average number of indels/100 bp for each transcribed LG4 and the surrounding non-exonic region was calculated separately from SNPs present in the dbSNP database. LG4 insertion events/100 bp were increased >5-fold and deletions >2.5-fold (*P* < 0.0001) as compared to surrounding non-exonic regions (Figure 4B). The observed increase in human genome variation (SNPs and indels) indicates that LG4s are prone to mutagenesis compared to surrounding loci.

In the human genome, copy-number variations (CNVs), which are defined as indels >50 bp, are also major contributors to genetic diversity and increase susceptibility to a range of genetic disorders (92,93). A CNV breakpoint is defined as the genomic location where a duplication or deletion occurs, and CNVs detected through genome-wide sequencing studies are available in dbVAR database at NCBI.org (51). We calculated the number of CNV breakpoints/kb for each transcribed LG4, 3 kb 5′ and 3′ of LG4 in 1 kb increments, as well as for the remaining transcript not associated with LG4 (referred to as unassociated transcripts). LG4 regions contained a significant (*P* < 0.0001), ∼10-fold increase in CNV breakpoints as compared to nearby unassociated transcripts, and an 8-fold increase relative to sequence >2 kb away (Figure 4C). Unexpectedly, regions within 1 kb of LG4 had a significant (*P* < 0.0001) ∼3-fold increase in CNVs over unassociated transcripts, suggesting that LG4s can invoke instability at proximal sequences (Figure 4C). This supports the findings of a previous report suggesting that DNA structures can induce mutagenesis in surrounding regions (94). Importantly our analysis provides evidence that many other transcribed G4 regions may be capable of repeat expansion and contraction. For example, a schematic representation of CNVs in oncogene *TMPRSS2* is shown in Figure 4D with deletions and duplications occurring throughout the LG4 sequence. Although how LG4 deletions and duplications affect *TMPRSS2* regulation is unknown, we note that *TMPRSS2* is the oncogene most frequently involved in gene fusions (41,95). That said, we find the majority of LG4 loci are similarly associated with annotated deletions and duplications (Supplementary Information S2).

### Deletions/duplications >10 bp accumulate within the *DIP2C* LG4 in yeast

The budding yeast *Saccharomyces cerevisiae* has been a powerful tool to study instability associated with G4 and other repetitive DNA sequences (32–34,91,93–104). Since our computational evidence indicated that indels and CNVs were elevated at LG4 loci in the human genome, we adapted a versatile *LYS2*-based frameshift reversion assay to directly assess the instability of a LG4 sequence in yeast. This system capitalizes on a 150 bp, functionally dispensable segment of the *LYS2* gene that is defined by stop codons in alternative reading frames. A frameshift mutation in this region can be reverted by any sequence addition/deletion of net opposite sign that occurs within the ‘reversion window’ demarcated by the stop codons (105). Prior studies demonstrated that most compensatory frameshifts are deletions/insertions of single base pairs in short, mononucleotide runs (57,105,106). Insertion of an out-of-frame LG4 sequence into the reversion window allows detection of additional mutation types specifically associated with G4-forming potential.

**Figure 5.**
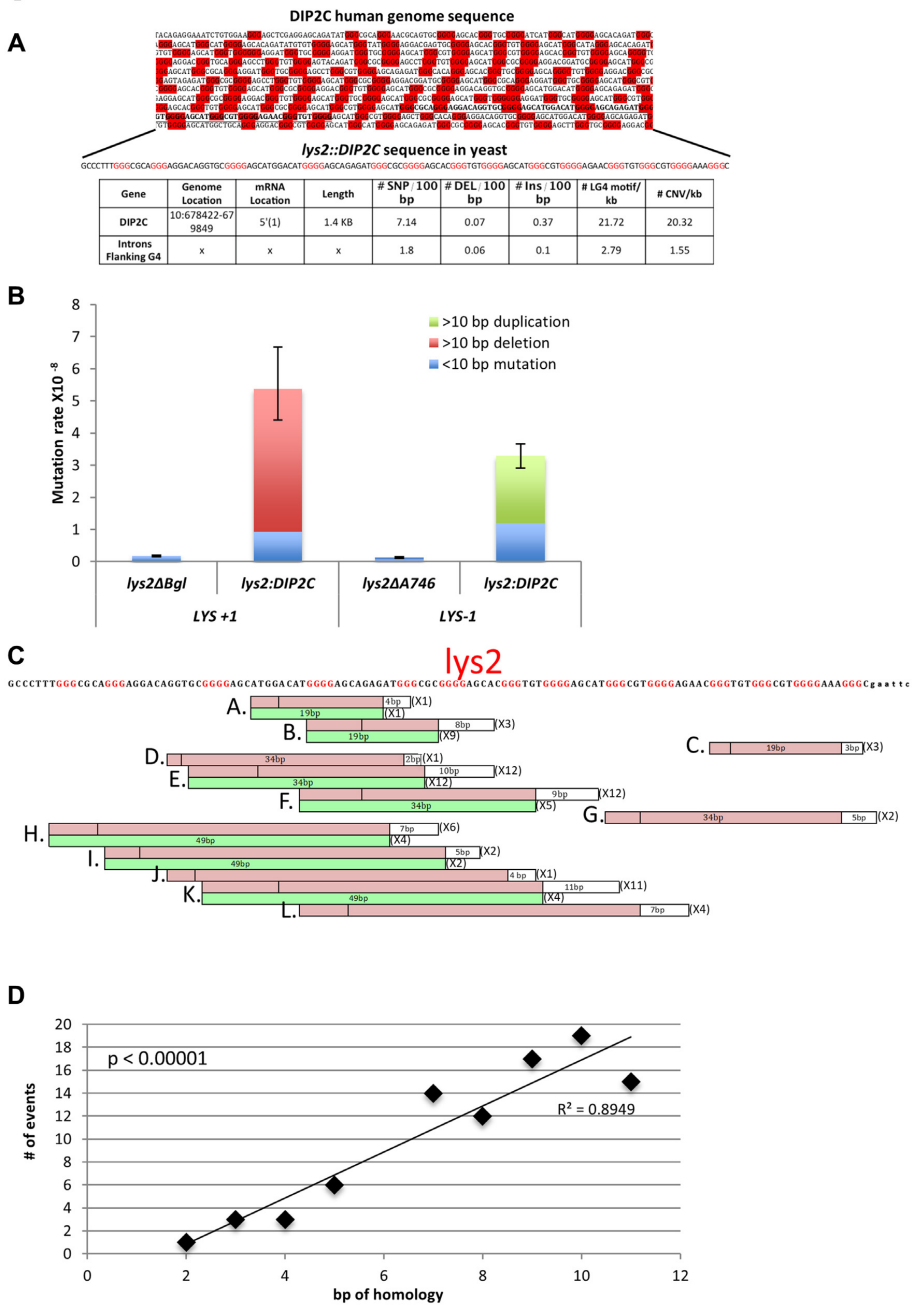
A *DIP2C* intronic LG4 is prone to deletions and duplications over 10 bp. (**A**) A 130 bp fragment of *DIP2C* LG4 was cloned into yeast. The G-repeats are in red. A table of LG4s genetic traits and human genome variation compared to non-exon regions surrounding all LG4s is below. (**B**) The LG4 *LYS2* reversion window was sequenced for revertants and rates adjusted by proportion of a given mutation type. Error bars are 95% confidence intervals and adjusted for the total rate. (**C**) Mutation spectrum for deletions (red bars) and duplications (green bars) with corresponding size and number detected for both Lys+1 sequences (top) and Lys −1 (bottom). (**D**) The bp of perfect homology at the end of duplications/deletions.

The *DIP2C* sequence (Figure 5A) was selected for this analysis because of our *in vitro* data confirmed its potential to form G4 structures and our computational analysis identified its significant association with indels and CNVs. Although over 800 bp of G-rich repetitive sequence have previously been inserted into *LYS2* (107), we were unable to clone the full-length *DIP2C* sequence into bacterial plasmids and, therefore, used a 130 bp segment representative of the LG4 (Figure 5A). The *DIP2C* insertions created a +1 or −1 *lys2* frameshift mutation (*lys2::DIP2C+1* and *lys2::DIP2C-1* alleles, respectively) that revert by net −1 or +1 changes in the extended ∼280 bp reversion window.

The reversion rates of the *lys2::DIP2C* frameshift alleles were measured and compared to those of *lys2* +1 and −1 alleles without the *DIP2C* insertion. Although addition of the *DIP2C* sequence roughly doubled the size of the theoretical reversion window where compensatory frameshift mutations can be detected, the reversion rates of the *lys2::DIP2C+1* and *lys2::DIP2C-1* alleles were elevated 15–20-fold relative to relative to alleles without the *DIP2C* insertion (Figure 5B). The region of the reporter containing the *DIP2C* insertion was sequenced to determine the types of compensatory frameshift mutations that occurred. Approximately 85% of the *lys2::DIP2C+1* revertants (53/64) contained deletions >10 bp, while ∼65% of *lys2::DIP2C-1* revertants (37/58) were duplications >10 bp. These deletions/duplications are summarized in Figure 5C where the size and number of each are indicated (see also Supplementary Table S8). Importantly, all events had at least 2 bp of perfect homology (a direct repeat) at the endpoints. The largest flanking repeat was 11 bp and in general, there was a correlation between the number of times a given event was detected and the length of the endpoint homology (Figure 5D). For deletions, one copy of the repeat and the intervening sequence was deleted; for duplications, one copy of the repeat plus the intervening sequencing was duplicated. There were 12 distinct events (A–L) among the 53 deletions detected; among the 37 duplications, there were six distinct types (Figure 5C). Each of the duplications had a corresponding deletion event with the same endpoints, and these were given the same letter designation as the deletion. Finally, all but one event within this initial sample of deletions/duplications was either 19, 34 or 49 bp. This striking periodicity of 15 bp reflects both the repetitive structure of *DIP2C* as well as the constraint to delete a non-multiple of 3 bp in order to restore the *LYS2* reading frame. Although similar deletions/duplications can be generated during the repair of double-strand breaks (108), neither loss of homologous recombination nor nonhomologous end-joining pathway affected their rates (*rad51Δ* and *dnl4Δ* backgrounds, respectively; Supplementary Table S8). This suggests that slippage between the flanking direct repeats during DNA replication is the most likely cause of the large deletion/duplications in the *DIP2C* sequence (108,109). That said, although we find sequence repetitiveness rather than the ability to form G4 structures is typically the principle driver of deletions and duplications in this sequence, we have extensively examined the relationship between G4 formation and LG4 mutagenesis formation (Supplementary Information S3) and find that over half of all duplication events occurring during periods of high transcriptional activity are likely directly attributable to G4 structure.

### LG4s are significantly associated with genomic rearrangements

**Figure 6.**
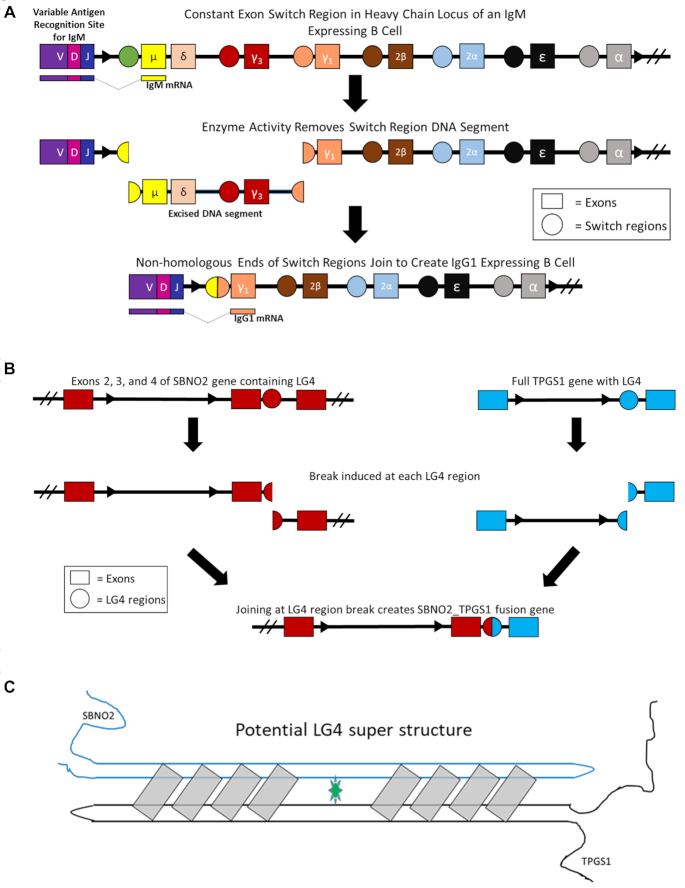
Recurrent translocations between LG4s. (**A**) Mechanism of isotype switching in activated B cells. Resulting mRNA indicated below genomic sequences. V; variable exon. D; diversity exon. J; joining exon. IgM; immunoglobulin M. IgG1; immunoglobulin G subclass 1. μ; immunoglobulin M heavy chain exon. δ; immunoglobulin D heavy chain exon. γ3; immunoglobulin G subclass 3 heavy chain exon. γ1;immunoglobulin G subclass 3 heavy chain exon. ϵ; immunoglobulin E heavy chain exon. α; immunoglobulin A heavy chain exon. 2β; immunoglobulin G subclass 2 beta heavy chain. 2α; immunoglobulin G subclass 2 alpha heavy chain (32,52,127–130). (**B**) Gene fusion (40,41) resulting from distinct LG4 breaks independently reported in breast (TCGA-BRCA) and cervical (TCGA-CESC) malignancies. SBNO2; Strawberry notch homolog 2. TPGS1; Tubulin Polyglutamylase Complex Subunit 1. (**C**) Model of potential LG4 super structure.

Having confirmed LG4s are associated with increased small and large-scale genomic variation, we next examined if LG4s are similarly associated with mutation in malignancy through mining the somatic mutation information available in COSMIC (Catalogue of Somatic Mutations in Cancer) (40). The COSMIC database contains genome-wide mutational data from over 32 000 cancer genomes derived from peer-reviewed, large-scale genome screening datasets and other databases such as TCGA and ICGC (40). Notably, the identified LG4s are 31 times closer to chromosomal breakpoints contained within the COSMIC dataset than matched controls (Supplementary Table S2). In addition to this, we also screened genes containing LG4s against the FusionGDB (41). FusionGDB is a publicly available database consolidating data from three primary fusion gene resources: chimeric transcripts and RNA-seq data (ChiTaRS 3.1), TumorFusions, and fusions identified in The Cancer Genome Atlas (TCGA). In all, the FusionGDB contains over 48 000 unique gene fusions characterized from an array of malignancies. Strikingly, 144 of the 185 LG4s located in annotated protein-coding loci were in genes involved in fusions listed in the FusionGDB (*P* < 0.001) (Supplementary Table S2). Thirty-seven of the chromosomal breaks contributing to annotated FusionGDB gene fusions occurred within or in close proximity to an LG4 present in the *TMPRSS2* locus (Figure 4D). Gene fusions involving *TMPRSS2* are the most frequently reported across all malignancies, with approximately half of all prostate cancers containing *TMPRSS2-ERG* fusions (110). There were additionally 23 unique gene fusions annotated in the FusionGDB that corresponded to genomic rearrangements occurring between distinct LG4 sequences resembling rearrangements occurring between long G4-capable-motif dense switch regions during mammalian immunoglobulin class switch recombination (Figure 6A). As an example, a genomic rearrangement occurring between LG4s found in the *SBNO2* and *TPGS1* loci has been repeatedly observed in various malignancies (Figure 6B) (41,95). In light of the frequent participation of LG4s in chromosomal translocations, it is tempting to speculate that G4 sequences may directly facilitate trans interactions (Figure 6C).

### Neighboring LG4 loops are frequently complementary and base pair *in vitro*

**Figure 7.**
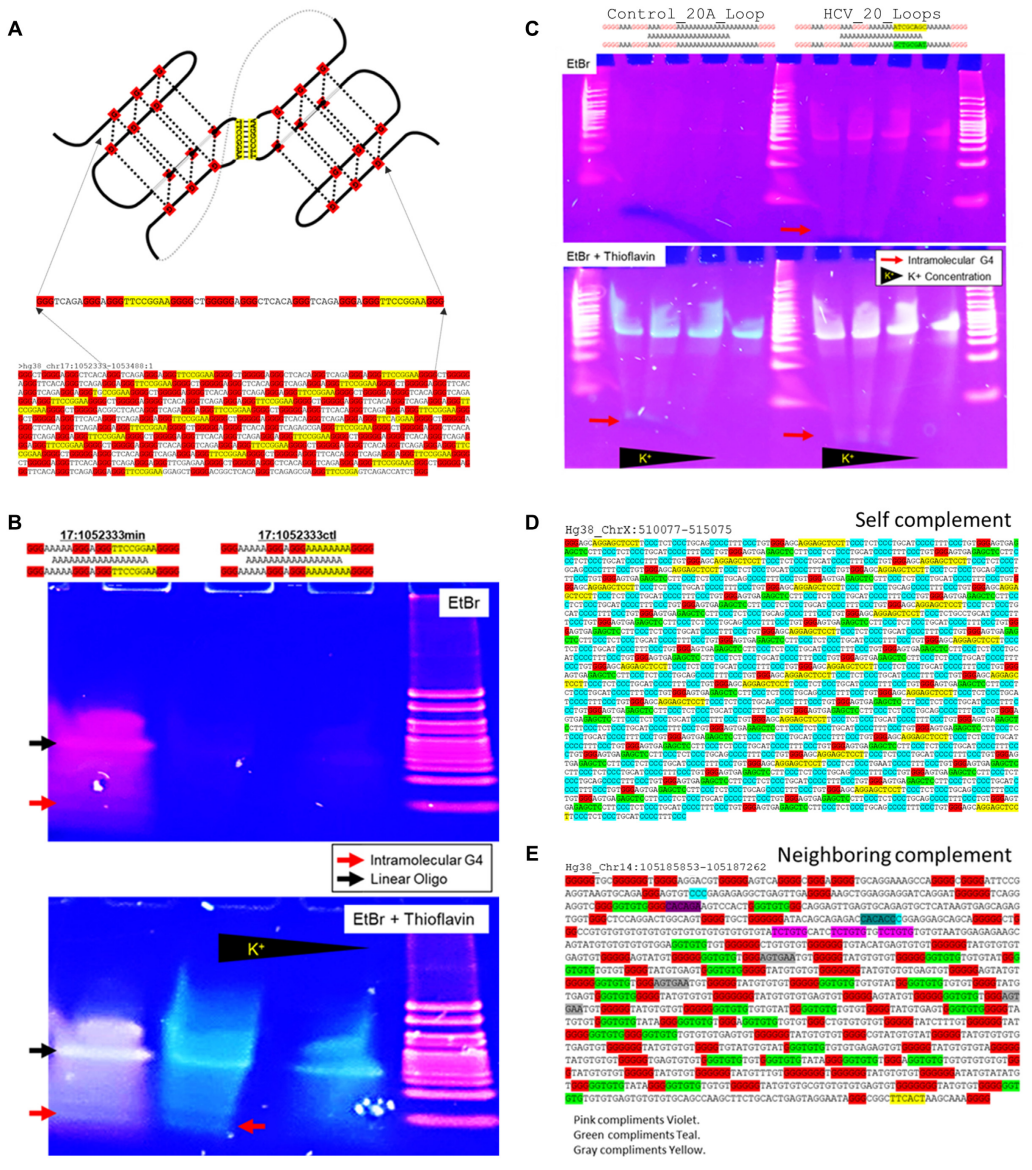
LG4 Kissing Loops. (**A**) Predicted model of neighboring G4 loop interaction in human LG4 locus occurring at hg38:chr17:1052333–1053488 is illustrated. Complementary loops are highlighted in yellow. Guanine triplets are highlighted in red. (**B**) Nondenaturing G4 gel electrophoresis of minimal G4 capable sequence (taken from the LG4 detailed in **A**). Lane 1 – tandem of two minimal G4 capable sequences with complementary loops. Lanes 2 and 3 – tandem of two minimal G4 capable sequences with complementary loops replaced by adenosines. Upper gel image: EtBr stain (orange) only. The affinity for EtBr binding of dsDNA is ∼25 times its affinity for ssDNA. Lower gel image: Subsequent Thioflavin (blue) staining of the identical gel shown in the upper image. (**C**) Nondenaturing G4 gel electrophoresis of G4 capable sequences with complementary loops from known HPV kissing hairpins. (Left) Control oligonucleotide lacking complementary loops. (Right) Oligonucleotide containing complementary loops known to participate in HCV hairpin kissing (111). Upper gel image: EtBr stain (orange) only. Lower gel image: Subsequent Thioflavin (blue) staining of the identical gel shown in the upper image. (**D**) Additional example of a LG4 locus with loops described as ‘Self complements’ (like the LG4 in A). (**E**) Example of a LG4 locus with loops described as ‘Neighboring complements’.

A detailed inspection of individual LG4 sequences revealed single-strand loops of neighboring G4s within individual LG4 regions that were frequently complementary to one another (Figure 7A, Supplementary Table S2, Supplementary Information 4). Whereas the complement to a 6 nt loop would only be expected to occur (at random) once every 4,096 bp and the average LG4 length is only 1843 bp, 178 of the 301 individual LG4 sequences we identified contained internal G4-loop complementarities. Each of these 178 loci had, on average, 34.8 complementary loops of 8.2 bp in length. We hypothesized that G4 loops within individual LG4 loci directly pair with one another in a manner similar to numerous, well-documented kissing stem–loop interactions characterized in various RNA (111,112) and (less frequently) DNA structures (113,114) (Figure 7A). To examine whether neighboring G4 loops can base pair with one another *in vitro* via a loop:loop kissing interaction (termed a G4 Kiss or G4K), we synthesized oligonucleotides containing two minimal G4-capable sequences separated by a polyA linker. Oligonucleotides contained either (i) neighboring G4-capable sequences with putative kissing loops from the LG4 shown in **A** (Figure 7B), (ii) two minimal G4-capable sequences separated by a polyA linker with loops containing characterized viral kissing-loop complements (Figure 7C) or (iii) controls for each of these that lacked complementarity. These oligonucleotides were evaluated by nondenaturing G4 gel electrophoresis and sequential staining (as assessed in Supplementary Information 5). Importantly, the affinity of ethidium bromide (EtBr) for dsDNA is 25 times greater than its affinity for ssDNA, while Thioflavin T associates with G4 DNA but not normal duplex DNA (115,116). Sequential EtBr and Thioflavin staining (pink and blue, respectively) confirmed that an intramolecular G4 structure formed by a DNA oligonucleotide with complementary loops directly engages in an observable double-strand interaction (upper gels) whereas the G4 structure formed by nearly identical control oligos lacking complementary loops does not (Figures 7B, C). Furthermore, we found that LG4s containing complementary loops typically assume (and can be fairly evenly divided between) one of two general configurations. The first of these consists of a regularly repeating series of self-complementary loops ‘self complementary’ (Figure 7D, Supplementary Information S4). In contrast, ‘neighboring complementary’ LG4s are generally much less organized but also more complex in that they typically contain several different putative loop:loop interactions (Figure 7E, Supplementary Information S4). Together, these findings suggest LG4s adopt a novel, higher order, composite G4K structure potentially driving the formation and/or maintenance of these conspicuous genomic regions.

## DISCUSSION

We have developed a novel algorithm for identifying long stretches of tandem G4s resembling Ig switch regions in genomic sequences, LG4ID, and used it to identify 301 LG4 regions averaging over 1.5 kb in length in the human genome. Subsequent analyses of these LG4s suggest that these unusually large, G4-dense regions are capable of complex G4 formation and possess a high probability of regulatory and mutagenic potential. Notably, shorter LG4 regions (e.g. 200–1500 bp), although smaller than the *Sμ* or *Sγ3* G4-containing immunoglobulin switch regions, might also be biologically relevant, and we suggest it would be interesting to determine if shorter LG4s are similarly located in regions with regulatory potential and subject to increased genomic variation. That said, we find over 6,000 human genomic loci between 500 and 1500 bp in length with a G-triplet/sequence length ratio equal to or greater than that used in the current search. As the number of LG4 calls decreases dramatically as search window size increases (as detailed in Supplementary Table S9), we elected to focus the current study on LG4s 1500 bp and greater in length with a density of at least 8 GGGs per 100 bp (as the core *Sμ* G4-containing immunoglobulin switch region is 1489 bp in length with a GGG density of ∼8.0 GGGs per 100 bp (Supplementary Information S5). Importantly, the flexibility of LG4ID allows window size (total length) to be reduced while maintaining a constant G-triplet/sequence length ratio, or alternatively, for the density of G-triplets to be adjusted, and as such, have made the LG4ID source code available for download on the LG4ID website to allow users to explore manipulating these parameters.

Although the present search focused on identifying regions of G4 formation, we find several LG4s are capable of assuming other non-B form DNA structures, forming stable hairpins, and/or contain long purine repeats (ex AGGGA) capable of triplex structures (data not shown) (117). We additionally found that a subset of cloned LG4 motifs stalled Klenow polymerase in a K^+^ independent manner, suggesting that Li^+^ is capable of stabilizing G4, or conversely, other G-rich structures are formed in addition to G4. Although stalling was enhanced by K^+^ and confined to the G-rich strand and CD scans demonstrated parallel G4 formation (Figure 3), non-G4 structures may also form from LG4 sequences. Hairpins, for instance, can also stall replication (118). Since the LG4 sequences that showed stalling during Klenow extension in Li^+^ did not contain any long stretches of purines, it is unlikely that this was due to triplex DNA. K^+^-independent stalling was also reported in telomeres, and to date, details of an alternative structure to G4 formation remain elusive (119). Further analysis of the identified LG4 regions will be needed to determine what sequence requirements, if any, lead to K^+^-independent stalling on only the G-rich strand.

We found that LG4s were associated with both sequence variations and known promoter/enhancer elements, suggesting a connection between G4 structures, site-specific instability, and genetic regulation. As the mechanism(s) of increased SNPs and indels at LG4s cannot be deciphered from computational data, and were not found to be elevated in our *DIP2C* yeast studies, more direct experimentation will be needed to delineate the role(s) of G4 structures in LG4 mutagenesis. The use of only a 130 bp portion of *DIP2C* in the yeast studies may have resulted in a significant underrepresentation of the true mutagenic capacity of the intact LG4, although duplications and deletions reflecting the repetitive nature of the sequence were evident. It should also be noted that the LG4s identified in this work contain a wide variety of repeat sequence compositions (Supplementary Table S4), and we predict that the mutagenic propensity and genetic control of instability will likely differ between distinct LG4s.

Strikingly, 119 of 185 protein-coding genes that contain regulatory LG4s have known disease associations (as indicated in Supplementary Table S2). Intergenic LG4s may also be disproportionately associated with disease, as the disruption of proper gene regulation due to CNVs, SNPs or indels in regulatory LG4s could contribute to cellular dysfunction and/or disease. For example, overexpression of Anoctamin 9, *ANO9*, is associated with the progression of metastatic colorectal cancer (120). The *ANO9* LG4 is a site for *EGR1* transcription factor interactions, suggesting this locus is highly involved in gene regulation. Further, the ANO9 LG4 is significantly enriched for CNVs, SNPs and indels making it possible that an increase of site-specific mutagenesis could disrupt proper *ANO9* regulation leading to the progression of late-stage colorectal cancer. Considering most genome-wide association sequencing studies of human disease have historically focused on coding DNA, mutations in regulatory non-coding regions, such as LG4s, could provide previously missed insights into the etiology of multiple diseases.

**Figure 8.**
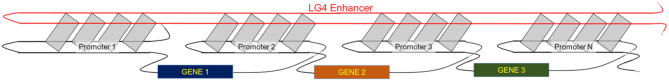
LG4 enhancer model. Potential mechanism by which LG4s could interact with multiple gene promoters to coordinate their expressions.

Importantly, in addition to high levels of CNVs, SNPs, and indels being associated with LG4s, translocation hotspots have also previously been reported to be enriched at sites harboring G4-capable sequences (30). In agreement with this, we similarly found translocations and gene fusions associated with LG4s (Figure 6, Supplementary Table S2). As such, it is tempting to speculate that interchromosomal and long-range intrachromosomal hybrid G4 formation between LG4 sequences may directly facilitate recurrent translocations (Figure 6B, C). Potentially related to this, we found that 217 of the 301 LG4 loci identified in this study either fully or partially overlap with an annotated human enhancer (Supplementary Tables S2 and S7) (versus only 84 average overlaps (*n* = 5) between size and nucleotide composition matched control loci and enhancers). Our preliminary analyses of the gene promoters likely regulated by these enhancers found them significantly enriched with G4-capable sequences (data not shown) suggesting that LG4 sequences could potentially form hybrid G4s with distal promoters with a LG4 and interacting promoter each contributing half the sequence necessary to form a composite G4. Notably, in 2015, a similar model for G4-based promoter:enhancer interaction was proposed based on the identification of a marked propensity for single promoter:enhancer pairs to contain potentially interacting minimal G4 motif components (121). That said, we find the average number of G triplets (GGG) potentially contributing to hybrid G4 formation within a single LG4 is 74 times the number of available G triplets found in the average inferred target-gene promoter. This leads us to speculate that the high number of available G4 donor sequences allows LG4 enhancers to act as long ‘Velcro-like’ regions that simultaneously interact with a number of neighboring gene promoters coordinating their expressions (Figure 8). Excitingly, a thorough comparison of LG4 positions with two distinct UCSC Hi-C data set tracks (42–44) finds LG4s involved in >200× more suspected inter-chromosomal interactions than sized matched controls with 321 943 interactions overlapping with LG4s versus only 1585 interactions overlapping with controls (Supplementary Table S10). As such, we suggest the clear propensity for LG4s to participate in distal chromosomal contacts well aligns with their potential to serve as functional enhancers.

Finally of note, interactions (based on cooperative unfolding) between neighboring G4s in the human ILPR promoter have been previously reported (122), and human telomeric G4s have been suggested to assume a higher-order structure stabilized by loop:loop interactions between neighboring minimal G4s (123–125). Similar to these previously suggested interactions between neighboring G4s, our results provide strong evidence that LG4s adopt a previously undescribed, higher order, G4-based secondary structure we have termed a ‘G4 Kiss or G4K’. Strikingly, a detailed inspection of individual LG4 sequences found that single-stranded loops of neighboring G4s within individual LG4 regions are frequently complementary to one another (Figure 7A, Supplementary Table S2, Supplementary Information S4). Whereas the complement to a 6 nt loop would only be expected to occur (at random) once every 4096 bp and the average LG4 length is only 1843 bp, we found that 178 of the 301 LG4 sequences identified contain extensive internal G4 loop complementarities, with each containing (on average) 34.8 complementary loops of 8.2 bp in length. Although loop composition is clearly not random (126), making it more difficult to predict the exact likelihood of the occurrence of a 6 nt loop complement within a given LG4, we suggest the fact that single-strand loops of neighboring G4s within individual LG4 regions are frequently, strikingly complementary is undeniable (Supplementary Information S4) leading us to hypothesize, then experimentally confirm *in vitro*, that neighboring G4 loops base pair with one another via a loop:loop kissing interaction or G4K (Figure 7B, C) similar to characterized kissing interactions between both RNA (111,112) and DNA (113,114) stem–loops (Figure 7A). Together, these findings indicate that LG4s adopt a novel, higher order, composite G4K structure that potentially drives the formation and/or maintenance of these conspicuous genomic regions.

In summary, we developed then utilized the novel LG4ID program to obtain a comprehensive database of LG4 regions in the human genome. This allowed us to detail LG4 features such as sequence composition, genetic orientation, length, location, G4 density and small sequence variations; characterize structural rearrangements, regulatory capacity, genomic context and associated genes; examine the ability to form higher order structures; and correlate LG4 presence with human disease (Supplementary Table S2). Our findings add to a growing body of research demonstrating that sequences capable of G4 formation are inherently unstable and involved in regulatory functions. Further study is needed to determine roles for individual LG4s in human disease, to examine the mechanism by which G4s in LG4 enhancers participate in and facilitate promoter interactions, and to better characterize and determine endogenous functions of the novel ‘G4 Kiss’ DNA structure identified in this work.

## DATA AVAILABILITY

LG4 search program available for download at http://omnisearch.socsouthalabama.edu:8080/g4search#.

## Supplementary Material

gkaa357_Supplemental_Files
