# Supplementary material for: Characterization of long G4-rich enhancer-associated genomic regions engaging in a novel loop:loop ‘G4 Kissing’ interaction
Source: Nucleic Acids Res. 2020 May 8;48(11):5907–25. doi: 10.1093/nar/gkaa357 (PMC7293029; doi:10.1093/nar/gkaa357)

Supplementary Files

Characterization of long G4-rich enhancer-associated genomic regions engaging in a novel loop:loop "G4 Kissing" interaction

Jonathan D Williams[1,2,§], Dominika Houserova[3,§], Bradley R Johnson[2], Brad Dyniewski[2], Alexandra Berroyer[2], Hannah French[2], Addison A Barchie[4], Dakota D Bilbrey[4], Jeffrey D Demeis[4], Kanesha R Ghee[4], Alexandra G Hughes[4], Naden W Kreitz[4], Cameron H McInnis[4], Susanna C Pudner[4], Monica N Reeves[4], Ashlyn N Stahly[4], Ana Turcu[4], Brianna C Watters[4], Grant T Daly[3], Raymond J Langley[3], Mark N Gillespie[3], Aishwarya Prakash[3,5], Erik D Larson[2,6], Mohan V Kasukurthi[7], Jingshan Huang[7], Sue Jinks-Robertson[1], and Glen M Borchert[3,4,*]

[1] Department of Molecular Genetics and Microbiology, Duke University, Durham, NC, 27708, USA
[2] School of Biological Sciences, Illinois State University, Normal, IL, 61790, USA
[3] Department of Pharmacology, University of South Alabama, Mobile, AL, 36688, USA
[4] Department of Biology, University of South Alabama, Mobile, AL, 36688, USA
[5] Department of Biochemistry and Molecular Biology, University of South Alabama, Mitchell Cancer Institute, Mobile, AL, 36688, USA
[6] Department of Biomedical Sciences, Western Michigan University Homer Stryker MD School of Medicine, Kalamazoo, MI, 49007, USA
[7] School of Computing, University of South Alabama, Mobile, AL, 36688, USA

* To whom correspondence should be addressed. Tel: +1 251 461 1367; Email: borchert@southalabama.edu

[§] The authors wish it to be known that, in their opinion, the first two authors should be regarded as joint First Authors.

Supplementary File Legends

Supplementary Table 1. The number of LG4 loci found in the human genome with respect to known genes. Raw numbers used in Figure 2B percentage calculation for LG4 (# LG4 loci) and control loci (# CTRL loci) overlapping with or within 5kb of a known gene (Known Gene-associated), overlapping with or within 5kb of a GENSCAN predicted gene (Predicted Gene-associated), or unassociated with gene transcripts (Unassociated) are shown.

Supplementary Table 2. LG4 master list. Genomic location (Hg38), GGG density, SNP density, Ensembl regulatory build annotations, presence and number of G4 loops potentially engaging in local kissing structures, and all overlapping genes (as well as genes within 5kb 5' or 3') are indicated along with their potential involvement in human disease(47) / characterized breakpoints. Strand occupancy (positive or negative) is also detailed for each LG4 and associated gene.

Supplementary Table 3. DAVID analysis of chromosomal location for LG4 genes. (**2.1**) Chromosomes (left) containing significant enrichment for LG4 genes (right) are shown. (**2.2**) Cytobands and the chromosome they are found on (left) containing significant enrichment for LG4 genes (right) are shown.

Supplementary Table 4. Oligonucleotides tested by circular dichroism. LG4 sequences used to design oligonucleotide are listed (LG4 name) and corresponding sequence of oligonucleotide assayed (oligo sequence). The different G repeats involved in intra-molecular G4 are highlighted in different colors for each corresponding G-repeat length (bottom).

Supplementary Table 5. Sequences representing LG4s ranging from 130-1300 bp cloned into a vector for single stranded DNA production. Sequences assayed are shown with G-repeats >3 bp bolded.

Supplementary Table 6. LG4 regulatory sequence associations. (**6.1**) A subset of regulatory motifs contained ChIP-Seq pull down interactions (ChIP-Seq loci) with some LG4 sequences having multiple ChIP-Seq interactions represented by more TFs pulled down than ChIP-Seq loci. All TFs pull down at LG4 or control loci are shown below the number of different types of TFs (# types). Gray boxes highlight the TFs with the largest LG4 increase over control(21, 86, 130). (**6.2**) LG4 are enriched regulatory regions. The number of regulatory LG4 or control (Ctrl) loci identified on Ensembl regulatory build and the total number searched are indicated. (**6.3**) Distribution of regulatory elements broken down by promoter, transcription factor binding sites (TFBS), transcriptional repressor CTCF, promoter flanking, enhancer, or open chromatin (OC). (**6.4**). Significant increases in LG4 TF pull downs over control loci are listed below the number of different types of TFs (# types).

Supplementary Table 7. Overlapping LG4/GeneHancer Enhancers. The 180 LG4s found to overlap with annotated GeneHancer Enhancers and corresponding enhancers are listed. Full GeneHancer entries, chromosomal positions for LG4s and enhancers, and length of overlap are indicated.

Supplementary Table 8. *DIP2C* Lys2 reversion rates and sequencing data.

Supplementary Table 9. Effects of altering LG4ID parameters. Number of LG4ID LG4 calls for Human Chromosome 21 requiring 4, 8, or 12 GGG / 100 bp densities within 250, 500 or 1,500 bp windows. By default, LG4ID requires 8 GGGs / 100 bp within a 1,500 bp window.

Supplementary Table 10. LG4 Hi-C interactions. List of chromosomal interactions between LG4 loci (and control sequences) and other genomic locations. Interaction data was obtained from UCSC Genome Browser using Hi-C and

Micro-C track. Columns are as follow: Chrom=Chromosome (or contig, scaffold, etc.). For interchromosomal, use 2 records; chromStart=Start position of lower region. For chromosomal, set to chromStart of this region; chromEnd=End position of upper region. For interchromsomal, set to chromEnd of this region; name=Name or ID of item. Usually name1/name2 or name1/name2/exp or empty; score=Score from 0-1000, typically derived from value; value=Strength of interaction or other data value; exp=Experiment name for filtering. Use . if not applicable; color=Item color, as itemRgb in bed9. Typically based on value or exp; sourceChrom=Chromosome of source region (directional) or lower region. For non-directional interchromosomal, chrom of this region; sourceStart=Start position of source/lower/this region; sourceEnd=End position of source/lower/this region; sourceName=Identifier of source/lower/this region; sourceStrand=Orientation of source/lower/this region: + or -. Use . if not applicable; targetChrom=Chromosome of target region (directional) or upper region. For non-directional interchromosomal, chrom of other region; targetStart=Start position of target/upper/this region; targetEnd=End position of target/upper/this region; targetName=Identifier of target/upper/this region; targetStrand=Orientation of target/upper/this region: + or -. Use . if not applicable.

Supplementary Information 1. LG4ID graphical algorithm workflow, summary, and training.

Supplementary Information 2. Occurrence of annotated structural variations at LG4 loci. Ten additional LG4 loci were selected then visualized in the UCSC browser after being zoomed out 10x making the central 10% of each window correspond to reported LG4 positions. The “database of structural variants” track listing (CNVs, Inversions, Insertions and Deletions) was selected.

Supplementary Information 3. Detailed analysis of the role of G4 in *DIP2C* intronic LG4 mutation.

Supplementary Information 4. Potential G4 Kissing Loops detailed in 20 select LG4s. Chromosomal position and potentially interacting complementary loops are indicated.

Supplementary Information 5. Nondenaturing G4 gel electrophoresis and sequential staining. (**A**) G4 formation of G4-capable oligonucleotides with third loop lengths of 3, 10, and 20 nts. (**B**) G4 formation

Supplementary Table 1

	# LG4 loci	# CTRL loci
Known Gene-associated*	234	102
Predicted Gene-associated	42	61
Unassociated	25	138
total	301	301

*p < 0.00001

Supplementary Table 2. LG4 master list. Genomic location (Hg38), GGG density, SNP density, Ensembl regulatory build annotations, presence and number of G4 loops potentially engaging in local kissing structures, and all overlapping genes (as well as genes within 5kb 5' or 3') are indicated along with their potential involvement in human disease(47) / characterized breakpoints. Strand occupancy (positive or negative) is also detailed for each LG4 and associated gene.

INCLUDED AS SEPARATE EXCEL SPREADSHEET

Supplementary Table 3.1

Chromosome	p-Value	Genes
Chromosome 19	0.000000002	ABCA7, VSTM2B, NMRK2, TNFSF9, CACNG8, TMPRSS9, HSD17B14, MBOAT7, TPRX1, IGLON5, DIRAS1, TCF3 SBNO2, TPGS1, SHANK1, BTBD2, THEG, CNN2, PTPRH, CDC42EP5, MAP2K2, TNNI3, R3HDM4, CAMSAP3 CBLC, C2CD4C, MED16, MADCAM1, HCN2, SHC2, ZBTB45, ARHGEF1, KLK7, KLHL26, KIF1A76, NWD1
Chromosome 16	0.03	SNX29, PRKCB, CPNE7, SPIRE2, ZFPM1, GALNS, DPEP1, TCF25, UBE2I, LMF1, HBM, NPIPA1, GSG1L, LUC7L

Supplementary Table 3.2

Cytoband	p-Value	Genes
19p13.3	0.000000000001	ABCA7, MAP2K2, NMRK2, R3HDM4, TNFSF9, TMPRSS9, C2CD4C, MED16, DIRAS1, SBNO2, TCF3, MADCAM1, HCN2, SHC2, TPGS1, BTBD2, CNN2
12q24.33	0.0001	GALNT9, PIWIL1, DDX51, FBRSL1, PXMP2
10q26.3	0.0003	C10orf91, LRRC27, CALY, SPRN, PRAP1
17q25.3	0.003	TEX19, B3GNTL1, CCDC57, DNAH17, CANT1
16q24.3	0.003	CPNE7, GALNS, DPEP1, TCF25
10p15.3	0.003	LARP4B, ADARB2, DIP2C
17p13.3	0.004	RPH3AL, P2RX5, TUSC5, ABR, DOC2B
11p15.5	0.004	B4GALNT4, IGF2, BET1L, DUSP8, ANO9
20q13.33	0.01	PCMTD2, OPRL1, HRH3, ZBTB46
2q37.3	0.01	CROCC2, GAL3ST2, KIF1A, D2HGDH
Xp22.3; Yp11.3	0.01	ASMTL, CRLF2
19q13.4	0.01	PTPRH, TNNI3, CACNG8, MBOAT7
18q23	0.01	CTDP1, NFATC1, ZNF516
22q13.33	0.02	CRELD2, TTLL8, PPP6R2
8q24.3	0.02	RHPN1, LY6H, GPIHBP1, MROH1
9q34.3	0.02	GRIN1, EXD3, LCN15, ENTPD8
13q34	0.03	TMCO3, RASA3, F7
5p15.3	0.05	AHRR, NKD2

Supplementary Table 4

LG4 name	Oligo Sequence
CTCFL	CAGGGGGGAAGGGGAGTAGGGAGGGGGAGGGGGAG
DIP2C	TGGGCGCGGGGAGCACGGGTGTGGGGAGCATGGGCGTGGGGA
F7	GTGGGGGATGGGGTGTGTGGGGTGTCGGGGAT
H2CN	CAGGGGTGGGGCTATGGGAGATCTGGGTGGGGTCAAGGCCCCAGGGATGGGGCT
MLF1	AGGGTGTGAGGTGAGGGGAAGGGTTGAGGGCGTGAGGTGAGGGG
MXI1	GAGGGGAGGGGCGTGTGAGGGGAGGGGAG
NACA	AGCTGGGGGAGTGGGGGCCCCTTTGGGGGGTGGGGTA
P2RXS	CTGGGCGGGGGGCGGGGGACGTTGGGAGGGTCCCTGGGGGCCCCGCT
PDE6B	GTGGGGTAGTGGGAGCAGCATGGGGTAGGGGAATAGCGTGGGGTA
PRDM16	GAGGGGTGTGTTGGGAGGGGTGAGTTGGGGGGGTGCACCGGGAGGGGTG
SARDH	GAGGGAGGGTGAGGGAGAGGGAGGGTGAGGGAGAGGGAGGGTGAGGGGA
TMPRSS2	GAGGGGGCGAGGGGGTGAGTGAGGGGGCGAGGGGGTG
TP73	CAGGGCTGGGATTGCGGGAGGAGGGGC
TPRX1	CAGGGAGTGGGCCTGGGATCTGGGCTGGGCCTGGGATTGGGGCAGGGA
UBE21	GAGGGAGGGAGGGAATGAGGGAGGGAGGGAAT
G=triplet G=quadruplet G=quintuplet G=sextuplet	

Supplementary Table 5

LG4	Sequence
>Sγ3	GCCCTTAACAGTGGACCGAGTGACGTTGCTGCCCCAGGTTGGGGGAGGGCAGAGTGAGCAGGGGGCAGGCATTGGAGCTCAGGGACCAGGACCAAGCAGCCACAGGTGCACAGGGCAGGTGGGAGGCAGGAAGAACAGGGGGCAGCTCCTGGAGCTCAGGGGACCACGGGAGAGCATCTGAAGGTGAACAGGAGCCGGTGGAGGGGCAGGATGAGCAGGAGGAAGACCCTGGAGCTCAGGGGACCAGGGCAGAGCAGCCACAGGTGAGGAGGCAGCCACAGGTGCGCAGGGCAGGTGGGAGGCAGGACCAGCAGGGGGCAGCCCCTGGAGCTCATAGAGCCAGGGCAGAGCAGTCGCAGGTGAGCAGGGCTCAGTGGAAGATGGGCAAGCAGGTGGCAGCTCCTGGAGCTCAGGGCACCAGGGCAGTGTAGCCACAGGTGAGCAGGGGCTGGTGGGAGGCAGGACAAGCAGGCGGCAGCTCCCGGAGCTCAGGGAACCAGGGCAGTGTAGCTGCAGGTGAGCAGGGGCTGGTGGAAGGCAGGACAAGCAGGTGGCAGCTCCNGGAGCTAAGGGC
>HCN2	TTCAGGGGTGGAGCTACAATGGTGCAGTGGTAGAGCCAAGACAGCAGGGGTGGGGCTATCAGGTTGCTGAGCAGGGTCGAGGCAGCAGGGGCAGGGCTATGATGCTGCTGGGGTGGAGTCAAGCAGCAGGGGTGGGGCCGTGATATCTGGGTGGGGTCAAGGAGCAGGGGCAGGGCTGTGAGGGTGCTGGGCGGGGTCAAGGCATCAGGGGCGGGGCTATGAGGGTTGTGCTGAGCGGGGTCAGACATCCGAGTGGAGATATGAGGGTTGTGCTGGGCGGGGTCAGGCAGCAAGGGCGGGACTATGAGGGTTGTGCTGGGCGGGGTCAGGCAGCAGGGGCGGGGCAATGCGGGTTTTGCTGGGGCAGGGCCAGACATCAGGGGTGGGGATATGAGGGTTGTGCTGGGGCGGTGTCAGACATCAGGGGTGGGGAGCTGTGGATTTGGGGGGTGTCCTAGGGCGGGGGCTGTGGATTTGGGGGGGTCCTGCAGTGAAGGCTGTAGAGGTGGGCGGGGGTCCTGGGGTGGGGGCTGTGGGTTTGGAGGGCGGGGGGTCCCGGGGCAGGGCTGCAGGGTGGGGCGGGGGTCTTGTGCGGGGCCTTGGATTTGGGGGAGGGGGCCAGGAGCTCCCGCAGTGGGGGCGCACGGGGCTGGGGCTCTGAAGGTGGGGGCCGGGGGTCAGCGGGTAGGGTGGGCTC
>TPRX1	GGGCCTATAATTGGGCCTGGGCCTGAGATTGGGCCTGGGATTGGGCCTGGGATCAGGCCTGGGTTCGGGCCTGAGATTGGGCCTGGGATCGGGCCTGGGTTCGGGCCTGAGATTGGGCCTGGGATTGGGCCTGGGATCGGGCCTGGGTTTGGGCCTGAGATTGGGCCTGAGATTGGGCCTGGGATCGGGCCTGGGATCGGGACTGAGATTGGGCCTGGGATCGGNCCTGAGATTGGGCCTGAGATCGGGCCTGGGTTTGGGCCTGGGATCGGGCCTGGGTTTGGGCCTGAGAATGGGCCTGAGATTGGGCCTGGGATCGGGGCTGGGCCTGAAATTGGGCCTGGGATTGGGCCACGGAATGGGCCTGGGATCTGGACTGGGCCTGAAATTGGGCCTGGGATTGGGGCTGGGATCGGGCTTGGGATCTGAGCTGGGCCTGGAATTGGGCCTGGAATTGAGCCAGGGAGTGGGCCTGGGATCTGGGCTGGGCCTGGGATTGGGGCAGGGATCGGGCCAGGGCCTGGACTCAGGGCAGCTGGGATGCCCTTCTGGGCTCTGCACCCAGGGCA
>DIP2C	TGGGCGCAGGGAGGACAGGTGCGGGGAGCATGGACATGGGGAGCAGAGATGGGCGCGGGGAGCACGGGTGTGGGGAGCATGGGCGTGGGGAGAACGGGTGTGGGGAGCATGGGCGTGGGGA
>ADARB2	GGGGAGTAAACACCGGGAGGGAGGGGGGAGTAAACACCGGGAGACAGAGGGGAGTAAACACCGGGAGGCAGAGGGGAGTAAACACCGGGAGGCAGGGGGGAGTAAACACCGGGAGGGAGGGGGGA
>SARDHT	GGGTGAGGGAGAGGGAGGGTGAGGGGAGAGNGAGGGTGAGGGAGAGGGAGGGTGAGGGAGAGGGAGNGTGAGGGAGAGGGAGGGTGAGGGTGAGGGGAGAGGGAGGGCGAGGGGAGAGGGAGGGCGAGGGAGAGGGAGGG
>CELSR3	GGGANGGGTCATTTGTGATGAGGGAGAGGCTCTGACAAAAGTTAAGGTGTGGCAAGGTGGACAGTGGAGGGGAGATGTGGGAGGGAGGGTGGAGGGGANATGTGNGAGGNTGGAGGGGAGATGTNGNAGGGTNGAGGGTGGAGGGGAGATGTGNGAGGGTGGAGGGTGGAGGGGAGATGTGGGAGGGTCGAGGGTGGAGGGTGGAGGGGAGATGTGGGAGGGAGGGTGGAGGGTGGAGGGGAGATGTGAGAGGCAGGGTGGTGGGTGGAGGGGAGATGTGGGAGGGTGGAGGGTGGAAGGGAGATGTGGGAGGGAGGGTGGAAGGGAGATGTGGGAGGGAGGGTGGAGGGGAGATGTGGGAGGGTGGAGGGTGGAGGGGAGATGTGGGAGGGTGGAGGGTGGAGGGGAGATGTGNGANGGAGGGTGGAGGGGAGATGTGGGAGGGAGGGTGGAGGGGAGATGTGGGAGGGAGGGTGGAGGGGAGATGTGGGAGGGTGGAGGGTGGAGGGGAGATGTGGGAGGGAGGGTGGAGGGGAGATGTGGGAGGGAGGGTGGAGGGGAGATGTGGGAGGGTGGAGGGGAGATGTGGGAGGGAGGGTGGAGGGGAAATATGGGAGGGAGGGTGGAGGGGAGATGTGGGAGGGTCTGACACTGGGGAGGTCTAGAAAGGGGAAAATTGGCAGGAGGGCAACATAGTATCTGGTTGGGGGGACAGAGAGACAGGACTTGGCCCAGGGGTCCCCTAGGGGCTCTCACCTTCAGATGGGGATGGCCGTTTTAAACTGGGAAGGCAGCAGCACATGAAGGGC
>MYO7B	GCCCTTAGGGTGAATGGATGGGTGGGTGGATAGATGGGTGAGTGGGTGGATGGGTAGTGGGTGGGTGGATGGGTGAGTGGATGGATGGGTGGTGGGTGGGTGGATGGATGGGTGGGTGGATGGATGGGTGGGTGAGTGGATGGGTGGATGGGTGTGTGAGGGTGGATGGGTAAATGGATGTGTGGTTGGTTGGTTGGATGGATGGATGGATGAGTGGATGGATGGGTGAGTGGATGGACGGGTGAATGGGTGGTTGGGTGGATAGATGGGTGAGTGGGTGGATGGGTAGTGGATGGGTGGATGGATGGGTGAGTGAGTGGATGGGTGGTGGGTGGGTGGATGGATGGGTGGGTGAGTGGATGGGTGGATGGGTATGTAAGTGGATGGGTGAATGGGTGTGTCGATGGTTGGGTGGATGGGTGAGTGGGTGAATGGATAGATGGATGGGTGGGTGGCTGTGTGGATGGGTGAGTGGATGGACGGGTGGGTGGGTGGATGGGTGAATGGATGGGTGGATGGGTGAGTGGGTGGATGGATAGATGGACGGGCAGGTGGCTGTGTGGGTGGGTGAGTGGATGGATGGGTGGGTGGATGGGTGGGTGGGTGGATGGATAGATGGATGGGTGGGTGGCTGTGTGGATGGGTGAGTGGATGGATGGATGGGTGGGTGGAATGGTGAATGAATGGGTGCATGGGTGGGTGCATGTGGCAGCTTATATAAGGGGGTTTGGAGTGGGAATGGGAATCTGGGGGGTCCAGAGTTTAGATAGGAGAAGTGACTTTGTGCTGGGTAGATGGTGGGGGCCACGCCTGGGTAGAGGGGTGGAGAGCATGGCCTTAGTGGGGCCATGGGTGGATGGGTGAGTGGGTGGATGGGTGGTGAGTAGGTGGATGGATGGGTGAGTGGGTGGATGGGTGGTGCGTGGGTGGATGGATGAGTGGGTGCATGGGTGAATGGATGGGTGAATGGGTGGGTGGATGGGTGGGTGGGTGGATGGGTGAGAGGGTGGATGGATAGATGGATGGGCGGGTGGCTATGTGGGTTGGTGAGTGGATGGAAGGATGGATGGGTGGGTGGGTGGGTGAATGGATGGACGCATGGGTGGGTGGGTGGATGGGTGCTGGGTAGGTGGAAGGATGGGTGAGTGGGTGGATGGGTGGTGGGCGGGTGGATGGGTGGGTGGGTGGATGGATAGGTGTGTGAGTGGATGGGTGAAGGGGTGGGTGGATGGGTGAGTGGGTGGATGGATACATGGATGGGTGGGTGGGAGGGC
>PDE6B	GCGGGAATAGCATGGCATAGCGGGAATAGCATGGGGTAGGGGAATAGCGTGGGGTAGTGGGAGCAGCATGGGGTAGGGGAATAGCGTGGGGTAGTGGGAGCAGCATGGGGTAGGGGAATAGCGTGGGGTAGTGGGA
>TP73	GGGATTGCGGGAGGAGGGGCTGAGGCTGCAGGGCTGGGATTGCGGGAGGAGGGGCTGAGGCTGCATGGCTGGGATTGCGGGAGGAGGGGCTGAGGCTGCATGGCTGGGATTGCGGGAGGAGGGGCTGAGGCTGCAGGGCTS
>PSRX5	GCTGGGAGGGGGACGTTGGGAGGGTCCCTGGGGGCCCCGCTGTGCGTTCTCCTGTTCTGAGGCTGGGCGGGGGGCGGGGGACGTTGGGAGGGTCCCTGGGGGTCCCGCTGTGTGTTCTCCTGTTCTGAGGCTGGGCGGGGGGCGGGGGACGTTGGGAGGGTCCCTGGGGNCCNAACTGTGTGTTCTCCTGTTCTCAGGCTGGGAGGGGGACGTTGGGAGGGTCCCTGGGGGTCCCGCTGTGCGTTCTCCTGTTCTGAGGCTGGGAGGGGGACGTTGGGAGGGTCCCTGGGGGTCCCGCTGTGCGTTCTCCTGTTCTGAGGCTGGGAGGGGGACGTTGGGAGGGTCCCTGGGGGCCCCGCTGTGCGTTCTCCTGTTCTGAGGCTGGGAGGGGGACGGGGGACGTTGGGAGGGTCCCTGGGGGCCCAGCTGTGTGTTCTCCTGTTCTGAGGCTGGGCGGGGGGCGGGGGACGTTGGGAGGGTTCCCTGGGGGCCCAGCTGTGCGTTCTCCTGTTCTGAGGCAACCCT
>PRDM16	GCCCTCTGAGAAGGGTGTGCCGGAAGGGCATGCACCGGGAGGGGCATGTTAGGAGAGGGTGCACGGGGAGGGGATGTGTTGGGAGGGGGTGCGCCAGGAGGGGTGTGTTGGGAGGGCTGTGTTGGGATGAATGTGTTGGGAGGGGTGCATGGTGAGGGATTGCACTGGGAGGGGTGTGCTGGGAGGGCTGTGTTGGGATGAGTGTGTTGGGAGGGGTGTGTTGGGAGGGGTGTGTTGGGAGGGGTGTGCTGGGAGGGGTGTGCTGGGAGGGGTGTGTTGGGAGGGGTGTGCTGGGAGGGGTGTGTTGGGAGGGGTGTGCTGGGAGGGGTGTGCTGGGAGGGCTGTGTTGGGAGGGGTGTGTTGGGAGGGGTGTGCTGGGAGGGGTGTGCTGGGAGGGGTGTGTTGGGAGGGGTGTGTTGGGAGGGGTGTGCTGGGAGGACTGTGTTGGGATGGGTGTGTTGGGAGGGGTGTGCTGGGAGGACTGTGTTGGGATGGGTGTGCTGGGAGGGGTGTGCTGGGAGGGCTGTGTTGGGATGAGTGTGTTGGGAGGGGTGTGCTGGGAGGGGTGTGCTGGGAGGGGTGTGCTGGGAGGGGTGTGTTGGGAGGGGTGTGCTGGGAGGGGTGTGTTGGGAGGGGTGTGCTGGGAGGACTGTGTTGGGATGGGTGTGCTGGGAGGGGTGTGCTGGGAGGGCTGTGTTGGGATGAGTGTGTTGGGAGGGGTGTGTTGGGAGGGGTGTGCTGGGAGGGGTGTGTTGGGAGGGGTGTGCTGGGAGGGGTGTGCTGGGAGGGGTGTGTTGGGAGGGGTGTGCTGGGAGGGGTGTGTTGGGAGAGGTGTGCTGGGAGGGGTGTGCTGGGAGGGGTGTGTTGGGAGGGGTGTGCTGGGAGGGGTGTGTTGGGAGGGGTGTGTTGGGAGGGGTGTGCTGGGAGGACTGTGTTGGGATGAGTGTGTTGGGAGGGGTGTGCTGGGAGAGGTGTGCTGGGAGGGGTGTGTTGGGAGGGGTGTTTTGGGAGGGGTGTGCTGGGAGGGGTGTGCTGGGAGGGGTGTGTTGGGAGGGGTGAGTTGGGGGGGTGCACCGGGAGGGGTGCACCGGGAGGGGGTGTGTTGGGGAGGGTGCACCAGGAAGGGCGCGCTGGGAGGGTAAGGGC

Supplementary Table 6.1

	LG4	Flank Ctrl
**Chip seq loci**	**44**	**6**
**# total loci**	**151**	**123**
**# TF pulled down**	**80**	**7**
**# types**	**20**	**5**
CTCF	1	0
E2F4	3	1
E2F6	4	0
EBF1	3	0
EGR1	32	0
ELF1	3	0
FOSL1	2	1
FOSL2	4	0
FOXA1	1	0
HNF4a	1	0
JUND	3	2
MAX	1	0
NFR1	1	0
SP1	9	0
SRF	1	0
TR4	1	0
USF1	6	2
YY1	1	0
ZBTB33	2	1
SNF263	1	0

Supplementary Table 6.2

	# reg loci	# total loci
**LG4***	113	198
**Ctrl***	40	141
*p < 0.001		

Supplementary Table 6.3

	LG4*	Ctrl*
**Promoter**	25	4
**TFBS**	13	2
**CTCF**	28	14
**Promoter Flanking**	26	9
**Enhancer**	9	9
**OC**	32	6
*p < 0.03		

Supplementary Table 6.4

	LG4	CTRL
**Chip seq loci***	**44**	**6**
**# total loci***	**151**	**123**
**# TF pulled down**	**80**	**7**
**# types**	**26**	**5**
E2F6	4	0
EGR1	32	0
FOSL2	4	0
SP1	9	0
USF1	6	2
*p < 0.0001		

Supplementary Table 7. Overlapping LG4/GeneHancer Enhancers. The 180 LG4s found to overlap with annotated GeneHancer Enhancers and corresponding enhancers are listed. Full GeneHancer entries, chromosomal positions for LG4s and enhancers, and length of overlap are indicated.

INCLUDED AS SEPARATE EXCEL SPREADSHEET

Supplementary Table 8. Lys2 reversion rates and sequencing data.

Strain #	frameshift	promoter	genotype	rate x10-8	CI low	CI high	% del	% dupe	# Del	# dupe	# Total seq
**G4 LYS+1**											
4547	lys +1	pLys	G4 WT	5.37	4.4	6.68	0.83	0	53	0	64
4552			G4 TS	3.3	2.76	4.17	0.78	0	28	0	36
4713			G4 rad51	17.8	13.5	26.9	0.83	0	44	0	53
4741			G4 lig4	5.6	4.93	6.89	0.92	0	48	0	52
4921			G4 pif1Δ rrm3Δ	32.8	25.1	50.8	0.98	0	47	0	48
4977		GAL-LYS	G4	27.6	24.4	28.9	0.6	0	25	0	42
**GCA LYS+1**											
4709	lys+1	pLys	GCA	3.67	2.65	4.25	0.75	0.02	40	1	53
4808			GCA rad51	12.5	9.7	17.8	0.73	0	36	0	49
4947			GCA pif1Δ rrm3Δ	31.3	16.5	42.2	0.98	0	51	0	52
1979		GAL-LYS	GCA	19.3	15.3	23.1	0.66	0	29	0	44
**G4 LYS-1**											
4548	lys-1	pLys	G4 WT	3.29	2.91	3.66	0	0.64	0	37	58
4553			G4 TS	2.07	1.66	2.47	0.04	0.67	1	18	27
4714			G4 rad51	3.36	2.69	3.36	0.02	0.56	1	29	52
4742			G4 lig4	2.66	1.93	3.68	0.02	0.85	1	45	53
4922			G4 pif1Δ rrm3Δ	21.2	13.2	25.7	0.05	0.78	3	43	55
4978		GAL-LYS	G4	28.3	25.2	30.6	0	0.84	0	38	45
**GCA LYS-1**											
4710	lys-1	pLys	GCA	2.86	2.08	3.24	0.04	0.73	2	41	56
4948			GCA pif1Δ rrm3Δ	11.5	6.72	17.9	0.04	0.85	2	47	55
4980		GAL-LYS	GCA	19.9	17.4	23.8	0	0.57	0	25	44

Supplementary Table 9. Effects of altering LG4ID parameters.

Window size (bp)	GGG count / 100bp	Chr21 LG4 Calls
1500	8	5
1500	12	0
1500	4	618
500	8	116
500	12	9
500	4	3616
250	8	389
250	12	46
250	4	10030

Supplementary Table 10. LG4 Hi-C interactions. List of chromosomal interactions between LG4 loci (and control sequences) and other genomic locations. Interaction data was obtained from UCSC Genome Browser using Hi-C and Micro-C track. Columns are as follow: Chrom=Chromosome (or contig, scaffold, etc.). For interchromosomal, use 2 records; chromStart=Start position of lower region. For chromosomal, set to chromStart of this region; chromEnd=End position of upper region. For interchromsomal, set to chromEnd of this region; name=Name or ID of item. Usually name1/name2 or name1/name2/exp or empty; score=Score from 0-1000, typically derived from value; value=Strength of interaction or other data value; exp=Experiment name for filtering. Use . if not applicable; color=Item color, as itemRgb in bed9. Typically based on value or exp; sourceChrom=Chromosome of source region (directional) or lower region. For non-directional interchromosomal, chrom of this region; sourceStart=Start position of source/lower/this region; sourceEnd=End position of source/lower/this region; sourceName=Identifier of source/lower/this region; sourceStrand=Orientation of source/lower/this region: + or -. Use . if not applicable; targetChrom=Chromosome of target region (directional) or upper region. For non-directional interchromosomal, chrom of other region; targetStart=Start position of target/upper/this region; targetEnd=End position of target/upper/this region; targetName=Identifier of target/upper/this region; targetStrand=Orientation of target/upper/this region: + or -. Use . if not applicable.

INCLUDED AS SEPARATE EXCEL SPREADSHEET

Supplementary Information 1. LG4ID graphical algorithm workflow, summary, and training.

LG4ID Summary:

LG4 (inputString, windowSize=1500, mustExceed=120)

LG4ID finds all non-overlapping "GGG" and "CCC" in inputString, and records the index of each "GGG" and "CCC" into "GGG Boolean List" and "CCC Boolean List", respectively. To clarify "non-overlapping", "GGGGGG" is two "GGG", not four "GGG". "GGG Boolean List" and "CCC Boolean List" are both of length(inputString) - 2, and used to reference all "GGG" and "CCC" in the "inputString". Such Boolean lists are used to obtain linear search-time complexity.

Next, LG4ID iterates through "inputString“ (Boolean Lists in this case) in "windowSize" overlapping bins, advancing by one index at a time. This guarantees that all LG4s will be found, as opposed to non-overlapping bin approaches. For each bin LG4ID counts “GGG”s and “CCC”s separately and obtains the maximum value among those, which is called the "maximum Hits". The "maximum Hits" is calculated by only one strand and is thus a pessimistic measure. If "maximum Hits" exceeds "mustExceed“ (‘120’) then the bin is a valid LG4, otherwise it is not. All valid LG4 bins which overlap or neighbor by start and stop coordinates are joined into contiguous LG4s, which are returned to the user.

LG4ID Workflow:

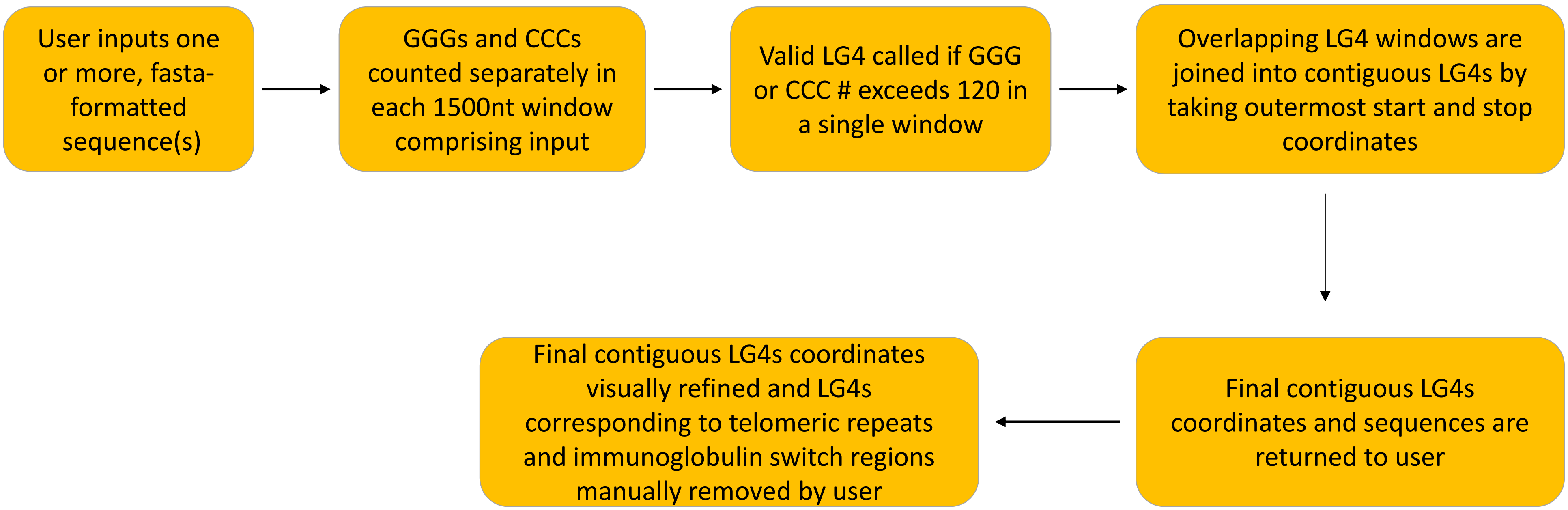

LG4ID Training: 120 GGG (or CCC) / 1,500 bp was based on:

Human IgHμ Switch region   hg38:Chr14:105857086-105858663   123 CCC in 1,489 bp

CCCAGCCCAGCTAAGCCCAGTATAGCCCAGCCCAGCTAAGCCCAGTATAGCCCAGCCCAGCTAAGCCCAGTATAGC
CCAGCCCAGCTAAGCCCAGTTTAGCCCAGCCCAGCTAAGCCCAGTATAGCCCAGCCCAGCTAAGCATAGTTCAGCA
CAGCCCAGCTCAGCTCAGCACAGTTCAACCCAGCTCAGCCCAGCTCAGTGCAGCACAGCCCAGCTTAGCCCAGCCC
AGCCCAGCTCAATCCAGCCTGGCTCAGCCCAGCCCAGCCCAGTTTGGCTCAACCCAGCTTGGCTCAGCCCAGGTCA
GCCTGGCTCAACTCAGCCCAGCCCAGCCCAGCTCTGCTCAACCCAGCTCTGCTCAACTCAGCCCGGCTCAGCCCAG
CTCAGCCCAGTTCAGCTCAGCCCTGCTCAGCACAGCACAGCAGAGCTCAGCTCAGCCCAGCTCAGCTCAGTTCAGC
TCAGCCCTGTTCAGCACAGCACAGCAGAGCCCAGCCCAGCCCAGCCCAGCTCATCCCAGCTCAGCCCAGCCCAGCC
TAGCTTAGCTCAACCCAGCTCAGCACAGTTCAGCTCAGCCCTGCTCAGCACAGCACAGCAGAGCCCAGCTCAGCCC
AGCTCAGCTCAGTTCAGCTCAGCCCTGTTCAGCACAGCACAGCAGAGCCCAGCCCAGCCCAGCTCAACCTAGCCTG
GCTCAGCCCAGCCCAGCCCAGCCCGGATCGGCTCAACCCAGCTTAGCTCAGCCCAGGTCAGCCCAGCTTAACTCAG
CCCAGGTCAGCCCAGCTTAACTCAGCCCAGCCCAGCCCAGCTCAGCCCCCAGCTCTGCTCAACCCAGCCCAGCTCA
GCTCAGCTCCGCTCAGCCCAGTTCAGCCCAGCTCAGCCCAGCCCAGCCTAGCTTGGCTCAACACAGCTCAGCTCAG
CCAGCCCAGACCAGCTCAGCTCAGCCCAGTCCAGCTCAGCTCAGCCCAACCCAGTCCGGCTCAGCCCAGCCCAACC
CAGCCCAGCCCAACCCAGCTCGGCTTAACCCAGCTCGGCTCAGCCCAGATCAGTCTGGCTCAACTCAGCCCAGCCC
AGCTCAACCCAGCCCAGTTCAGCCCAGCTCATCCAAGCTCAGCTCAGCCCAGCCCAGTTCAGCCCAGCCCAGTTCA
GCTCAGCTCAGCCCAGCCCAGTTCAGCCCAGCCCAGTTCAGCTCAGCTCAGCTGAGCCCAGCCCAGCCCAGTCCGG
CTCAGCTCAGCCCCGCCCCACTCAGCCCAGCTCAGCTCAGCCCAGCTCAGCCCAGCTCAGCTTAGCCCAGCTCAGTT
CAGCCAGGCACAATCTGGCTCAGCCCAGCCCAGCCCAGCTCAGCCCAGCCCAGCCTAGCTCAGCACAGCCCAGTTC
AGCTCAGCTCAGCTTAACTCAGCTCAGCTCAGCTCAGCCCAGCCCAGCCCAGGTCAGCTCAGCCCAGCCCAGCCCA
GCCCAGATCATCCCAGCTCAGCTCAGCTCAGCTCGGCTTAGCCC

Supplementary Information 2. Occurrence of annotated structural variations at LG4 loci. Ten additional LG4 loci were selected then visualized in the UCSC browser after being zoomed out 10x making the central 10% of each window correspond to reported LG4 positions. The "database of structural variants" track listing (CNVs, Inversions, Insertions and Deletions) was selected.

Casper J, et al. The UCSC Genome Browser database: 2018 update. Nucleic Acids Res. 2018 Jan 4;46(D1):D762-D769. doi: 10.1093/nar/gkx1020.

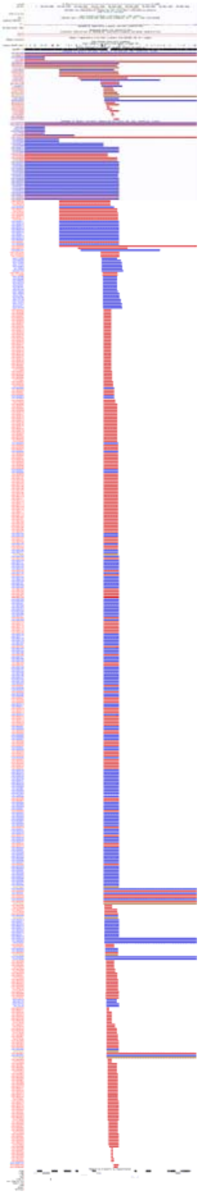

16:88546871-88547886

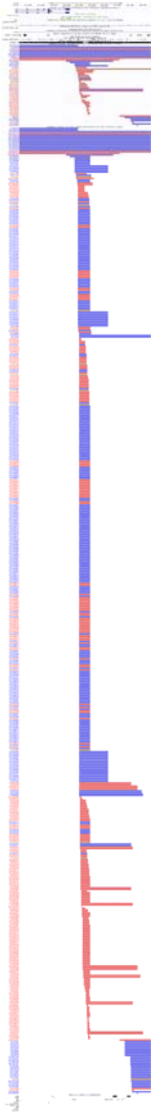

19:376684-377716

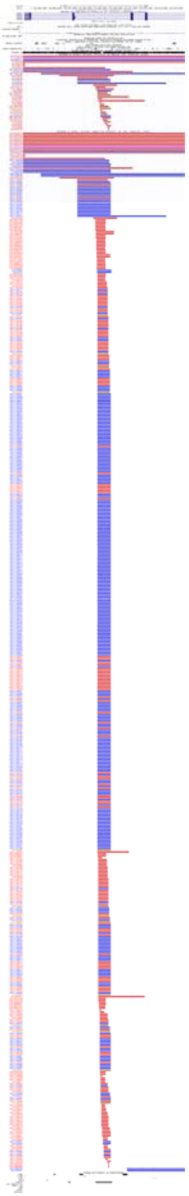

9:133668283-133669352

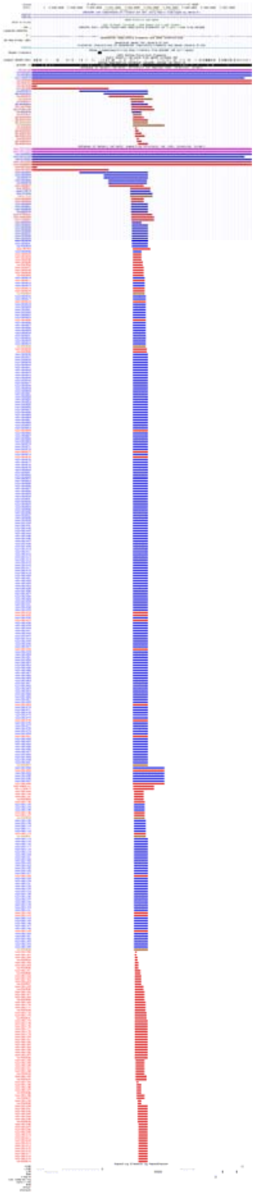

4:7441549-7442705

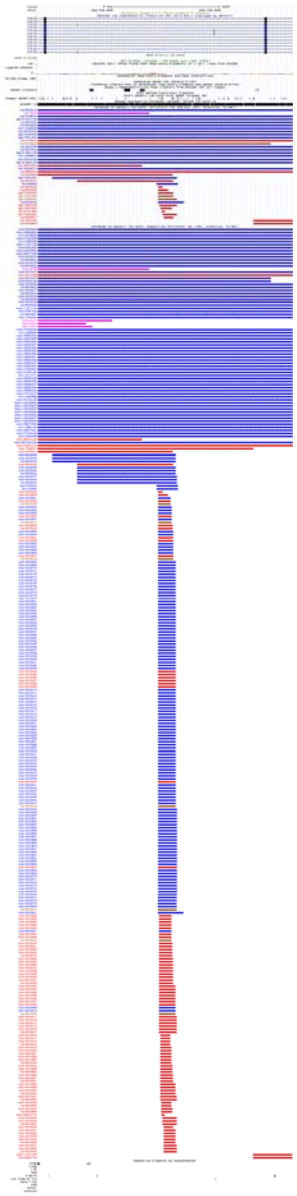

2:240731865-240733047

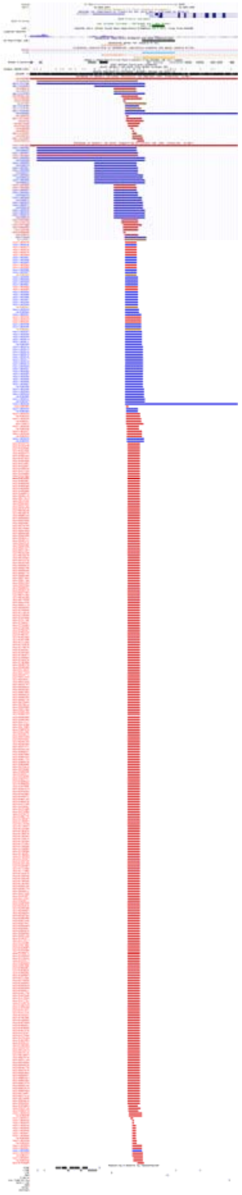

7:44960832-44962055

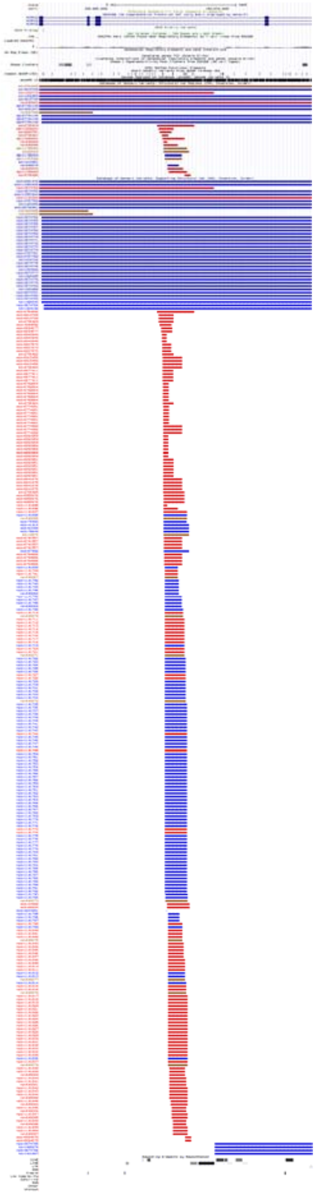

7:158667266-158668388

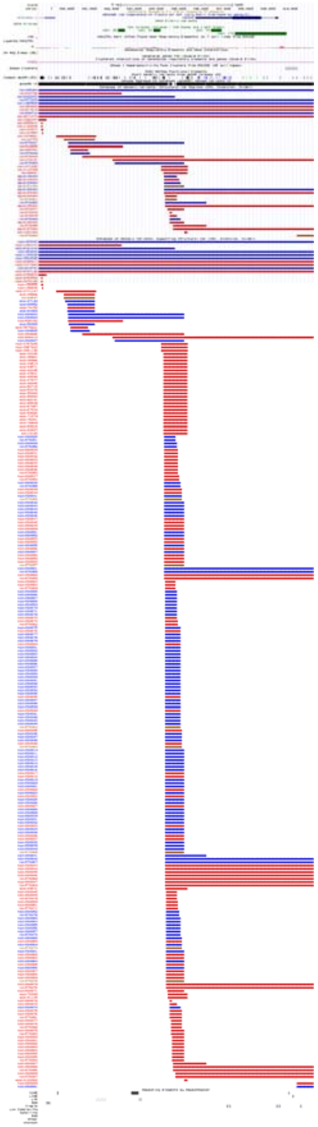

19:403958-405188

12:123911267-123912498

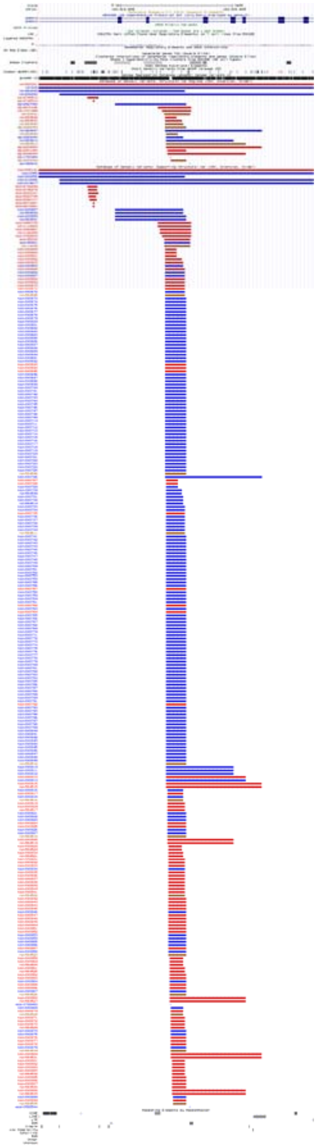

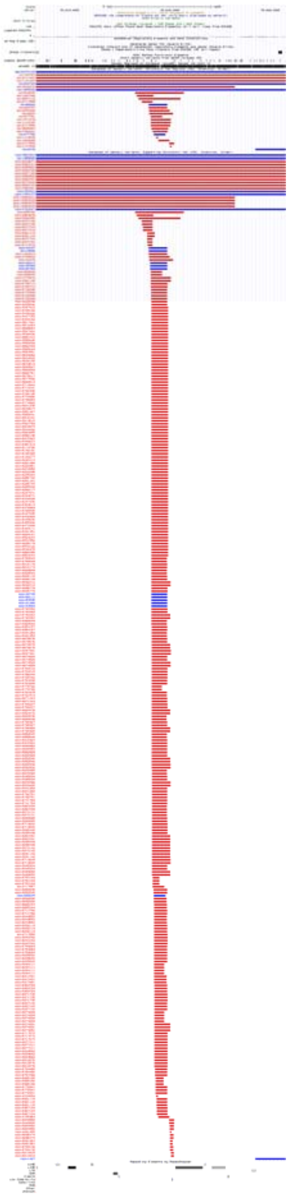

18:78613479-78614723

Supplementary Information 3.

Detailed analysis of the role of G4 in *DIP2C* intronic LG4 mutation.

G4-forming potential is not the primary driver of large deletions/duplications in *DIP2C*

Of note, the orientation of a G-rich sequence on the non-transcribed strand (NTS) verses transcribed strand (TS) can increase G4 formation from the presence of R-loops(Kim and Jinks-Robertson, 2012). R-loops are TS-DNA-RNA hybrids that prevent the NTS from re-annealing, and the persistent single stranded state increases the ability for G4 structure formation. As such, in order to further examine the effects of G4 presence on deletions / duplications, *DIP2C* was inserted into *LYS2* in both orientations (NTS or TS) and frameshifts (+1 or -1). We found both deletions (95% CI) and duplications (Mann-Whitney) were significantly enriched when the G-rich sequence was on the NTS versus TS (**Figure SI3F1A**). This indicates that increasing structure formation through transcript orientation can increase deletion and duplication events, and therefore, that the formation of G4 can influence mutagenesis. That said, the frequent occurrence of large deletions/duplications in the *DIP2C* sequence could reflect the propensity of this sequence to form stable G4 structures that interfere with replication or could simply reflect the repetitive nature of the sequence. To distinguish between these possibilities, we constructed a non-G4 forming control sequence by strategically disrupting all 3-4N guanine runs with either cytosine or adenine (GCA control; **Figure SI3F2A**). Such changes would be expected to maintain homology at the deletion and duplication endpoints observed in the *DIP2C* sequence while limiting potential hairpin formation (**Figure SI3F2B**). Only the endpoint homology of event J was reduced in the GCA control sequence (from 4 bp to 2 bp). CD analysis of the GCA control sequence confirmed its inability to adopt a G4 structure *in vitro* (**Figure SI3F2C**). The rate of deletions in the GCA control sequence was reduced ~40% relative to *DIP2C* ($p<.001$), but the rate of duplications was not affected (**Figure SI3F1B**). These data suggest that the repetitive nature of *DIP2C* is the primary cause for large deletions / duplications in the frameshift-

reversion assay, with the potential for G4 formation having little, if any, effect in an otherwise wild-type (WT) background.

Pif1 and Rrm3 are helicases implicated in the removal of G4 structures in yeast and each has been shown to suppress gross chromosomal rearrangements that map to G4 loci(Ribeyre et al., 2009)(Paeschke et al., 2013). We thus examined the effects of deleting *PIF1* and/or *RRM3* on reversion of *lys2* frameshift alleles containing the *DIP2C* or GCA control sequence. While individual removal of these helicases had no effect on reversion rates of the *lys2::DIP2C* or *lys2::GCA* alleles (data not shown), simultaneous removal of both helicases (*pif1Δ rrm3Δ* double mutant) increased deletions and duplications associated with each ~7-fold (**Figure SI3F1C**). Removal of Pif1 leads to a petite phenotype. However, deletion of Abf2 as a control for the petite phenotype had no effect on the mutation rate of either *lys2::DIP2C* frameshift (data not shown). The lack of a differential effect of Pif1 and Rrm3 on instability of the *DIP2C* versus the GCA control sequence again suggests that the G4-forming potential of DIP2C is not the primary driver of large deletions/duplications.

The location of a G-rich sequence with G4-forming potential on the non-transcribed strand (NTS) versus the transcribed strand (TS) of a gene has been associated with increased instability(Kim and Jinks-Robertson, 2012)(Williams et al., 2015). This has been attributed to persistent base pairing between the TS and the transcript (R-loop formation) which leaves the NTS in a single-stranded state that promotes G4-structure formation. In the constructs described above, the *DIP2C* sequence was inserted into *LYS2* so that the G-rich strand was the NTS. To examine a possible transcriptional effect on *DIP2C*-associated instability, we inserted the sequence in the reverse orientation so that the TS was the G-rich strand. Both deletions and duplications were significantly reduced when the G-rich sequence was moved from the NTS to the TS ($p < 0.001$ and $p < 0.01$ by Mann-Whitney U-test, respectively; **Figure SI3F1A**) suggesting that transcription-associated G4 formation is not solely responsible for deletions/duplications.

Previous studies have reported increased mitotic loss-of-heterozygosity and gross chromosomal rearrangements at G4-forming sequences under high-transcription conditions(Yadav et al., 2014)(Williams et al., 2015). We thus examined the effect of highly elevated transcription levels on reversion of the *lys2::DIP2C* and *lys2::GCA* alleles by replacing the endogenous *LYS2* promoter with the *GAL1* promoter. We found the higher transcriptional activity associated with the *GAL1* promoter stimulated deletions / duplications in both types of *lys2* alleles (**Figure SI3F1D**), and that this was enhanced when the *DIP2C* sequence was also present. Notably, large deletions were 1.3-fold higher in the *lys2::DIP2C* than the *lys2::GCA* allele, and large duplications were elevated 2-fold. Although the transcription-associated effects on the *lys2::DIP2C* relative to *lys2::GCA* alleles are somewhat subtle, they nevertheless suggest that G4 formation may contribute to the formation of large deletions / duplications within repetitive sequences.

Non-homologous end joining and homologous recombination are not responsible for deletions and duplications.

Another mechanism underlying formation of deletions or duplications is double stranded breaks repair. The predominant double strand break repair pathways are non-homologous end joining (NHEJ) and homologous recombination (HR). Classical-NHEJ is the direct ligation between two broken strands where the *Ku* heterodimer binds the broken ends and acts as a docking site for downstream proteins involved in the repair process, such as *Ligase IV* (*Lig4*)(Daley et al., 2005). Both *Ku* and *Lig4* are required for classical-NHEJ repair. There is also alternative-NHEJ repair, or microhomology-mediated end joining (MMEJ), that is independent of *Ku*, dependent on microhomology, and partially dependent on *Lig4*(Ma et al., 2003). Since deletions and duplications all contained microhomology at their boundaries, removal of *Lig4* could indicate if MMEJ, or classical-NHEJ were involved. However, we find removal of *Lig4* has

no effect on either deletions or duplications implying neither MMEJ nor classical NHEJ are involved in their formation (**Figure SI3F1E**).

HR repair occurs between two highly homologous sequences and involves end processing followed by strand invasion of the broken end onto the repair template(Symington et al., 2014). After strand invasion, there are two predominant HR repair mechanisms, the double-strand break repair and synthesis-dependent strand-annealing pathways. *Rad51* is integral for the strand invasion step of HR and, as such, was deleted to allow us to determine if either HR downstream pathway is involved in *DIP2C* G4 deletion or duplication. In this haploid yeast system, *DIP2C* sequences were only present in our reporter gene, and the sister chromatid provided the substrate for HR repair during replication. That said, we found *Rad51* removal did not significantly alter the observed duplication rate (green bars **Figure SI3F1F**). In contrast, deletions increased 3 fold without *Rad51* indicating that HR can prevent loss of the repeat (red bars **Figure SI3F1F**). Nevertheless, the deletions in the GCA control increased at a similar rate compared to *Rad51* proficient yeast indicating that G4 structures are not responsible for the increase (**Figure SI3F1F**). By ruling out the two major double strand break repair pathways, we favor a mechanism by which replication stress and slippage is responsible for deletions and duplications. Further, at times of low level expression sequence repetitiveness seems to be the main driving force behind mutagenesis in *DIP2C*-G4, whereas half of all duplication events can be attributed to G4 structure formation during periods of high transcriptional activity.

Supplementary Information Discussion

As the mechanism(s) of increased SNPs and indels at LG4s cannot be deciphered from computational data, and were not found to be elevated in our *DIP2C* yeast studies, it is possible that observed increases are due to elevated nucleotide damage brought on by a more frequent single stranded state, error prone DNA synthesis, or inhibition of proper nucleotide repair from G4 structures. All are viable options considering: (1) LG4s are enriched in open chromatin, (2)

G4 structures can inhibit base excision repair(Broxson et al., 2014)(Holton and Larson, 2016), (3) translesion polymerases are needed for replication to bypass G4 structures(Sarkies et al., 2010), and (4) induce small mutations at G4 to prevent large chromosomal rearrangements (Koole et al., 2014). Another possibility is the increased repair of double strand breaks at LG4s. G4 loci are highly enriched at translocation hotspots and one prominent hypothesis is the aberrant resolution of DNA breaks is responsible in initiating mutagenesis(Kasparek and Humphrey, 2011). Most DNA breaks are repaired in an accurate manner, but can occasionally lead to small insertions and deletions(Nambiar et al., 2011)(Kane et al., 2012). Therefore, the increase of double strand breaks at LG4s could be responsible for the increase of indels. One of the most unexpected findings arising from this work was that repeat expansions and contractions are at times predominantly driven by the repetitiveness of the sequence and not on the ability to form G4 structures. Since HR and NHEJ were not involved in formation of deletions or duplications (**Main Text Figure 5, Supplementary Information 3 Figure 1**), we favor a hypothesis where replication stress through highly repetitive sequences induces slippage, or replication restart, leading to mutagenesis. Further evidence was provided for replication stress by the removal of helicase activity which increased deletion and duplication rates 7-fold in a non-G4 dependent manner. While G4 helicase removal had no G4-specific effect, there was an orientation specific increase that stabilized G4 structures. High levels of transcription led to a 10-fold increase over endogenous *LYS2* transcription, and a 2-fold increase in G4 associated duplications (**Main Text Figure 5, Supplementary Information 3 Figure 1**). This suggests that high transcriptional activity can lead to formation of R-loops that in-turn stabilize G4 structures. This could conceivably further increase replicative stress and exacerbate duplication frequency in a G4 specific manner.

While we elected to employ the yeast *LYS2* frameshift reversion assay because it has the capability to capture both small and large deletions and duplications (both found enriched in LG4 genome variation analysis), the *LYS2* frameshift reversion assay does have its drawbacks

in that net deletions and duplications of 3 bp will not be detected. Having registered this, since large repeat deletions and duplications seem to be the predominant mutation associated with LG4s, future studies should include a *URA3* intron reversion or inactivation assay(Shishkin et al., 2009)(Cherng et al., 2011)(Aksenova et al., 2015). By placing a >1 kb repeat in the *URA3* intron, any repeat contraction below ~1 kb can be detected by selection on media lacking uracil. Alternatively, placement of a small repeat allows the detection of any repeat expansion past ~1 kb resulting in URA3 inactivation and growth on media with 5FOA. Besides repeat contractions, selection on 5FOA would also be able to detect large genomic rearrangements at *URA3*(Aksenova et al., 2013)(Chen and Kolodner, 1999), as well as any increase in SNPs and indels in the surrounding *URA3* gene(Tang et al., 2013).

Finally, the discrepancy of G4 involvement in mutagenesis between high transcription and helicase removal is clearly perplexing. Since sequence repetitiveness, and not G4 formation, is the driving force in *DIP2C* mutagenesis, it is possible the large increase from helicase stress drowns out any G4 specific effects observed in high transcription duplications. Alternatively, previous studies of *Pif1* in yeast have shown that *Pif1* helicase only suppresses damage at short looped G4 structures(Piazza et al., 2015). A computational model(Kikin et al., 2006) of potential *DIP2C* G4 structures contains loops of 5 and possibly 9 bp long, both not shown to be destabilized in the *Pif1* mutant background. While the author's interpretation was that longer looped G4 structures do not form *in vivo*, G4 specific antibody ChIP detected a high prevalence of larger looped G4 structures in the human genome(Hänsel-Hertsch et al., 2017). Therefore, it is feasible that larger looped structures form *in vivo*, but are not substrates for *Pif1*, and these structures can lead to instability in other backgrounds that increase G4 formation such as high transcription. Another possibility is that G4 rarely forms during endogenous transcription, making removal of G4 helicases a non-factor under this condition. In the future, further removal of helicases under high transcription should be done to determine if loss of helicases exacerbate instability when mutations can already be attributed to G4 structures.

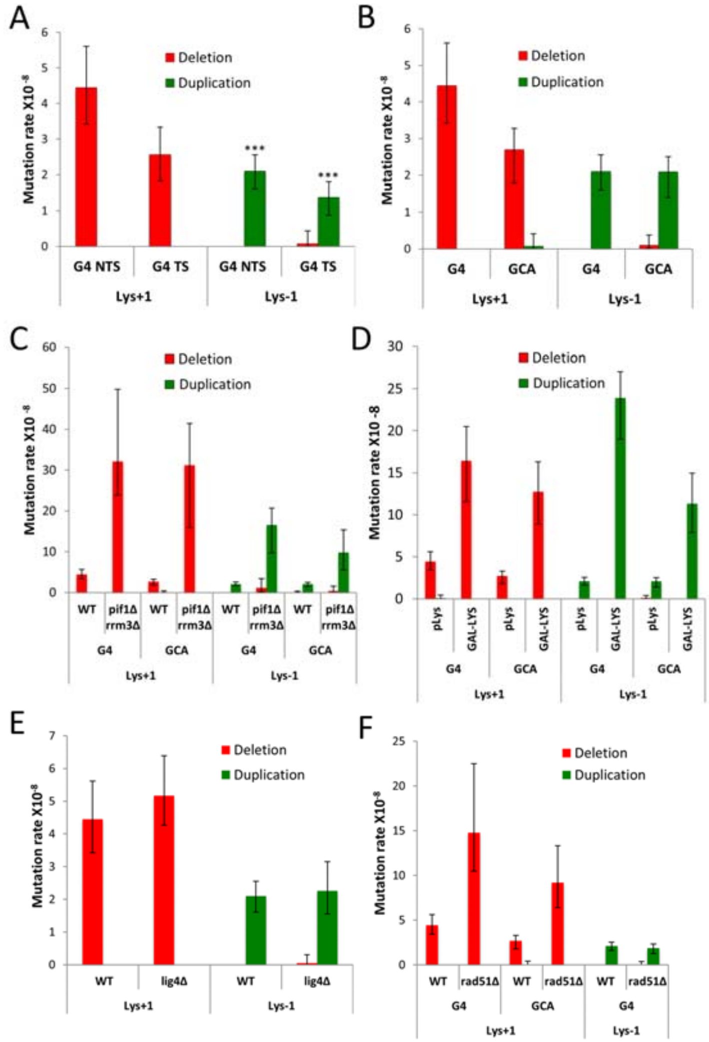

Supplementary Information 3 Figure 1. G4 structure formation is not the predominant driver of *DIP2C* deletions and duplications. The mutation spectrum generated from *LYS2* reversion window sequencing was used to calculate the deletion and duplication mutation rate (x10^{-8}) (y-axis) from the total *LYS2* reversion rate for both the +1 (Lys+1) and -1(Lys-1) frameshift. Error bars were calculated from 95% confidence intervals and adjusted for proportion of a given mutation. **(A.)** The G-rich sequence of *DIP2C* LG4 was placed on the non-transcribed strand of *LYS2* (G4 NTS) or the transcribed strand (G4 TS). *** Denotes where 95% confidence intervals overlap but rates are still significant according to Mann-Whitney ($p<.001$). **(B.)** GCA indicates a control sequence unable to form G4, but is highly repetitive and similar to *DIP2C*'s G4 sequence (G4) (x-axis). **(C.)** The Deletion and duplication rate (y-axis) of G4 helicases *pif1*Δ and *rrm3*Δ yeast alongside proficient helicase strains (WT)(x-axis). **(D.)** The deletion and duplication rate (y-axis) of endogenous *LYS2* transcription (pLys) and high *LYS2* transcription driven by the GAL1 promoter (GAL-Lys) (x-axis). Non-homologous end joining and Homologous recombination are not responsible for deletions and duplications. The deletion and duplication mutation rate (x10^{-8}) (y-axis) for both the +1 (Lys+1) and -1(Lys-1) frameshift is graphed with error bars calculated from 95% confidence intervals and adjusted for proportion of a given mutation. **(E.)** The deletion and duplication rates (y-axis) of NHEJ deficient (*lig4*Δ) and proficient (WT) yeast (x-axis) with *DIP2C*-G4 inserted into *LYS2*. **(F.)** The deletion and duplication rate for HR deficient (*rad51*Δ) and (WT) yeast in *DIP2C* LG4 (G4) or non-G4 control (GCA) (x-axis).

A

DIP2C sequence cloned into yeast *lys2*

GCCCTTTGGGCGCAGGGAGGACAGGTGCGGGGAGCATGGACATGGGGAGCAGAGATGGGCGCGGGGAGCACGGGTGTGGGGAGCATGGGCGTGGGGAGAACGGGTGTGGGCGTGGGGAAAGGG
C

GCA control sequence cloned into yeast *lys2*

GCCCTTTGGACGCACGGAGGACAGGTGCGCCGAGCATGGACATGCCGAGCAGAGATGGACGCGCCGAGCACGGATGTGCCGAGCATGGACGTGCCGAGAACGGATGTGGACGTGCCGAAAGGA
C

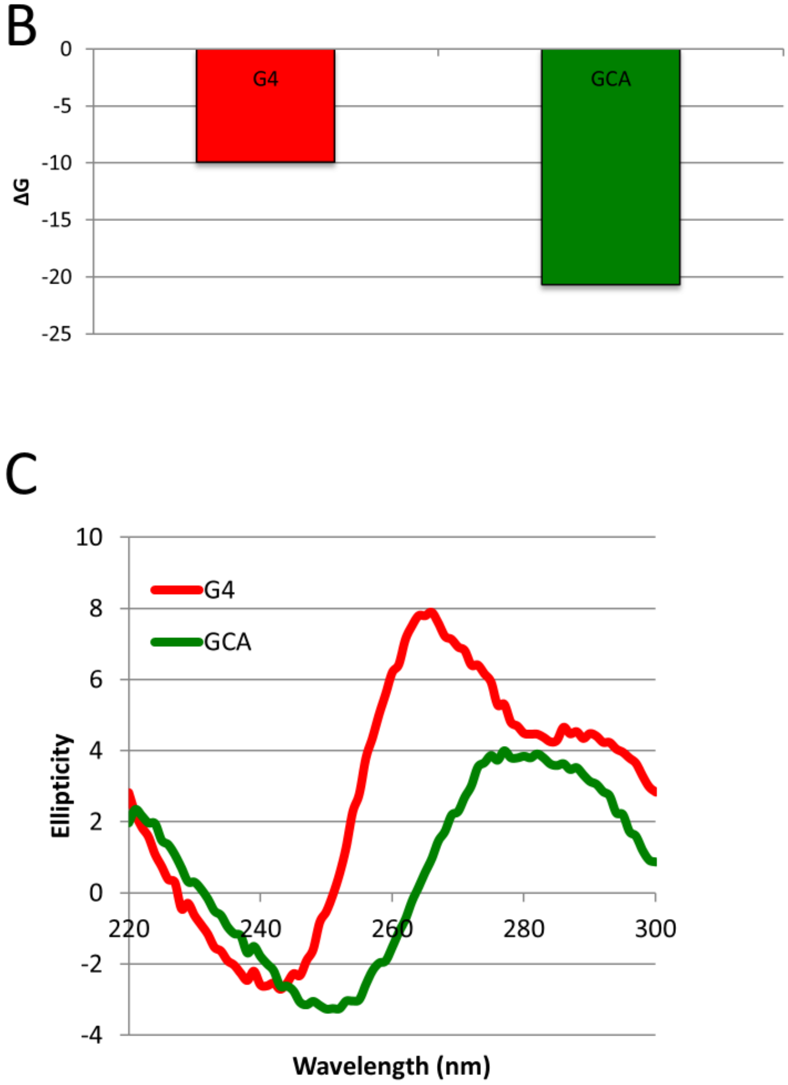

Supplementary Information 3 Figure 2. Construction of non-G4 forming repetitive control GCA. **(A.)** The sequences for *DIP2C* LG4 (top) and GCA control (bottom) are shown with G-repeats in red text and cytosine or adenine interrupted repeats shown in green. **(B.)** M-fold sequence analysis of *DIP2C*'s G4 and GCA control's (x-axis) ability to form hairpins (ΔG y-axis) (top) and subsequent predicted structures (bottom). **(C.)** Circular dichroism ellipticities of oligonucleotides representing *DIP2C* LG4 (red line) or GCA control (green line) (Ellipticity) (y-axis) is displayed from a 220-300 wavelength (nm).

References

Aksenova, A.Y., Greenwell, P.W., Dominska, M., Shishkin, A.A., Kim, J.C., Petes, T.D., and Mirkin, S.M. (2013). Genome rearrangements caused by interstitial telomeric sequences in yeast. Proc. Natl. Acad. Sci. U. S. A. *110*, 19866–19871.

Aksenova, A.Y., Han, G., Shishkin, A.A., Volkov, K. V., and Mirkin, S.M. (2015). Expansion of Interstitial Telomeric Sequences in Yeast. Cell Rep. *13*, 1545–1551.

Broxson, C., Hayner, J.N., Beckett, J., Bloom, L.B., and Tornaletti, S. (2014). Human AP endonuclease inefficiently removes abasic sites within G4 structures compared to duplex DNA. Nucleic Acids Res. *42*, 7708–7719.

Chen, C., and Kolodner, R.D. (1999). Gross chromosomal rearrangements in Saccharomyces cerevisiae replication and recombination defective mutants. Nat. Genet. *23*, 81–85.

Cherng, N., Shishkin, A.A., Schlager, L.I., Tuck, R.H., Sloan, L., Matera, R., Sarkar, P.S., Ashizawa, T., Freudenreich, C.H., and Mirkin, S.M. (2011). Expansions, contractions, and fragility of the spinocerebellar ataxia type 10 pentanucleotide repeat in yeast. Proc. Natl. Acad. Sci. U. S. A. *108*, 2843–2848.

Daley, J.M., Palmbos, P.L., Wu, D., and Wilson, T.E. (2005). Nonhomologous end joining in yeast. Annu. Rev. Genet. *39*, 431–451.

Hänsel-Hertsch, R., Di Antonio, M., and Balasubramanian, S. (2017). DNA G-quadruplexes in the human genome: detection, functions and therapeutic potential. Nat. Rev. Mol. Cell Biol. *18*, 279–284.

Holton, N.W., and Larson, E.D. (2016). G-quadruplex DNA structures can interfere with uracil glycosylase activity in vitro. Mutagenesis *31*, 385–392.

Kane, D.P., Shusterman, M., Rong, Y., and McVey, M. (2012). Competition between replicative and translesion polymerases during homologous recombination repair in Drosophila. PLoS Genet. *8*, e1002659.

Kasparek, T.R., and Humphrey, T.C. (2011). DNA double-strand break repair pathways, chromosomal rearrangements and cancer. Semin. Cell Dev. Biol. *22*, 886–897.

Kikin, O., D'Antonio, L., and Bagga, P.S. (2006). QGRS Mapper: a web-based server for predicting G-quadruplexes in nucleotide sequences. Nucleic Acids Res. *34*, W676-82.

Kim, N., and Jinks-Robertson, S. (2012). Transcription as a source of genome instability. Nat. Rev. Genet. *13*, 204–214.

Koole, W., Van Schendel, R., Karambelas, A.E., Van Heteren, J.T., Okihara, K.L., and Tijsterman, M. (2014). A polymerase theta-dependent repair pathway suppresses extensive genomic instability at endogenous G4 DNA sites. Nat. Commun. *5*.

Ma, J.-L., Kim, E.M., Haber, J.E., and Lee, S.E. (2003). Yeast Mre11 and Rad1 proteins define a Ku-independent mechanism to repair double-strand breaks lacking overlapping end sequences. Mol. Cell. Biol. *23*, 8820–8828.

Nambiar, M., Goldsmith, G., Moorthy, B.T., Lieber, M.R., Joshi, M. V., Choudhary, B., Hosur, R. V., and Raghavan, S.C. (2011). Formation of a G-quadruplex at the BCL2 major breakpoint region of the t(14;18) translocation in follicular lymphoma. Nucleic Acids Res. *39*, 936–948.

Paeschke, K., Bochman, M.L., Daniela Garcia, P., Cejka, P., Friedman, K.L., Kowalczykowski, S.C., and Zakian, V.A. (2013). Pif1 family helicases suppress genome instability at G-quadruplex motifs. Nature *497*, 458–462.

Piazza, A., Adrian, M., Samazan, F., Heddi, B., Hamon, F., Serero, A., Lopes, J., Teulade□Fichou, M., Phan, A.T., and Nicolas, A. (2015). Short loop length and high thermal stability determine genomic instability induced by G□quadruplex□forming minisatellites. EMBO J. *34*, 1718–1734.

Ribeyre, C., Lopes, J., Boulé, J.-B., Piazza, A., Guédin, A., Zakian, V.A., Mergny, J.-L., and Nicolas, A. (2009). The yeast Pif1 helicase prevents genomic instability caused by G-quadruplex-forming CEB1 sequences in vivo. PLoS Genet. *5*, e1000475.

Sarkies, P., Reams, C., Simpson, L.J., and Sale, J.E. (2010). Epigenetic instability due to

defective replication of structured DNA. Mol. Cell *40*, 703–713.
Shishkin, A.A., Voineagu, I., Matera, R., Cherng, N., Chernet, B.T., Krasilnikova, M.M., Narayanan, V., Lobachev, K.S., and Mirkin, S.M. (2009). Large-scale expansions of Friedreich's ataxia GAA repeats in yeast. Mol. Cell *35*, 82–92.
Symington, L.S., Rothstein, R., and Lisby, M. (2014). Mechanisms and regulation of mitotic recombination in saccharomyces cerevisiae. Genetics *198*, 795–835.
Tang, W., Dominska, M., Gawel, M., Greenwell, P.W., and Petes, T.D. (2013). Genomic deletions and point mutations induced in Saccharomyces cerevisiae by the trinucleotide repeats (GAA·TTC) associated with Friedreich's ataxia. DNA Repair (Amst). *12*, 10–17.
Williams, J.D., Fleetwood, S., Berroyer, A., Kim, N., and Larson, E.D. (2015). Sites of instability in the human TCF3 (E2A) gene adopt G-quadruplex DNA structures in vitro. Front. Genet. *6*.
Yadav, P., Harcy, V., Argueso, J.L., Dominska, M., Jinks-Robertson, S., and Kim, N. (2014). Topoisomerase I Plays a Critical Role in Suppressing Genome Instability at a Highly Transcribed G-Quadruplex-Forming Sequence. PLoS Genet. *10*.

Supplementary Information 4. Potential G4 Kissing Loops detailed in 20 select LG4s. Chromosomal position and potentially interacting complementary loops are indicated.

hg38_chrX_510077_515075

```
>hg38_chrX_510077_515075
GGGAGCAGGAGCTCCTTCCCTCTCCCTGCAGCCCCTTTCCCTGTGGGAGCAGGAGCTCCTTCCCTCTCCCTGCATCCCCTTTCCCTGTGGGAGTGAG
AGCTCCTTCCCTCTCCCTGCATCCCCTTTCCCTGTGGGAGTGAGAGCTCCTTCCCTCTCCCTGCATCCCCTTTCCCTGTGGGAGTGAGAGCTCCTTC
CCTCTCCCTGCATCCCCTTTCCCTGTGGGAGCAGGAGCTCCTTCCCTCTCCCTGCATCCCCTTTCCCTGTGGGAGCAGGAGCTCCTTCCCTCTCCCT
GCAGCCCCTTTCCCTGTGGGAGCAGGAGCTCCTTCCCTCTCCCTGCATCCCCTTTCCCTGTGGGAGCAGGAGCTCCTTCCCTCTCCCTGCAGCCCCT
TTCCCTGTGGGAGTGAGAGCTCCTTCCCTCTCCCTGCAGCCCCTTTCCCTGTGGGAGTGAGAGCTCCTTCCCTCTCCCTGCATCCCCTTTCCCTGTG
GGAGCAGGAGCTCCTTCCCTCTCCCTGCATCCCCTTTCCCTGTGGGAGCAGGAGCTCCTTCCCTCTCCCTGCAGCCCCTTTCCCTGTGGGAGCAGGA
GCTCCTTCCCTCTCCCTGCATCCCCTTTCCCTGTGGGAGTGAGAGCTCCTTCCCTCTCCCTGCATCCCCTTTCCCTGTGGGAGTGAGAGCTCCTTCC
CTCTCCCTGCATCCCCTTTCCCTGTGGGAGTGAGAGCTCCTTCCCTCTCCCTGCATCCCCTTTCCCTGTGGGAGCAGGAGCTCCTTCCCTCTCCCTG
CATCCCCTTTCCCTGTGGGAGTGAGAGCTCCTTCCCTCTCCCTGCAGCCCCTTTCCCTGTGGGAGCAGGAGCTCCTTCCCTCTGCCTGCATCCCCTT
TCCCTGTGGGAGTGAGAGCTCCTTCCCTCTCCCTGCAGCCCCTTTCCCTGTGGGAGTGAGAGCTCCTTCCCTCTCCCTGCATCCCCTTTCCCTGTGG
GAGTGAGAGCTCCTTCCCTCTCCCTGCATCCCCTTTCCCTGTGGGAGCAGGAGCTCCTTCCCTCTCCCTGCATCCCCTTTCCCTGTGGGAGTGAGAG
CTCCTTCCCTCTCCCTGCATCCCCTTTCCCTGTGGGAGTGAGAGCTCCTTCCCTCTCCCTGCATCCCCTTTCCCTGTGGGAGCAGGAGCTCCTTCCC
TCTCCCTGCATCCCCTTTCCCTGTGGGAGTGAGAGCTCCTTCCCTCTCCCTGCATCCCCTTTCCCTGTGGGAGTGAGAGCTCCTTCCCTCTCCCTGC
ATCCCCTTTCCCTGTGGGAGTGAGAGCTCCTTCCCTCTCCCTGCATCCCCTTTCCCTGTGGGAGTGAGAGCTCCTTCCCTCTCCCTGCATCCCCTTT
CCCTGTGGGAGCAGGAGCTCCTTCCCTCTCCCTGCATCCCCTTTCCCTGTGGGAGCAGGAGCTCCTTCCCTCTCCCTGCATCCCCTTTCCCTGTGGG
AGTGAGAGCTCCTTCCCTCTCCCTGCAGCCCCTTTCCCTGTGGGAGCAGGAGCTCCTTCCCTCTCCCTGCATCCCCTTTCCCTGTGGGAGCAGGAGC
TCCTTCCCTCTCCCTGCATCCCCTTTCCCTGTGGGAGCAGGAGCTCCTTCCCTCTCCCTGCATCCCCTTTCCCTGTGGGAGTGAGAGCTCCTTCCCT
CTCCCTGCATCCCCTTTCCCTGTGGGAGTGAGAGCTCCTTCCCTCTCCCTGCAGCCCCTTTCCCTGTGGGAGCAGGAGCTCCTTCCCTCTCCCTGCA
TCCCCTTTCCCTGTGGGAGCAGGAGCTCCTTCCCTCTCCCTGCAGCCCCTTTCCCTGTGGGAGTGAGAGCTCCTTCCCTCTCCCTGCATCCCCTTTC
CCTGTGGGAGTGAGAGCTCCTTCCCTCTCCCTGCATCCCCTTTCCCTGTGGGAGTGAGAGCTCCTTCCCTCTCCCTGCAGCCCCTTTCCCTGTGGGA
GTGAGAGCTCCTTCCCTCTCCCTGCATCCCCTTTCCCTGTGGGAGCAGGAGCTCCTTCCCTCTCCCTGCATCCCCTTTCCCTGTGGGAGTGAGAGCT
CCTTCCCTCTCCCTGCATCCCCTTTCCCTGTGGGAGTGAGAGCTCCTTCCCTCTCCCTGCATCCCCTTTCCCTGTGGGAGTGAGAGCTCCTTCCCTC
TCCCTGCATCCCCTTTCCCTGTGGGAGTGAGAGCTCCTTCCCTCTCCCTGCATCCCCTTTCCCTGTGGGAGCAGGAGCTCCTTCCCTCTCCCTGCAT
CCCCTTTCCCTGTGGGAGTGAGAGCTCCTTCCCTCTCCCTGCATCCCCTTTCCCTGTGGGAGTGAGAGCTCCTTCCCTCTCCCTGCATCCCCTTTCC
CTGTGGGAGTGAGAGCTCCTTCCCTCTCCCTGCAGCCCCTTTCCCTGTGGGAGCAGGAGCTCCTTCCCTCTCCCTGCATCCCCTTTCCCTGTGGGAG
TGAGAGCTCCTTCCCTCTCCCTGCATCCCCTTTCCCTGTGGGAGCAGGAGCTCCTTCCCTCTCCCTGAATCCCCTTTCCCTGTGGGAGTGAGAGCTC
CTTCCCTCTCCCTGCATCCCCTTTCCCTGTGGGAGTGAGAGCTCCTTCCCTCTCCCTGCAGCCCCTTTCCCTGTGGGAGTGAGAGCTCCTTCCCTCT
CCCTGCAGCCCCTTTCCCTGTGGGAGTGAGAGCTCCTTCCCTCTCCCTGCATCCCCTTTCCCTGTGGGAGTGAGAGCTCCTTCCCTCTCCCTGCATC
CCCTTTCCCTGTGGGAGTGAGAGCTCCTTCCCTCTCCCTGCAGCCCCTTTCCCTGTGGGAGCAGGAGCTCCTTCCCTCTCCCTGCAGCCCCTTTCCC
TGTGGGAGTGAGAGCTCCTTCCCTCTCCCTGCATCCCCTTTCCCTGTGGGAGTGAGAGCTCCTTCCCTCTCCCTGCAGCCCCTTTCCCTGTGGGAGT
GAGAGCTCCTTCCCTCTCCCTGCATCCCCTTTCCCTGTGGGAGTGAGAGCTCCTTCCCTCTCCCTGCAGCCCCTTTCCCTGTGGGAGCAGGAGCTCC
TTCCCTCTCCCTGCATCCCCTTTCCC
```

(10 bp palindrome) extension for green

6bp palindrome

hg38_chr15_20300999_20305997

```
>hg38_chr15_20300999_20305997
CCCATCCTGACCCTGTCTTGGCCCTGTGCTACCCTGTCCTTGCCCTGGTCTTGCCCTGGCACTGGCCCTGCCCTGAACCTGCCCTGGCCTGACCTTG
GCTCTGGCCCTGGCTCTGGCCCTGCCCCTTGTCCTGACCCTGGTGCTGTCATGGCACTGGCCCTGCCAATGGTCATGGTCCTGCTCCTGTTCTGGCC
CTGACCTGGCCTTGGACATGTCCTGGCCCTGCTTTGGCCCATCCCTGCCCTGGCCCCACCATAGGCCTGCCTGTTCTACCCTCTCCTGGCACTGACC
TTGCCCTGTCATGGCCTAGTGGTGCCATTGCCCTGCCTTACCCTGTGCTGGTTGTGCCTTGGCCCTGCTTGGTGCTGGCCGCTCCCTGGACCTGCCC
TGACCCTGCCTTGGCTTTGCCCTGCCCTCACTATGGCCTGGCCCTGACCCTAGCCCTGGTCCTGCCATATCCCTGGCCCTGCCCTTATCCAGGCCC
TGGCCCTGCTGCTGGCCTGGAACCTGGTCCTGTCAAGGACCTGCCCTGACTCTGCCATGGCCCTGGCCCTGCTCTGCCTTGTTCCTGGCCCTGACCC
AGACCCAGACCCTTTCCTGGCTCTGCACTGGCCTTTCCGTGGTCCTGAGCTGGCAGTGGTCTGCCCCTGGTCTTGCCATCACCCTGCCCTGCTGTGC
TCTGGATGTGTCATCACCCTGACCTGGCCCTACTCTGCCTTTGACCCTGCCCTGGCCTTACCTTGGCCCTCACCCTAGTCTTCACTAGGCCCTGCTC
TGGAGCTGGCCCTAGCACAGACCTGGCCCTGACTCTGGCCCTGGTCTTTGTCCTGCCATAGCCCTGGCCCTGAAGTGAACTTCGAGGTGTCCTGGCC
CCGGCGTAACATAGCTCTGCATTGGCATGTCCCTGCCCTGCCGCTACCATTGCCTTGCCCTGCTCTGCCCTGTCCCAGTACTGACCTGGCCATGCTA
TTTCCCTGCCCTACCCTGCCTTGGCTGTGCCCTGGCTCGGTTCTGGCCCTGGCCCCGGCCCTGCCCTGGACATGCTCTGACACTGCCTCAGCCTCGG
CACTAGCCTGGCTCTTCTTTGGCATCAGCTCTGCTCTCTGTGTGGACCGGCTCTTGTCCTGTCCTGCACTGGCCATACCATGCCCTGCCCTGCCCTG
CCCGACTCAGCCCTGGCTCAGCCCTGGCCCAGCCTTGGCCTTGGCATTGCCCCTGGTCCTGCCATATTTCTTGCCCTGTCCCTACCCTGGCCTTGGC
CCTGACCCTTACCTTGCCCTGGCTCTGCCCTTGCCCTAACGCAGCCCCTGGCCCTGTCATGGCCCTGCCCTGGACCTGTCCTGGCCCTGGCCCTTCC
CTGCTTGAGACCTTGCCCTGGTTCTCCCCTGGCCCTGACCCTGAAATGCCTGGCCCTACCCTGGCCTTGCACTGCTCTGGCCCTTGCCCTGACTCTG
GTCCTGTCACTGGCCTAGCCCCAGCCCTGTTGCTGGTCTTACCATGGCCCAGACCCTGCCTTGGCCCTTCCCTGACACTGTCCTGGACCCTGGCTGT
GCCAAGATCCTGCACTGACCGTGTCCTTGTTTTGCTCCTGCCCCGAACCTGGTCCTGCCCAGGCCATGGCCATGGCCCTGGCCCTGCCCTGGCTGTT
CCCTGGCCCTGCCCTGCCTTGGCCCTATGCTTTCCTGGCCCTGTCTTGCCTGTCCTGGCCCTGCCTTGGCCCTAGCCTGGCTTTGACCCTGCCCTGG
CCCTACCTTGGCCTTCACCCTAGCCTTACCTGGCCACTCTGTTGGACCTGGCCATAGCACAGACCTGGTTGTGGCCCTGGCCCTGCCATGGCCCTGT
CCCAGACCCTAGCCCTGCCAGGTACCTGTCCTGGCCCAGCTCTGGGCCTGGCTTTGTCCCTGGTTCTTAGATGAACCTGGCCCTGCACCTGCCCTTG
CCCTTGCCCTGGCACTGGCCTTGGACATGTCCGTGGTCCTAACCCTGGCCCTGCCCTGGAGCTGCCACTGTCTTGGCCCTGCCCTGGCTCTGGCCCT
GCCCTGGCCCTGGCCCTGCCCCGGCCCCAGCCATAGACCTGCCCTGGTTGGTCATGCCCTACCTTAACCCTGTGCTACCCTGGGCCTGCTCCACCCT
GCCCTGGCCCTGCCCTCCCTTTGGCCCTGCCATGACCCTGCCTTGGCCCTCACACTGGCCCTAGCACAGACCTGGTCCTATCTGTGGCCTTAGCCTG
GCATTGACCCTTGCTCCTGACCCTGGCCCTGCCATGGCCCTTGCCCTGCCAATGACCCTGACAGCCCTGGCCCTGGCCCTGTCTTGGCCCTGGCCCT
GAACTGGCCCTGCCCTGACCCTGGCCCTGAAGTGGATTTGCAGGTGTCTTGTCCATGATTTAACCTGGTCTTATCATGGCCCTGTCCCTCCCCTGGC
TCTGTCCTGGTCTTGTGCTGACCCTGACCCAGACCTTGGCCCTGCCCCAGCCTTGTCCTTGACCTGGCCATGGCCCTGCCTCTGCCCTGGACCGAGG
CTGGCACTGGCATGGACCCTGGCCCTGGCCGTTCACTACTTAAGGCCATACCCTGGCCCAGCCCTGGTCCTGACCCTGTCCTGGCCCTAATTTGGCC
TGGCTCTACCCTGGCATGCTATTCTGGCCCTGGCCCTGACCCGGTCCCTGTCCCTGTCCTGGCCCCAGCCCCGTTGCTGGTCCTGCCATGGCCCTTG
TTCTGACATTGCCCTTTCCTGGTTCTGGCCCTGGCCCTGTCCCAGCCCTGCTCTGGCCCTGGTCTGAACCCTGGCCCTGCAATAGACCTGCCTTGGT
CCTGCCCAGACCCTGGCTCGGGCCCTACCTCTGCCCTGGCCATACCCTTGCCCTGGCCTGGACCTCGGTCCTGGTCCTTGTCCTGCCCCAGCCATGG
TCCTGGCCCTGCCCTGCCTGTGCCCTGTTCTATCCTGGGCTGGCCCTGCCATGGCCTGGTCTTGCCATTGCCCTGCCTTAGCCTGCCCTGCTTGTGC
CCTAGATCTGCCCCGGCCTTTGCCCCGTCTTGGTTCTAGCCTTGACGCAGCCCTGGACCTTCCCTGACCTTGCCTTAGCCCTGGCACTACCCTGGCC
TTGGCTTGGCATTTGCCCTACTCTATGGCCTGGCTCTCGTCCTGCCCTTATGCAGGCCTGACCCTGCCTGTGCCTTGGCTTTGGCCTGGACCTTGGC
CATACAGTGACCCTGCCATGACCCTTTCCTGGCCCTGGCCTGGAACCTGGCCCTGCCAAGGACTCGCCCTGGCTCTGTCATGGCCCTGGCCCTTTTC
TGGATTTGGATGTGTCCTGTCCCTTATTTCCCCGGCGCTTCCCTGGCTCTGCCATACCCC
```

- REVERSE COMP IN RED UNDERLINE

TGGCCTGGAACCTGG

- 6BP PALINDROME

TGCCTGTGCCTTGGCTTTGGCCTGGACCTTGGCCATACAGTGA

- REVERSE COMPLEMENT IN GREY

TGTGCTGGTTGTGCCTTGG

- REVERSE COMP IN PINK

TGCAATAGACCTGCCTTGGTCCTG

- REVERSE COMP IN MAROON

TGGCCTGGAACCTGG

- 6 BP PALINDROME

TGACTCTGCCATGG

- 8BP PALINDROME

TGCCATGGCCTGGTCTTGCCATTG

hg38_chr12_131390467_131395465

>hg38_chr12_131390467_131395465
GGGCATGGGGGGTGGACGTGGTGTGATATGGAGGGTGGGTGTGGGTGTGATGTGGAGGGTGGGTGTGGGTGTGATGTGGAGGGTGGGTGTGGTGTGA
TGTGGAGGGTGGGTGTGGTGTGATGTGGAGGGTGGGTGTGGTGTGATGTGGAGGGTGGGTGTGGTGTGATGTGCAGGGTGGGTGTGGGTGTGATGTG
GAGGGTGGGTGTGGTGTGATGTGCAGGGTGGGTGTGGGTGTGATGTGGAGGGTGGGTGTGGGTGTGATATGGAGGGTGGGTGTGGTGTGATGTGGAG
GGTGGGTGTGGTGTGATGTGGAGGGTGGGTTGGTGTGATGTGGAGGGTGGGTGTGGTGTGATGTGGAGGGTGGGTGTGGTGTGATGTGGAGGGTGGG
TGTGGGTGTGATGTGGAGGGTGGGTGTGGTGTGATGTGGAGGGTGGGTTGGTGTGATGTGGAGGGTGGGTGTGGTGTGATGTGGAGGGTGGGTGTGG
GTGTGATATGGAGGGTGGGTGTGGTGTGATGTGGAGGGTGGGTGTGGTGTGATGTGGAGGGTGGGTTTGTGTGATATGGGGGGTGGGTGTGGTGTGA
TGTGGAGGGTGGGTGTGGTGTGATGTGGAGGGTGGGTGTGGTGTCATATGGAGGGTGGGTGTGGTGCGATGTGGAGGGTGGGTGTGGTGTGATGTGG
AGGGTGGGTGTGGTGTGATGTGGAGGGTGGGTGTGGGTGTGATGTGGAGGGTGGGTGTGGGTGTGATGTGGAGGGTGGGTTGGTGTGATGTGGAGGG
TGGGTGTGGTGTGATGTGGAGGGTGGGTGTGGTGTGATGTGGAGGGTGGGTGTGGTGTGATGTGGAGGGTGGGTGTGGTGTGATATGGAGGGTGGGT
GTGGTGTGATGTGGAGGGTGGGTGTGGTGTGATATGGAGGGTGGGTGTCGTTTGATATGGAGGGTGGGTGTGATGTGGAGGGTGGGTGTGGTGTGAT
GTGGAGGGTGCGTGTGGTGTGATGTGGAGGGTGGGTGTGGTGTGATGTGGAGGGTGGGTGTGGTGTGATGTGGAGGGTGGGTGTGGTGTGATGTGGT
GGGTGGGTGGGTTTGATGTGGAGGGTGGGTGTGGTGTGATGTGGAGGTTGGGTGTTTTGTGATGTGGAGGGTGGGTTGGTGTGATGTGGAGGGTGGG
TGGGTGTGATGTGGAGGGTGGGTGTGGGTGTGATATGGAGGGTGGGTTGGTGTGATGTGGAGGGTGGGTGTGGTGTGATGTGGAGGGTGGGTGTGGT
GTGATATGGAGGGTGGGTTGGTGTGATGTGGAGGGTGGGTGTGGTGTGATGTGGAGGGTGGGTGTGGTGTGATGTGGAGGGTTGGTGTGGGTGTGAT
ATGGAGCTCCACCTGGG

Complementary to sequence highlighted in yellow.

TGTGGTGTGATGTGGAGGTT

Reverse complement to sequence highlighted in pink.

TGTGATATGGAGCTCCACCT

hg38_chr16_33710225_33715223

```
>hg38_chr16_33710225_33715223
CCCTGCCCCTGCCATGGATTTGGCCCTGTACTGCCCTGCCTTGCCCTGTACCTGCTCTACTGTTACCTGGACTCTCGGCCCTGTCCTGCTCTGGTCC
CATCCTGACTCTGTCTTGGCCCTGTGCTACCCTGTCCCTGCCCTGGTCTTACCCTGGCACTGGCCGTGCCCTGAACCTGCACTGGCCTGACCTTGGC
TCTGGCCCTGGCTCTGGCCCTGCCCCTTGTCCTGACCCTGGTCCTCTCGTGGCACTGGCCCTGCCAATGGTCATGGTCCTGCTCTTGTTCTGGCCCT
GACCTGGCCTTGGACATATCCTGGCCCTGCTTTGGCCCATCCCTGCCCTGGCCCCACCATGGGCCTGCCTTTTCTGCCCTCTCCTGGCACTGACCTT
GCCCTGTCATGGCCCAGTGGTGCCATTGTCCTGCCTTACCCTGCGCTGGTTGTGCCTTGGCCCCGCTTGGTGCTGGCCGCTCCCTGGACCTGCCCTG
ACCCTGCCTTGGCTTTTGCCCTGCCCTCACTATGGCCTGGCCCTGGCCCTAGCCCTGGTCCTGCCATATCCCTGGCCCTACCCTTATCCAGGCCCTG
CCCCTGTTGCTGCCCTGGCCCTGGCCTGGAACCTGGTCCTGTCAAGGACCTGCCCTGACTCTGCCATGGCCCTGGCCCTGCTCTGCCTTGTTCCTGG
CCCTGACCCAGACCCAGACCCTTTCCTCTCTCTGCACTGGCCTTTCCCTGGCCCTGAGCTGGCAGTGGTCTGCCCCTGGTCTTGCCATCACCCTGCC
CTGCTGTGCTCTGGATGTGTCATCCCCCTGCCCTGGCCCTACTCTGCCTTTGACCCTGCCCTGGCCTTACCTTGGCCCTCACCCTAGTCTTCATTAG
GCCCTGCTCTGGAGCTGGCCCTAGCACAGACCTGGCCCTGATCCTGGCTCTGGTCTTTGTCCTGCCATAGCCCTGGCCCTGAAGTGGACTTGGAGGT
GTCCTGGCCCCGGCATGACATGGCTCTGCATTGGCCTGTCCCTGCCCTGCCACTACCATCGCCTTTCCCTGCTCTGCCCTGTCCCAGTACTGACCTG
GCCATGCTATTTCCCTGCCGTACCCTGCCTTGGCTGTGCCCTGGCTCGGTTCTGGCCCTGGCCCCGGCCCTGCCCTGGATATGCTCTGACATTGCCT
CAGCCTTGGCACTAGCCTGGCTCTTTCTTGGCATCAGCCCTGCTGTCTCTGTGGACTGGCTCTTGTCCTGTCCTGCACTGGCCATACCATGCCCTGC
CCTGCCCTGCCCTGACTCAGCCCTGGCTCAGCCCTGGCCCAGCCTTGGCCTTGGCATTGCCCCTGGTCCTGCCATATTTCTTGCCCTGTCCCTACCC
TGGCCTTGGCCCTGACCCTTACCTTGCTCTGGCCCTGCCCTTACCCTAACACAGCCCCTGGCCCTGTCATGGCCCTGCCCTGGACCTGTCCTGGCCC
TGGCCCTTCCCTGCTTGAGACCTTGCCCTGGTTCTCCTCTGGCCCTGACCCTGAAATGCCTGGCCCTTCCCTGGCCTTGCACTGCTCTGGCGCTTGC
CCTGACTCTGGTCCTGTCACTGGCCTAGCCCCAGCCCTGTTGCTGGTCTTACCATGGCCCAGACCCTGCCTTGGCCCTGCCCTGACACTGTCCTGGA
CCCTGGCTGTGCCAAGAACGTGCACTGTCCTTGCCTTTGTTTTGCTCCTGCCCCAAACCTGGTCCTGCCCAGGCCCTGGCCCTGACCCTGACCTGGC
TGTTCCCTGGCCCTGCCCAGGTCTTGGCACTGGCCTGGCCCTGCCCTGCCTTGGCCCTATGCTTTCCTGGCCCTTCCTTGACGGCCCTGGCCCTGCC
TTGGCCCTAGCCTGGCTTTGACCCTGCCCTGGCCCTAGCCTGGCTTTGACCCTGCCCTGGCCCTACCTTGGCCTTCACCCTAGCCTTCCCTGGGCAC
TGTGTTGGACCTGGCCATAGCACAGACTTGGCTGTGGTCCTGGCCCTGCCATGGCCCTGTCCCAGACCCTAGCCCTGCCAGGTACCTGTCCTGGCCC
TGTTCTGGGCCTGGCTTTGTCCCTGGTTCTCAGATGACCCTGACCCTGCTTCTGCCCTTGCCCTTGCCCTGGCACTGGCCTTGGACATGTCCGTGGT
CCTAACCCTGGCCCTGCCCGGGAGCTGCCACTGTCTCGGCCCTGCCCTGGCTCTGGCCCTGCCCCAGCTCTGGCCCTGCCCTGGACCCAGCCATAGA
CCTGCCCTGGTTGGTCATGCCCTACCTTAACCCTGTGCTACCCTGGACCTGCTCCACCCTGCCCTCCCTTTGGCCCTGCCCTGAGCCCGCCTTGGCC
CACACACTGGCCCTAGCACAGACCTGGTCCTATCTGTGGCCTTGGCCTGGCATTGACCCCTGCTCCTGACCCTGGTCCTGCCATGGCCCTGGCCCTG
CCAATGACCCTGATGGCCCTGGCCCTGGCCCTGTCTTGGCCCTGGCCCTGAACTGGCCCTGCCCTGACCCTGGCCCTGAAGTGGATTTGCAGGTGTC
TTGTCCCTGAGTTAACCTGGTCCTACCATGGCCCTGTCCCTCCCCTGGCTCTGTCCTGGTCTTGTGCTGACCCTGACCCAGACCTTGGCCCTTCCCC
AGCCTTGTCCTAGACCTGGCCATGGCCCTGCCTCTGCCCTGGACCAGCGCTGGCACTGGCATGGACCCCCTGGCCCTGGCCCTTCGCTACTTAAGGC
CATACCCTGGCCCAGCCCTGGTCCTGACCCTGTCCTGGCCCTAATTTGGCCTGGCTCTACCCTGGCATGCTATTCTGGTCCTAGCCCTGACCCTGTC
CCTGTCCCTGTCCTGGCCCTAGCCCCGTTGCTGGTCCTGCCATGGTCCTTGTCCTGACATTGCCCTTTCCTGGTCCTGGCCCTGGCCCTATCCCAGC
CCTGGTCTGGCCCTGGTCTGAACCCTGGCCCTGCAATGGACCTGCCTTGGTCCTGCCCAGACCCTGGTTCTGGCCCTACCTCTGCCCTGGCCATACC
CTTGCCCTGGCCTGGACCCGGTCCTCGTCCTTGTCCTGCCCCAGCCGTGGCCCTGGCCCTGCCCTGCCTGTGCCCTGTTCTATCCTGGCCTGGCCTT
GCCATGGCCTGGTCTTGCCATTGCCCTGCCCTAGCCTGCCCTGCTTGTGCCCTAGATCTGCCCTGGCTTTGCCCCTGTCTTGGTTCCAGCCTTGAC
TCAGCCCTGGACCTTCCCTGACTTTGCCTCAGCCCTGGCACTACCCTGGCCTTGGCTTGGCATTTGCCCTACTCTCTCTATGGCCTGGCTCTGGTCC
TGCCCTGCTCTTGTTCTGTCCTGGCACAGCCCTGGCCCTGGCCCTGCCATATCACTGGCTCTGGTCCTGCCCTTATGCAGGCCTGACCCTGCCCCTG
CCTTGGCATTGGCCTGGACCTTGGCCGTACAGTGACCCTGCCATGACCCTTTCCTGGCCCTGGCCTGGAACTTGGCCCTGCCAAGGACTCGCCCTGG
CTCTGTCATGGCCCTGGCCCTTTCCTGGATTTGGATGTGTCCTGTCCCTTATTTGCCCCAGCCCTTCCCTGGCTCTGCCATACCC
```

>6bp palindrome highlighted in teal.

TGACTCTGCCATGG

>6bp palindrome highlighted in light green.

AGTACTGACCTGGCCATGCTATTT

>Complementary to sequence highlighted in pink.

TGGACCAGCGCTGGCACTGGCATGGA

>Complementary to sequence highlighted in yellow.

TGGTCCTGCCATAT

>Complementary to sequence highlighted in gray.

TGCCAGGTACCTGTCCTGG

>Complementary to sequence highlighted in dark green.

TAATTTGGCCTGGCTCTA

hg38_chr17_83002882_83004836

>hg38_chr17_83002882_83004836
CCCTCCGCGTGTGGGATTCCTGAGCCCGCCCTCCCGCCCTCCGCGTGTGGGATTCCTGAGCCCGCCCTCCCACTCTCCGCAGTCCGCGTGTGGGATT
CCTGAGCCCGCCCTCCCGCCCTCCGCGTGTGGGATTCCTGAGCCCGCCCTCCCGCCCTCCGCGTGTGGGATTCCTGAGCCCGCCCTCCCACTCTCCG
CAGTCCGCGTGTGGGATTCCTGAGCCCGCCCTCCCGCCCTCCGCGTGTGGGATTCCTGAGCTCGCCCTCCCACTCTCTGCAGTCCGCGTGTGGGATT
CCTGAGCCCGCCCTCCGCGTGTGGGATTCCTGAGCCCGCCCTCCCACTCTCTGCAGTCCGCGTGTGGGATTCCTGAGCCCGCCCTCCCGCCCTCCGC
GTGTGGGATTCCTGAGCCCGCCCTCCCGCCCTCCGCAGTCCGCGTGTGGGATTCCTGAGCCCGCCCTCCCGCCCTCCGCGTGTGGGATTCCTGAGCT
CGCCCTCCCACTCTCTGCAGTCCGCGTGTGGGATTCCTGAGCCCGCCCTCCCACTCTCCGCAGTCCGCGTGTGGGATTCCTGAGCCCGCCCTCCCGC
CCTCCGCGTGTGGGATTCCTGAGCTCGCCCTCCCACTCTCTGCAGTCCGCGTGTGGGATTCCTGAGCCCGCCCTCCCACTCTCTGCAGTCCGCGTGT
GGGATTCCTGAGCCCGCCCTCCCACTCTCTGCAGTCCGCGTGTGGGATTCCTGAGCCCACCCTCCCACTCTCTGCAGTCCGCGTGTGGGATTCCTGA
GCTCGCCCTCCCACTCTCTGCAGTCCGCGTGTGGGATTCCTGAGCCCGCCCTCCCGCCCTCCGCATGTGGGATTCCTGAGCCCGCCCTCCCGCCCTC
CGCGTGTGGGATTCCTGAGCCCGCCCTCCCACTCTCTGCAGTCCGCGTGTGGGATTCCTGAGCCCACCCTCCCACTCTCTGCAGTCCGCGTGTGGGA
TTCCTGAGCTCGCCCTCCCACTCTCTGCAGTCCGCGTGTGGGATTCCTGAGCTCGCCCTCCCGCCCTCCGCGTGTGGGATTCCTGAGCTCGCCCTCC
CACTCTCCGCAGTCCGCGTGTGGGATTCCTGAGCCCGCCCTCCCGCCCTCCGCATGTGGGATTCCTGAGCCCGCCCTCCCGCCCTCCGCGTGTGGGA
TTCCTGAGCCCGCCCTCCCACTCTCTGCAGTCCGCGTGTGGGATTCCTGAGCCCGCCCTCCCGCCCTCCGCGTGTGGGATTCCTGAGCTCGCCCTCC
CACTCTCCGCAGTCCGCGTGTGGGATTCCTGAGCCCGCCCTCCCGCCCTCCGCGTGTGGGATTCCTGAGCCCGCCCTCCCACTCTCCGCAGTCCGCG
TGTGGGATTCCTGAGCCCGCCCTCCCGCCCTCCGCGTGTGGGATTCCTGAGCCCGCCCTCCCACTCTCTGCAGTCCGCGTGTGGGATTCCTGAGCTC
GCCCTCCCGCCCTCCGCGTGTGGGATTCCTGAGCTCGCCCTCCCACTCTCTGCAGTCCGCGTGTGGGATTCCTGAGCCCGCCCTCCCGCCCTCCGCG
TGTGGGATTCCTGAGCTCGCCCTCCCACTCTCTGCAGTCCGCGTGTGGGATTCCTGAGCTCGCCCTCCCGCCCTCCGCGTGTGGGATTCCTGAGCTC
GCCCTCCCACTCTCTGCAGTCCGCGTGTGGGATTCCTGAGCTCGCCCTCCCACTCTCTGCAGTCCGCATGTGGGATTCCTGAGCTCGCCCTCCCACC
CTCCGCACTCCGCGTGTGGGATTCCTGAGCTCGCCCTCCCACCCTCCGCAGTCCGCGTGTGGGATTCCTGAGCCCGCCCTCCCACTCTCTGCAGTCC
GCGTGTGGGATTCCTGAGCCCGCCCTCCCACCC

Refined length: 1876bp
Bottom yellow is complementary to all other yellows.
Very last grey is complementary to all other greys.
ALL UNDERLINED ARE PALINDROME.
ALL GREENS ARE PALINDROME.
147 GGG, 491 SNP. No regulatory capacity. Overlaps with B3GNTL1. No 5’ or 3’ flank.
46 kissing loops.

hg38_chr5_391248_392495

>hg38_chr5_391248_392495
GGGGGGAGCTGAGGGCAGTCATGACACAGCCAGATTAGGAAGGTGGGCCAGAGCGTGCACGGGCGCAGGGCGAGGCGGGCGCAGGGCGAGGCAGGGC
CAGAGCGTGCATGGGCGCAGGGCGAGGAGGGCGCAGGGCGAGGCAGGGCCAGAGCGTGCACGGGGGCAGGGCGAGGCGGGCGCAGGGCGAGGCAGGG
CCAGAGCGTGCACGGGGGCAGGGCGAGGCGGGCGCAGGGTGAGGCAGGGCCAGAGCGTGCATGGGGGCAGGGCGAGGAGGGCGCAGGGCGAGGCAGG
GCCAGAGCGTGCACGGGGGCAGGGCGAGGAGGGCGCAGGGCGAGGCAGGGCCAGAGCGTGCACGGACACAGGGCGAGGAGGGCGCAGGGTGAGGCAG
GGCCAGAGCGTGCATGGGGGCAGGGCGAGGAGGGCGCAGGGTGAGGCAGGGCCAGAGCGTGCATGGGGGCAGGGCGAGGAGGGCGCAGGGCGAGGCA
GGGCCAGAGCGTGCACGGGGGCAGGGCGAGGAGGGCGCAGGGCGAGGCAGGGCCAGAGCGTGCACGGACACAGGGCGAGGAGGGCGCAGGGTGAGGC
AGGGCCAGAGCGTGCACGGGCGCAGGGCGAGGAGGGCGCAGGGTGAGGCAGGGCCAGAGCGTGCACGAACACACGGGTGCAGGGCGAGGCAGGCGCA
GGGCGAGGCAGGGCCAGAGCGTGCACGGACGCAGGGCGAGGAGGGCGCAGGGCGAGGCAGGGCCAGAGCGTGCACGGGCGCAGGGCGAGGAGGGCGC
AGGGTGAGGCAGGGCCAGAGCGTGCACGAACACACGGGTGCAGGGCGAGGCAGGCGCAGGGCGAGGCAGGGCCAGAGCGTGCACGGACGCAGGGCGA
GGAGGGCGCAGGGCGAGGCAGGGCCAGAGCGTGCACGGGCGCAGGGCGAGGAGGGCGCAGGGCGAGGCAGGGCCAGAGCGTGCACGGGCGCAGGGCG
ATGAGGGCGCAGGGCGAGGCAGGGCCAGAGCGTGCACGGGCGCAGGGCGAGGAGGGCGCAGGGCGAGGCAGGGCCAGAGCGTGCACGGGCGCAGGGC
GAGGAGGGCGCAGGGCGAGGCAGGGCCAGAGCGTGCACGGGCGCAGGGCGAGGAGGGCGCAGGGTGAGGCAGGGCCAGAGCGTGCACGAACACACGG
GTGCAGGGCGAGGCAGGCGCAGGGCGAGGCAGGGCCAGAGTGTGCACGGGCGCAGGGCGAGGTGGG

Refined length: 1230bp
Long palindrome.
115 GGG, 488 SNP. No regulatory capacity.
Overlaps with AHRR, no 5’ or 3’ flank.
19 kissing loops.

hg38_chr10_110226409_110227678

```
>hg38_chr10_110226409_110227678
GGGAGGGGTGTGCGCGCGTGAGGGTAGAGGAGCGCGCGTGTGTGGGGAGGGGCGTGTGGGGAGGGGAGCGCGCGTGAGGGGAGGGGCGTGTGAGGGA
GGGGTGCGCACGCGTGAGGGGAGCGCGCGTGTGAGGGGAGGGGCGTGTGAGGGAGGGGTGCGCGCGTGTGAGGGGAGGGGCGTGTGAGGGAGGGGAC
GCGCGTGTGAGGAGGGGCGTGTGGGTAGGGGAGCAGGCGTGTAAGGGGGGCGTGTGAGGAGCGCGCGCGTGAGGGGAGGGGCGAGTGAAGGAGGGGA
GCGCGCTTGTGATGGGAGGGGCATGTGGGGAAGGGAGCGCGCGTGTGGGAGGGGCGCACGCGTGAGGGGAGGGGCGTATGAGGGAAGGGGAGCGCGC
GTGAGGGGAGGGGCGTGCACATGTGAGGGGAGGAGTGTGTGAGGGGAGGGGAGCATGTGAGGGGAGGGGAGCTCGCCTGTGAGGGAGGGGTGCGCGC
GTGTGAGGGAGGGGTGCGCGCGTGTGAGGGGAGGGGCGTGTGAGGGGAGGGGAGCGCGCGCGTGAGGGGAGGGGCGCGCGGGTGTGAGGGGAGGGGC
GCGCGCGTGTGTGTGAGGGGAGGGGTGAGCGCATGTGAGGGGAGGGGTGCGAGCATGTGAGGGGAGGGGCGTACGCGTGTGGGGAGGGGCGTGTGAG
GGGAGGGCCATGCGTGTGTGTGAGGGCAGGGGTGCGCACGTTAGGGGAGGGGCGTGTGAGGAGAGGGGCACGCGCGTGTGTGGGGAGGGTCGCGCGT
GTGCGGGGAGGGTCGCGCGTGTGTGAGGGGTGCGCGCGTGGGAGGGATGGTGCGCGGGGGGGAGGGGCGTGTGCGGGAAGGGCGTGCGCGTGTGGGA
GGGGCGTGCGCGTGTGGGAGGGGCGGGTGCGGGGAGGGGCGCGCGTGCGTGGGGAGGTGTGTGTGCTGAGATCGTGAGTGGAGGCGTGTGTGAGAGG
TCGCGCAGGTGCTGGGGGAGGTGCGAGGGTGCGCACGGAGGGTGGGGCTGTGGGTGTGCGGCGAGCGGGAAGGAGACGGGTGGAGGGAAGTTGGAGA
GAGGGCGGTCTGGGGCTGGAGAGAGGGTGACCGTGGAGAAGCTTGGAGGGCCCCGGAGCGGGAGAGGGTGCTGGGAGATAAGAGGAAGGAGGAACAC
GGATGCTGGGGGGGGTCAGGGGAAGGGACGAGGCCGGGAGAACACTGAGCGGGGGAAAACCGGTGGAGAGGGGCCTTTGAATACCTGCGGGGTGATG
AGATGGGG
```

Each yellow highlight is a palindrome that binds to itself
Each underline is a palindrome that binds to itself
Each pink highlight can bind to each blue highlight
The green highlight can bind to each gray highlight

hg38_chr12_132222807_132227061

>hg38_chr12_132222807_132227061
CCCACACCCTACACAACCCGCACACCACACACACAGACACGCCACACAACACACATACACCCCACACAACCCGCACCCCACACCCCACATACATCCC
ACACAACCCACACCCCACACACACCACACAACTCACACCCCACACAACCCGCACCCCACACAACCCACACCCCACATACACCCCACACAACCCACAC
CCTACACAACCCGTACCCCACACACACAGACACACCACACAACCCACACCCCACATACACCCCACACACACACCCCACACCCTACACAACTCGCACC
CCAGACACCCCACACACACCCCACACACACACCACACAACCCACACACCACACAACCCACACCGCACACAACCCACACCCCACATACACCCTACACA
ACCCGCACCCCAGACACCCCACACAACCCACACCCCACATACACCCCACACACCCATCCTACACAACCCACACCCCACACAGACACCCCACACAACC
CACACCCCACATACACCCCACACACCCACCCCACACAACCCACACCCCACACACNCACCCCCCGCCACACACCACACAACCCACACCCTACACAACC
CGCACACCACACACACAGACACGCCACACAACACACATGCACCCCACACAACCCGCACCCCACACACACACAAACCCACACCCCACACAACCCACA
CCCCCATACACAGACACCCCACACACCCCACATACACCCCACACAACCCACACCCCACACAACCCACACCCCACACACACACACCACACAACCCACA
CCCCACAAACATACACCCCATACACCCCATATACACACCCCAGCGTACATACACACCCCACACTGTACCTATACACCCCACACTACACACCCCACAC
ACTGTGCATACACACCAGACATACGCCCCATACACCATACACACCCCACACCCCTCCCACACCCCACACTGTACACACCCCACACACATACATCTC
ATACACACACCCCACACACATACACACCCTCCCACATACACACCACACAACCCACACTCCACACACTGTAATGTACACACCCCACACATACCACAC
AATCCACACTGCACACACTGTATATACACGTGCCACATGCATAAACACAACCCACACCCCACACACTGTACATACACACCCCACACACATACAC
CCCATATACACACCCTATACACTATACGTACACCCCCCCACACACCACATAACCCACACCCCACACACTACATACACACCACCCCCCCACACACAA
CCCACATGCCACACACTGTACATACACACCCCCACATATACATACCACACAACTCACACCTCACACACTGTACACACCCTACACACATACCACAC
AACCCACACACTGTACATACACANCCCACACACATACACTGCATATACTCCATATACACATCCCATATACTATACTTATACACCCCCCACACACCA
CACAACCCACCCCACACTACATACACACCACCCCCACACAACCCACATGCCACACACTGTACATAAACACCCCCACATATACATACCACACAACTC
ACACCCCACACGCTGTACACACCCTACACACATACACACCACACAACCCACACACTATATACACCCCCCACACACACACCACACAACTGAAACTCC
ACTGTACATACACATCCCACACACATACATACCACACAATCCACACTCCACACACTGTATATACACATGCCACACACAACACACACCCCACACT
GTACATACACACCCCACACAACCCACACACCACATTCTATATATACACACCCCACACATGCACATCACACAACCCACACCCCACACACTGTATAT
ACACACCCCACACACACTCCACACACTGTACGTACACACCCCATACACACACTGTACGTACACACCCCATACACACAACACTGTACATACACAC
CCCCCACAACCCACACTACACACGCTGTATACACCCCACACACGCCACACAACCCACACCCCACTGTACATGCATACCCTCACACACACCACACAA
CCCACACCCCACACACTGTGTATACACACCCCCCATACACACCACACACCCCACAACCCACACCCCACACACTGTACATATACAACCCACACACAC
ACCCCATACACCCCATATGCACACCCCACACACTGTACACACCCCACACACATACACCCCACACACCCCACATTGTACACACCCCACATACACCC
CATACACACTATACACACACCCCACACTGTACATACACATCCCACACGCATACACCCCAAATACACACCCTACACACATACATCCCACACACCCCA
CACTGTACATACACACCCCACACATACACCCCATACACACCATATACACACCCCACACACATACACCCCACACTGTACATACACAACCCACACAT
ACACCCCATACACAACATATACACACACCCCACACACGGTACATACACACCCCACACATACACCTCATACACACCATATACACGCTGCACATACTGT
ACATACACCTCACACATACATCCCTTATACCCCATATACACATCCCACACACTGTACATACACATCCCACACACATACACCTCATATACCCCATG
TACACACCCCACACTGTACAGACATATCCCACACACATACACCCACCCCACACACTGTACACATGCACGCCACACATATGCCCCATACATACCATA
TACACACCCCACACACATACACAACACACAACCCACACCCCACTGTATATACACACCCACATACACCCCACACACATACACACCACACACCCCAC
ACCCCACTGTATATACACACCCCACATGCACCCCATGCACCCCCATATACACACCCCACACACACTGTACATACACACCAAACACCCACCCCAC
ACACTGTACATACACACCAAATACACATCCCACACACATACACCCCATACACCCACCCCACACACTGTACATACACATCCCACACATACACCCCA
TATACACACCCACACACATCCCATAAACACACCCCACACACACCATATACACACCCCATACACTGTACATACACACCCCACACACATACACTCCAT
AAACACGCCCCACACACACCATATACATACCCCACACACTGTACACACCCCACATACACCCCATACACACTGTATACACACCCCACACTGTACAT
ACACACCCATCCCCCCCACACACATACATACACCCCATACACACACACACCCACACCCACACACAACCC

The yellow highlight can bind with each green highlight
The gray highlights can bind with each teal highlight
Each red highlight is a palindrome that can bind to itself
Each underline can bind to each bold
Each italicize is a palindrome that can bind to itself

hg38_chr17_170672_172022

>hg38_chr17_170672_172022

GGGGTGAGGGGAAGGGAACAGGACAGTTGAGAACATGGAGCTGACACATGCTTGAGTGGCAGAGCCAGAGGGC
AGCACCAGGGACCAGGCCAGGCTGCAGAGGGGAGCACCAGGGGCCGGGCCAGGCTGCAGAGGGCAGCACCAGG
GGCCGGGCCAGGCTGCAGAGGGCAGCACCAGGGGCCGGGCCAGGCTGCAGAGGGGAGCACCAGGGGCCGGGCC
AGGCTGCAGAGGGCAGCACCAGGGGCCGGGCCAGGCTGCAGAGGGCAGCACCAGGGGCCGGGCCAGGCTGCAG
AGGGGAGAGCACCAGGGGCCGGGCCAGGCTGCAGAGGGGAGAGCACCAGGGGCCGGGCCAGGCTGCAGAGGG
GAGCACCAGGGGCCGGGCCAGGCTGCAGAGGGCAGCACCAGGGGCCGGGCCAGGCTGCAGAGGGGAGCACCAG
GGGCCGGGCCAGGCTGCAGAGGGGAGCACCAGGGGCCGGGCCAGGCTGCAGAGGGGAGAGCACCAGGGGCCG
GGCCAGGCTGCAGAGGGGAGAGCACCAGGGGCCGGGCCAGGCTGCAGAGGGGAGAGCACCAGGGGCCGGGCCA
GGCTGCAGAGGGCGGCACCAGGGGCCGGGCCAGGTTGCAGAGGGGAGCACCAGGGGCCGGGCCAGGCTGCAGA
GGGGAGCACCAGGGGCCGGGCCAGGCTGCAGAGGGCAGCACCAGGGGCCGGGCCAGGCTGCAGAGGGGAGAGC
ACCAGGGGCCGGGCCAGGCTGCAGAGGGGAGCACCAGGGGCCGGGCCAGGCTGCAGAGGGCAGCACCAGGGGC
CGGGCCAGGCTGCAGAGGGGAGAGCACCAGGGGCCGGGCCAGGCTGCAGAGGGGAGCACCAGGGGCCGGGCCA
GGCTGCAGAGGGGAGCACCAGGGGCCGGGCCAGGCTGCAGAGGGGAGAGCACCAGGGGCCGGGCCAGGCTGCA
GAGGGGAGCACCAGGGGCCGGGCCAGGCTGCAGAGGGCAGCACCAGGGGCCGGGCCAGGCTGCAGAGGGGAGC
ACCAGGGGCCGGGCCAGGCTGCAGAGGGCAGCACCAGGGGCCGGGCCAGGCTGCAGAGGGGAGAGCACCAGGG
GCCGGGCCAGGCTGCAGAGGGGAGCACCAGGGGCCGGGCCAGGCTGCAGAGGGCAGCACCAGGGGCCGGGCCA
GGCTGCAGAGGGGAGAGCACCAGGGGCCGGGCCAGGCTGCAGAGGGGAGCACCAGGGGCCGGGCCAGGCTGCA
GAGGGGAGCACCAGGGGCCGGGCCAGGCTGCAGAGGGGAGCACCAGGGGCCGGGCCAGGCTGCAGAGGGGAG
CACCAGCGGCCGGGCCAGGCTGCAGAGGGG

>ACCAGGCCAGGCTGCAGA

(38 hits- green highlighted sequence is a palindrome)

>CCAGGCTGCAGA

(38 hits- green highlighted sequence is a palindrome)

hg38_chr16_89618874_89620033

>hg38_chr16_89618874_89620033

CCCTCTACCTTTGGCTTCCGGAGGGTTGCCCTCCCTGCTCCCTCCCTGCAGCTCCCTGCCCCCCTGCTCCCCTCCCT
GCAGCCCCCCTGCCCCCCCGCTCCCCTCCCTGCAGCCCCCTGCCCCCCCCGCTCCCCTCCCTGCAGCTCCCTGCCC
CCCTGCTCCCCTCCCTGCAGCCCCCTGCCCCCCCTGCTCCCCTCCCTGCAGCTCCCTGCCCCCCTGCTCCCCTCCCT
GCAGCTCCCTGCCCCCCCTGCTCCCCTCCCTGCAGCCCCCTGCCCCCCCGCTCCCCTCCCTGCAGCTCCCTGCCCCC
CCGCTCCCCTCCCTGCAGCCCCCCTGCCCTCCCTGCTCCCTCCCTGCAGCCCCCTGCCCCCCCACTCCCCTCCCTGC
AGCTCCCTGCCCCCCCGCTCCCCTCCCTGCAGCCCCCTGCCCCCCCGCTCCCCTCCCTGCAGCTCCCTGCCCCCCCG
CTCCCCTCCCTGCAGCCCCCTGCCCCCCCTGCTCCCCTCCCTGCAGCCCCCTGCCCCCCCTGCTCCCCTCCCTGCAG
CCCCCTGCCCCCCTGCTCCCCTCCCTGCAGCCCCCTGCCCCCCTGCTCCCCTCCCTGCAGCCCCCTGCCCCCCTGCT
CCCCTCCCTGCAGCTCCCTGCCCCCCTGCTCCCCTCTCTGCAGCCCGCTTTCCCCCTGCTCCCCCCGCTCCCCTCCC
TGCAGCCCCCTGGTCCCCCTGCTCCCCCCGCTCCCCTCCCTGCAGCCCCCTGCTCCCTCTGCTCCCCTCCCTGTAGC
TCCCTGCAGCCCCCTGCTCCCCTCCCTGCACCCCTTTTGCCCCCTGCTCCCCTCCCTGCAGCCCCCTGCACTCCCTT
CACCCCCTCCCTGCAGCCTGCTGTGCCCCCTGCTCCCCTCCCTGCAGCCCCCTGCACCCCCTCCCTGCAGCGCCCT
GTGCCCCCCACTCCCCTCTCTGCAGCCCCCTGCGTCCCCTCCCCTCTCTGCAGCCCCCACTGTTCCTCCCACTGCTG
CCCATCATTGGCTGCTTGAAAATGTCAGACTCCAAGGGCCCCTGTGACTAGCCCAGCAGCAGCCCTAGGTCTCCA
CCCTGCCTTGGGTGGGGTCCGGGATAGGCCTGGCCTCCCCCCGCCTGCTTGAACCCACTGAGTATGCCCCCCTCC
C

>TCTCTGCAG

(30 hits-dark green highlighted sequence CTGCAG is a palindrome)

>TGCAGCCTGCTGTG

(22 hits – purple highlighted sequence is not a palindrome just repeats. Yellow GGCTGC sequence is the reverse compliment of the purple GCAGCC)

>ACTGCTG

(2 hits - light green highlighted sequence CTGCTG is not a palindrome, just repeats. Dark blue CAGCAG is the reverse compliment of CTGCTG)

>

TGCAGCCTGCTGTG

hg38_chr2_3436705_3438617

```
>hg38_chr2_3436705_3438617 #1
CCTGTGTATTAATCCCCCCACCACCCCTGGATTAATACCCCCATCACCCCTGGATTAATCTCCCCACTACCCCTGGATTAATACCCCATCACCCCTG
AATTAATCCCCCCATCACCCCAGGATTAATACCCCATCACCCCAGGATTAATACCCCATCACCCCTGGATTAATCTCCCCACCACCCCTGGATTAAT
CTCCCCACCACCCCTGGATTAATCCCCCCACCACCCCTGGATTAATCCCCCAATCACCCCTGGATTAATCCCCCAATCACCCCTGGATTAATCCCCC
CATCAGCCCTGGATTAATACCCCATCACCCCAGGATTAATACCCCATCACCCCTGAATTAATCCCCCCACCACCCCTGGATTAATCCCCCAATCACC
CCTGGATTAATCCCCCCATCAGCCCTGGATTAATACCCCATCACCCCAGGATTAATACCCCATCACCCCTGAATTAATCCCCCCACCACCCCTGGAT
TAATCCCCCAATCACCCCTGGATTAATCCCCCCATCACCCCTGGATTAATGCCCCATCACCCCTGGATTAATCCCCCAATCACCCCTGGATTAATCC
CCCCATCACCCCTGGATTAATACCCCATCACCCCTGAATTAATCCCCCCACCACCCCTGGATTAATCCCCCAATCACCCCTGGATTAATCCCCCCAT
CACCCCTGGATTAATACCCCATCACCCCTGAATTAATCCCCCCACCACCCCTGGATTAATCCCCCAATCACCCCTGGATTAATCCCCCCATCACCCC
TGGATTAATACCCCATCACCCCTGGATTAATACCCCATCACCCCTGGATTAATCCCCCCACCACCCCTGGATTAATCCCCCAATCACCCCTGGATTA
ATCCCCCCATCACCCCAGGATTAATACCCCATCACCCCAGGATTAATCCCCCAATCACCCCTGGATTAATCCCCCCATCACCCCTGGATTAATACCC
CATCACCCCTGGATTAATCCCCCCATCACCCCTGGATTAATCCCCCAATCACCCCTGGATTAATCCCCCCATCACCCCTGGATTAATACCCCATCAC
CCCTGGATTAATCCCCCAATCACCCCTGGATTAATCCCCCCATCACCCCTGGATTAATACCCCATCACCCCTGGATTAATCCCCCCATCACCCCTGG
ATTAATCCCCCATCACCCCTGGATTAATACCCCATCACCCCTGGATTAATCCCCCCACCACCCCTGGATTAATCCCCCAATCACCCCTGGATTAATC
CCCCCATCACCCCAGGATTAATACCCCATCACCCCAGGATTAATACCCCATCACCCCTGGATTAATCCCCCCATCACCCCTGGATTAATCCCCCAAT
CACCCCTGGATTAATCCCCCCATCACCCCAGGATTAATACCCCATCACCCCAGGATTAATACCCCATCACCCCTGGATTAATCCCCCCACCACCCCT
GGATTAATCCCCCAATCACCCCTGGATTAATCCCCACCACCCCTGGATTCATCCCCCACCACCCCTGGATTCATCCCCCCATCAGCCCTGGATTAGT
ACTTCCACCCCTGGATAAATCCCCCCACCATCCCTGGATTAACCCCCATCACCCCTGGATTAGCCCCCATCACCCCTGGGTTAGCCCCCATCACCCC
TGGATTAATACTTCCACCCCTGGATTGATCCCCCATCACCCCTGGATTAATCCCCCATCACGCCTGGATTAATACACCCACTACCTCTGGATTAACC
CCCATCACCCCTGGATTAATACCCCCACCAGCCCTGGATTAATCCCCCGCCACCCCTGGATCAATCCCCCCAATCCCCCTGGGTTAATCCCCCACCAC
CCGTGGATTGATCCCCCCTCACCCCTGGATTAATCCCCCCATCCCCCTGGGTTAATCCCCCATCACCCCT
```

Green-green (palindrome): 72 hits
Yellow-yellow (palindrome): 41 hits
Purple-Dark Blue:3 hits
Base-Pair Length: 1,913

hg38_chr18_76392495_76394852

>hg38_chr18_76392495_76394852 #8
GGAAGGCAGGTGGGAAGGTAGGTGGCCAGGTGGGGGAAAGGTGGATGGGAAGGCAGGTGGCCACGTGGGGGAAAGGTGGATGGGAAGGCAGGTGGCC
GGGTGGGGGAAGGGCAGGTGGGAAGACAAGTGGCCAGGTGGAGGGAGGGCAGGCGGGAAGGCAGGTGACCAGGTGGGGGAAAGGTGGATGGGAAGGC
AGGTGACCAGGTGGGGGAAAGGTGGATGGGAAGGCAGGTGACCAGGTGGGGGAAAGGTGGATGGGAAGGCAGGTGACCAGGTGGGGGAAAGGTGGAT
GGGAAGGCAGGTGGCCAGGTGGGGGAAAGGCAGGTAGTCAGGTGGGAAGCCGGGAAGGCAGGTGGTCAGGTGGGGGGAAGGCAGGTGGTCAGGTGGG
GGGAAGGCAGGTGGTCAGGTGGGGGGAAGGCAGGTGGTCAGGTGGGGGGAGGGCAGGTGGTCAGGTGGGGGGAAGGCAGGTGGTCAGGTGGGGGGAA
GGCAGGTGGTCAGGTGGGGGGAGGGCAGGTGGTCAGGTGGGGGGAAGGCAGGTGGTCAGGTGGGGGGAAGGCAGGTGGTCAGGTGGGGGGAAGGCAG
GTGGTCAGGTGGGGGGAGGGCAGGTGGTCAGGTGGGGGGAGGGCAGGTGGTCAGGTGGGGGGAAGGCAGGTGGTCAGGTGGGGGGAAGGCAGGTGGT
CAGGTGGGGGCAGGGCGGGTGGTCAGGTGGGGGGAAGGCAGGTGGTCAGGTGGGGGGAGGGCAGGTAGTCAGGTGGGAAGCCGGGAAGGCAGGTGGT
CAGGTGGGGGGAAGGCAGGTGGTCAGGTGGGGGGAAGGCAGGTGGTCAGGTGGGGGGAGGGCAGGTGGTCAGGTGGGGGGAAGGCAGGTGGTCAGGT
GGGGGGAGGGCAGGTGGTCAGGTGGGGGGAAGGCAGGTGGTCAGGTGGGGGGAAGGCAGGTGGTCAGGTGGGAAGCCGGGAAGGCAGGTGGTCAGGT
GGGGGCAGGGCGGGTGGTCAGGTGGGGGGAGGGCAGGTGGTCAGGTGGGGGGAAGGCAGGTGGTCAGGTGGGGGCAGGGCGGGTGGTCAGGTGGGGG
GAAGGCAGGTGGTCAGGTGGGGGCAGGGCGGGTGGTCAGGTGGGGGGAGGGCAGGTGGTCAGGTGGGGGGAAGGCAGGTGGTCAGGTGGGGGCAGGG
CGGGTGGTCAGGTGGGGGGAAGGCAGGTGGTCAGGTGGGGGGAAGGCAGGTGGTCAGGTGGGGGGAAGGCAGGTGGTCAGGTGGGGGCAGGGCGGGT
GGTCAGGTGGGGGGAAGGCAGGTGGTCAGGTGGGGGGAAGGCAGGTGGTCAGGTGGGGGGAAGGCAGGTGGTCAGGTGGGGGCAGGGCGGGTGGTCA
GGTGGGGGGAGGGCAGGTAGTCAGGTGGGAAGCCGGGAAGGCAGGTGGTCAGGTGGGGGGAAGGCAGGTGGTCAGGTGGGGGGAAGGCAGGTGGTCA
GGTGGGGGGAGGGCAGGTGGTCAGGTGGGGGGAAGGCAGGTGGGGGGAAGGCAGGTGGTCAGGTGGGGGGAGGGCAGGTGGTCAGGTGGGGGGAAGG
CAGGTGGTCAGGTGGGGGGAAGGCAGGTGGTCAGGTGGGAAGCCGGGAAGGCAGGTGGTCAGGTGGGGGCAGGGCGGGTGGTCAGGTGGGGGGAGGG
CAGGTGGTCAGGTGGGGGGAAGGCAGGTGGTCAGGTGGGGGCAGGGCGGGTGGTCAGGTGGGGGGAGGGCAGGTAGTCAGGTGGGAAGCCGGGAAGG
CAGGTGGTCAGGTGGGGGGAAGGCAGGTGGTCAGGTGGGGGGAAGGCAGGTGGTCAGGTGGGGGGAGGGCAGGTGGTCAGGTGGGGGGAAGGCAGGT
GGTCAGGTGGGGGGAGGGCAGGTGGTCAGGTGGGGGGAAGGCAGGTGGTCAGGTGGGGGGAGGGCAGGTGGTCAGGTGGGGGGAAGGCAGGTGGTCA
GGTGGGGGGAAGGCAGGTGGTCAGGTGGGAAGCCGGGAAGGCAGGTGGTCAGGTGGGGGCAGGGCGGGTGGTCAGGTGGGGGGAGGGCAGGTGGTCA
GGTGGGGGGAAGGCAGGTGGTCAGGTGGGGGCAGGGCGGGTGGTCAGGTGGGGGGAAGGCAGGTGGTCAGGTGGGGGGAAGGCAGGTGGTCAGGTGG
GGGCAGGGCGGGTGGTCAGGTGGGGGGAAGGCAGGTGGTCAGGTGGGGGGAAGGCAGGTGGTCAGGTGGGGGGAAGGCAGGTGGTCAGGTGGGGGCA
GGGCGGGTGGTCAGGTGGGGGGAGGGCAGGTGGTCAGGTGGGGGGAAGGCAGGTGGTCAGGTGGGGGCAGGGCGGGTGGTCAGGTGGGGGGAAGGCG
GGTGGTCAGGTGGGGGGAGGGCAGGTGGGA

Green-pink: 86 hits
Teal-Yellow: 4 hits
Base-pair length: 2,358

hg38_chr16_89852017_89853089

```
>hg38_chr16_89852017_89853089
CCCCCACCCCCCAGATCCCCTGGCCCATCTTCCATCCTCCCCCAACCCGCCAGATCCCCTGGCCCATCTTCCATCCTCCCCCCACCCACCAGATCCC
ATGGCCCATCTTCCATCCTCCCCCCACCCCCCAGATCCCATGGCCCATCTTCCATCCTCTCCCCACCCACCAGATCCCATGGCCCATCTTCCATCCT
CCCCCCACCCCCCAGATCCCATGGCCCATCTTCCATCCTCCCCCCACCCACCAGATCCCATGGCCCATCTTCCATCCTCTCCCCACCCACCAGATCC
CATGGCCCATCTTCCATCCTCCCCCCACCCCCCATATTCCATGGCCCATCTTCCATCCTCCCCCCACCCCCCAGATCCCATGGCCCATCTTCCATCC
TCCCCCCACCCCCCAGATCCCATGGCCCATCTTCCATCCTCTCCCCACCCACCAGATCCCATGGCCCATCTTCCATCCTCCCCCCACCCCCCAGATC
CCATGGCCCATCTTCCATCCTCCCTCCACCCCCCGGATCCCCTGGCCCATCTTCCGTCCTCTCCCCACCCCCTGGATCCCCTGGCCCATCTTCCATC
CTCCCTCCACCCCCCAGATCCCCTGGCCCGTCTTCCGTCCTCCCTCTCACCCCCCGGATCCCATGGCCCGTCATCCGTCTTCTCTCTCACCCCCCGG
ATCCCATGATCCGTCTTCCGTCCTCCCTCTCACCCCCCGGATCCCATGGCCCGTCTTCCGTCCTCCCTCTCACCCCCCGGATCCCATGGCCCGTCTT
CCGTCCTCCCTCTCACCCCCCGGATCCCATGGCCCGTCTTCCGTCCTCCCTCTCACCCCCCGCATCCCATGGCCCCTCTTCCGTCCTCCCTCTCACC
CCCCGGATCCCATGGCCCGTCTTCCGTCCTCCCTCTCACCCCCCGCATCCCATGGCCCGTCTTCCATCCTCCCTCTCACCCCCCGGATCCCATGGCC
CGTCTTCCATCCTCCCTCTCACCCTCAAGATCCCATGGCCCATCTTCCGTCCTCTCTCTCAGCTCCCCCTTGGCCTCCTGGGACATGCTTCTAACGT
TCCCCA
```

CCATGG= palindromes 20 hits

hg38_chr5_83036_85631

GGGTGTCCATTGTGTGCTGGGCACCATTCTAGGGGCTAATATTCCAGTGGGAGAGAGAGATGCTAAACATGAAACTATAAACTGGGTATCAGCATAA
GTGATTCACAGGGTATGTTACTGGGAAGCCTCGTTGAGAGGCCGGGTGGTCAGGGAAGCCTCGATCAGAGGGAGGGTGGTCAGGGAAGCCTTGATGA
GAGGCCAGGTAGTCAGGGAAGCCTTGATGAGAGGGAGGGTGGTCAGGGAAGCCTCCATGAGAGGCCAGGTGGTTGGGGAAGCCTCGATGAGAGGCCG
GGTGGTCGGGGAAGCCTCGATGAGAGGCCGGGTGGTCGGGGAAGGCTCGATGAGAGGCAGGGTGGTCGGGGAAGCTTCGATGAAAGGCTGGGTGGTC
GGGGAAGGCTCGATGAGAGGCCGGGTGGTCAGGGAAGGCTCGATGAGAGGCCGGGTGGTCAGGGAAGCCTCGATGAGAGGCCGGGTGGTCGGGGAAG
CCTCGATGAGAGGCCGGGTGGTCGGGGAAGCCTCGATGGGAGGCCGGGTGGTCGGGGAAGGCTCGATGGGAGGCAGGGTGGTCGGGGAAGGCTCGAT
GAGAGGCCGGGTGGTCGGGGAAGCCTCGATGAGAGGCCGGGTGGTCGGGGAAGCCTTGATGAGAGGCCGGGTGGTCAGGGAAGGCTCGATGAGAGGC
CGGGTGGTCGGGGAAGCCTCGATGGGAGGCCGGGTGGTCGGGGAAGCCTTGATGAGAGGCCGGGTGGTCAGGGAAGGCTCGATGAGAGGCCGGGTGG
TCGGGGAAGCCTCGATGAGAGGCCGGGTGGTCGGGGAAGCCTCGATGGGAGGCAGGGTGGTCGGGGAAGCCTCGATGGGAGGCCGGGTGGTCGGGGA
AGCCTCGATGAGAGGCCGGGTGGTCAGGGAAGGCTCGATGGGAGGCAGGGTGGTCGGGGAAGCCTCGATGGGAGGCCGGGTGGTCGGGGAAGCCTCG
ATGAGAGGCCGGGTGGTCAGGGAAGCCTCGATGGGAGGCCGGGTGGTCGGGGAAGCCTCGATGGGAGGCAGGGTGGTCGGGGAAGCCTCGATGGGAG
GCAGGGTGGTCGGGGAAGCCTCGATGGGAGGCAGGGTGGTCGGGGAAGCCTCGATGGGAGGCAGGGTGGTCAGGGAAGGCTCGATGAGAGGCCGGGT
GGTCGGGGAAGCCTTGATGGGAGGCCGGGTGGTCGGGGAAGCCTCGATGAGAGGCCGGGTGGTCGGGGAAGCCTTGATGAGAGGCCGGGTGGTCGGG
GAAGGCTCGATGAGAGGCCGGGTGGTCGGGGAAGCCTCGATGGGAGGCCGGGTGGTCGGGGAAGCCTCGATGGGAGGCAGGGTGGTCGGGGAAGCCT
CGATGGGAGGCCGGGTGGTCGGGGAAGCCTCGATGAGAGGCCGGGTGGTCAGGGAAGCCTCGATGAGAGGCCGGGTGGTCGGGGAAGCCTCGATGAG
AGGCCGGGTGGTCAGGGAAGGCTCGATGAGAGGCAGGGTGGTCGGGGAAGCCTCGATGGGAGGCAGGGTGGTCGGGGAAGCCTCGATGGGAGGCCGG
GTGGTCGGGGAAGGCTCGATGAGAGGCCGGGTGGTCGGGGAAGCCTCGATGAGAGGCCGGGTGGTCGGGGAAGCCTCGATGAGAGGCCGGGTGGTCA
GGGAAGGCTCGATGAGAGGCAGGGTGGTCGGGGAAGCCTCGATGGGAGGCAGGGTGGTCGGGGAAGCCTCGATGGGAGGCAGGGTGGTCGGGGAAGC
CTCGATGGGAGGCAGGGTGGTCGGGGAAGCCTCGATGGGAGGCAGGGTGGTCAGGGAAGGCTCGATGAGAGGCCGGGTGGTCGGGGAAGCCTTGATG
GGAGGCCGGGTGGTCGGGGAAGCCTCGATGAGAGGCCGGGTGGTCGGGGAAGCCTTGATGAGAGGCCGGGTGGTCGGGGAAGGCTCGATGAGAGGCC
GGGTGGTCGGGGAAGCCTCGATGGGAGGCAGGGTGGTCGGGGAAGCCTCGATGGGAGGCAGGGTGGTCAGGGAAGGCTCGATGAGAGGCCGGGTGGT
CGGGGAAGCCTCGATGGGAGGCCGGGTGGTCGGGGAAGCCTCGATGGGAGGCAGGGTGGTCGGGGAAGCCTCGATGGGAGGCAGGGTGGTCAGGGAA
GGCTCGATGAGAGGCCGGGTGGTCGGGGAAGCCTTGATGGGAGGCCGGGTGGTCGGGGAAGCCTCGATGAGAGGCCGGGTGGTCGGGGAAGCCTCGA
TGAGAGGCCGGGTGGTCGGGGAAGCCTCGATGAGAGGCCGGGTGGTCGGGGAAGCCTTGATGAGAGGCCGGGTGGTCAGGGAAGGCTCGATGAGAGG
CCGGGTGGTCGGGGAAGCCTCGATGGGAGGCAGGGTGGTCGGGGAAGCCTCGATGGGAGGCAGGGTGGTCAGGGAAGGCTCGATGAGAGGCCGGGTG
GTCAGGGAAGCCTCGATGAGAGGCAGCGAGCAGACATACGCAGGAGGGAGGGGTCGGGCCAGGCAGAGATCGGGGAGTGTGTGTGGGGCAGAGGGAA
CTGTGGGTGAAGTTCTGTGTCTGAGGTGTGCCTGGCATGTGGGGAGGCCTGTGGCTGGAGGAGAGGCATTGGGG

- 29 kissing loops
- Promoter flank

hg38_chr11:1351757-1353522

CCCTCTGCCTCTGCTAGCTTTTCTCCCAGGTCTCACACCTGGACCCCCCACCCCCACCAACTCCCCTCCCAGGCCTCCCACCCAGACCCCCACTCCC
CTCCTAGGCCTCCCACCTAGACCCCTGCCCCCACCAATTCCCCTTCCAGGCCTCACACCTGGACCCCCACCCCCACTGACTCCCCTCCCAGGCCCCA
TGCCTGGACCCCCACCAACTCCCCTCCCAGGCCTCATACCTGGACCCCCCACCCCCACCAACTCCCCTCCCAGGCCTCACACCCGGACCCCCACCCA
CTCCCCTTCTAGGCCTCCCACCTAGACCCCTGCCCCCACCAATTCCCCTTCCAGGCCTCACACCTGGAACCCCACACCCACCAACTCCCCTCCCAGG
CCTTACACCTGGACCCCCACCCCCACTGACTCCCCTCCCAGGCCCCATGCCTGGACCCCCACCCCCACCAACTCCCCTCCCAGGCCTCACACCTGGA
CCCCCCACCCCCACCAACTTCCATCCCAGGCCTCACACCTGGACCCCCACCCCCACCAACTCCCCTCCCAGGCCTCATACCTGGACCCCCCACCCCC
ACCAACTCCCCTCCCAGGCCTCATACCTGGACCCCCCACCCCCACCAACTCCCCTCCCAGGCCTCACACCTGGACCCCCCACCCCCACCAACTCCCC
TCCCAGGCCTCACACCCGGACCCCCACCCACTCCCCTTCTAGGCCTCCCACCTAGACCCCTGCCCCCACCAATTCCCCTTCCAGGCCTCACACCTGG
AACCCCACACCCACCAACTCCCCTCCCAGGCCTTACACCTGGACCCCCACCCCCACTGACTCCCCTCCCAGGCCCCATGCCTGGACCCCCACCCCCA
CCAACTCCCCTCCCAGGCCTCACACCTGGACCCCCCACCCCCACCAACTTCCATCCCAGGCCTCACACCTGGACCCCCACCCCCACCAACTCCCCTC
CCAGGCCTCACACCTCGACCCCCCACCCCCCACCCCTCATCCCCACTGACTCCCTTCCCAGGCCTCACACCTGGACCCCCCACCCCCCACCCCCACC
AACTTCCATCCCAGGCCTCACACCTGGACCCCCACCCCCACCCCTCATCCCCACCGACTCCCCTCCCAGGCCTCACACCTGGACCCCCACCCCCAAT
GACTCCCCTCCCAGGCCTCACACCTGGACCCCCACCCTCACCCCTCATCCCCACTGACTCCCCTCCCAGGCCTCACACCTGGACCCCCACCCTCACC
CTCATCCCCACCGACTCCCCTCCCAGGCCTCACACCTGGACCCCCATCCCCTACCCCTCATCCCCGCCGACTCCCCTCCCAGGCCTACACCAGGTCC
CCCACCCCCTCTTGCCTGTCACAGTGCCTCCTGGAACATGGAACCCCTCTAGCAACTCCCTAATTTTCTTCTATTCATAGCAAATTGTCTCAAAAAA
TGTGTCTACACCCACCATCCAAGACCCTGCATCCAAGACGCCCCATCCAAGACCACCCCC

-27 kissing loops
-Promoter flank, CTCF, open chromatin
-Located on LINCO2689

hg38_chr6:170153743-170155616

CCCCGGAGGAGAGCGAGGGGCTCGGGAGGGTCCTCACTCACAGGCACCGGGCGCTTCGACTGCACCCTCATCTCCATCTGCAGGAGGCACAAAGGGG
GACTGACAAGCCCAGTCAGGGTGCATGGGAAGGTCCTTTCCCACTAGACAAGCCAGTAGGGGCCAGGGCCCCAGGGGTACTGGCTGGAGGGGGCGTC
TCCCCCACACCATCCTGGCCCTTGGGCTCACTGCAGACATGGAAGCCAGCAGGCAAAGCTGCACACCCCTCTCCCTCCTCCCAGCCCAGGCCTGTGT
GCTCTCCCTCCTCCCAGCCCAGGCCTGCCTGCTCTCCCTCCTCCCAGCCCAGGCCTGCAGGCCAGGTAGTGATGGATAAGACGGGTCATTGCACCTG
GTGCTGCACTCGCCTCCTGTTCCTTCCAGGAGGGTCTTCTCCAGCCCTGCCGGGAAGACCCTGGTGCACACAGGCCTTCCAGCATGGCCTGCCTAGC
TCCTCGCCCTCCTGGGTCCTCACAGCCCCGGCCTCCTCCCTCCATGGTCGTGCTCTTCTGCTGTCAGCCCCAGGCCTCGCAGTATCGGCCAGAAAAG
CCCATGCCCCTGGGCAAGCCCTGAGTCAGGGCCCCTCCACAGAACCCGGGACCCCTCCACAGAACCCAGGGACCCCTCCACAGAACCCAGGGCCCCT
CCACAGAACCCAGGACCCTTCCACAGAACCCAGGGCCCCTCCACAGAACACAGGGCCCCTCCACAGAACCCAGGGACCCCTCCACAGAACCCAGGGC
CCCTCCACAGAACCCAGGGCCCCTCCACAGAACCCAGGGCCCCTCCACAGAACCCAGGGACCCCTCCACAGAACCCAGGACCCCTCCATAGAACCCA
GGACCCCTCCATAGAACCCAGGGCCCCTCCACAGAACCCAGGGACCCTCCACAGAACCCAGGGACCCCTCCATAGAACCCAGGGCCCCTCCACAGAA
CGCAGGACCCCTCCACAGAACCCAGGGACCCTCCACAGAACCCAGGGCCCCTCCACAGAACCCAGGACCCCTCCACAGAACCCAGGACCCTTCCACA
GAACCCAGGACCCCTCCATAGAACCCAGGACCCTTCCACAGAACCCAGGGCCCCTCCACAGAACCCAGGGACCCCTCCATAGAACCCAGGGCCCCTC
TACAGAACCCAGGGCCCCTCTACAGAACTCAGGACCCCTCTACAGAACCCAGGACCCCTCTACAGAATGCAGGACCCCTCCATAGAACCCAGGACCC
CTCTACAGAACTCAGGACCCCTCTACAGAACCCAGAACCCATCTACAGAACTCAGGACCCCTCTACAGAACCCAGGACCCCTCTACAGAACGCAGGA
CCCCTCCATAGAACCCAGGACCCCTCTACAGAACCCAGGACCCCTCTACAGAACCCAGGACCCCTCTACAGAACCCAGGGCCCCTCTACAGAACTCA
GGACCCCTCTACAGAACCCAGGACCCCTCTACAGAACTCAGGACCCCTCTACAGAACTCAGGACCCCTCTACAGAACCCAGAACCCATCTACAGAAC
CCAGGACCCCTCTACAGAACCCAGGACCCTTCTACAGAACCCAGAACCCATCTACAGAACCCAGGACCCCTCCACAGAACCCAGGACCCCTCTATAG
AACCCAGGACCCCTCTACAGAACCCAGGACCCCTCTACAGAACCGAGGACCCCTCTACAGAACCCAGGACCCCTCTACAGGACCCAGGGCCCCTCTG
TAGAACCCAGGACCCCTCTACAGGACCCAGGGCCCCTCTATAGAACCCAGGGCCCCTCTACAGAACCCAGGACCCCTCTACAGGACCCAGGACCGTT
CTACGGAACCCAGCGCTTGTCCCCTGGCCCC

- 26 kissing loops
- CTCF
- Located on AL596422.1

hg38_chr9_134462726_134467724

Pink compliments Purple

Gray compliments Red

```
>hg38_chr9_134462726_134467724
CCCCGGTGTCATGCCTCAGCTCTTCCCCACACCTCTGCGCTGCTCACCCAGCCGCCCCCTCCCATTAGTG
CCCACTCTGCACCACACGCGCCTCTGCCCGGCTCACCCAGCCGCCCCCCCATTAGTGCCCACTCTGCACC
ACACGCGCCTCTGCCCCGCTCACCCAGCCTCCCCCCCATTAGTGCCCACTCTGCACCACACGCGCCTCTG
CCCCGCTCACCCAGCCTCCCCCCCATTAGTGCCCACTCTGCACCACACGCGCCTCTGCCCGGCTCACCCA
GCCTCCCCCCCATTAGTGCCCACTCTGCACCACACGCACCTCTGCCCCGCTCACCCAGCCGCCCCCTCAT
TAGTGCCCACTCTGCACCACACGCGCCTCTGCCCCGCTCACCCAGCCTCCCCCCCATTAGTGCCCACTCT
GCGCCACACGCGCCTCTGCCCCGCTCACCCAGCCGCCCCCTCATTAGTGCCCACTCTGCACCACACGCGC
CTCTGCCCCGCTCACCCAGCCGCCCCCTCATTAGTGCCCACTCTGCACCACACGCGCCTCTGCCCGGCTC
ACCCAGCCTCCCCCCCATTAGTGCCCACTCTGCACCACACGCGCCTCTGCCCCGCTCACCCAGCCTCCCC
CCCATTAGTGCCCACTCTGCACCACACGCGCCTCTGCCCGGCTCACCCAGCCGCCCCCCCATTAGTGCCC
ACTCTGCACCACACGCGCCTCTGCCCCGCTCACCCAGCCTCCCCCCCATTAGTGCCCACTCTGCACCACA
CGCGCCTCTGCCCCGCTCACCCAGCCTCCCCCCCATTAGTGCCCACTCTGCACCACACGCGCCTCTGCCC
GGCTCACCCAGCCGCCCCCCCATTAGTGCCCACTCTGCACCACACGCGCCTCTGCCCCGCTCACCCAGCC
TCCCCCCCATTAGTGCCCACTCTGCACCACACGCGCCTCTGCCCCGCTCACCCAGCCTCCCCCCCATTAG
TGCCCACTCTGCACCACACGCGCCTCTGCCCGGCTCACCCAGCCGCCCCCCCATTAGTGCCCACTCTGCA
CCACACGCGCCTCTGCCCCGCTCACCCAGCCTCCCCCCCATTAGTGCCCACTCTGCACCACACGCGCCTC
TGCCCGGCTCACCCAGCCGCCCCCCCATTAGTGCCCACTCTGCACCACACGCGCCTCTGCCCCGCTCACC
CAGCCTCCCCCCCATTAGTGCCCACTCTGCACCACACGCGCCTCTGCCCCGCTCACCCAGCCTCCCCCCC
ATTAGTGCCCACTCTGCACCACACGCGCCTCTGCCCGGCTCACCCAGCCGCCCCCCCATTAGTGCCCACT
CTGCACCACACGCACCTCTGCCCCGCTCACCCAGCCGCCCCCTCATTAGTGCCCACTCTGCGCCACACGC
GCCTCTGCCCGGCTCACCCAGCCTCCCCCCCATTAGTGCCCACTCTGCGCCACACGCGCCTCTGCCCCGC
TCACCCAGCCGCCCCCTCATTAGTGCCCACTCTGCACCACACGCGCCTCTGCCCCGCTCACCCAGCCGCC
CCCTCATTAGTGCCCACTCTGCGCCACACGCGCCTCTGCCCGGCTCACCCAGCCTCCCCCCCATTAGTGC
CCACTCTGCACCACACGCGCCTCTGCCCCGCTCACCCAGCCTCCCCCCCATTAGTGCCCACTCTGCACCA
CACGCGCCTCTGCCCGGCTCACCCAGCCGCCCCCCCATTAGTGCCCACTCTGCACCACACGCGCCTCTGC
CCGGCTCACCCAGCCTCCCCCCCATTAGTGCCCACTCTGCACCACACGCGCCTCTGCCCCGCTCACCCAG
CCTCCCCCCCATTAGTGCCCACTCTGCACCACACGCGCCTCTGCCCGGCTCACCCAGCCGCCCCCCCATT
AGTGCCCACTCTGCACCACACGCGCCTCTGCCCCGCTCACCCAGCCTCCCCCCCATTAGTGCCCACTCTG
CACCACACGCGCCTCTGCCCCGCTCACCCAGCCTCCCCCCCATTAGTGCCCACTCTGCGCCACACGCGCC
TCTGCCCCGCTCACCCAGCCGCCCCCTCATTAGTGCCCACTCTGCACCACACGCGCCTCTGCCCCGCTCA
CCCAGCCGCCCCCTCATTAGTGCCCACTCTGCGCCACACGCGCCTCTGCCCCGCTCACCCAGCCGCCCCC
TCATTAGTGCCCACTCTGCGCCACACGCGCCTCTGCCCCGCTCACCCAGCCGCCCCCTCATTAGTGCCCA
CTCTGCGCCACACGCGCCTCTGCCCCGCTCACCCAGCCGCCCCCTCATTAGTGCCCACTCTGCGCCACAC
GCGCCTCTGCCCCGCTCAGCCAGCCGCCCCCTCATTAGTGCCCACTCTGCGCCACACGCGCCTCTGCCCC
GCTCACCCAGCCGCCCCCTCATTAGTGCCCACTCTGCGCCACACGCGCCTCTGCCCCGCTCACCCAGCCG
CCCCCTCATTAGTGCCCACTCTGCACCATACGCGCCTCTGCCCCGCTCACCCAGCCGCCCCTCCATTAGT
GCCCACTCTGTGCCACATGTGCCACTGCCCATGTCCCTTGCTTCAGTTCCAATGGGCCTACTGGCCTCGG
ATCCCTTGTCCACTCCAGGTCTGCACTTGGGAGATGGGGTCACCTGGTGCTGGGCCCCTGAAGTATGCCG
ATGACCTGGGCCCTGCAGAAGTGTCCAGCTTGGGGAGGCGGGAAGCAAAGCAGAGCTCCGGGGTGGCCTT
TGATTTTCTACTTCCCCCAGCCCCATTCCAGGGACCCCTCCACCCTGCTACACCCACCTGCATGCAGGGA
CATGAGCCGAGAGCCCAGAGGGGACCCGTGAGAAGGGAGGGAGGAAAGTGAGAAGACTCCGGTACCAGCA
GCCTGGAGTTACACTGGGACAACCAGACGCTCCAGGGTGCATCAGCAGAGCACGACCCTGTCCCTGAGCA
GCTCCCAGGCGGCTCTGCCATCCTGGAGGCCCCTGGGGCGCTGTCCTATCAGCACCAAGCAGCTGTGCTC
CCAAACTCTGGGGCGTGCAGCCTCAGGGCAAGGTGGCTTTGGGTGGCTGATGGTGCCCCCTCAGGGACTC
TGAGCAGGTCTGAATCTTTCTGAGCCCTTGTTTCTTTAGGTACAAAGCAGAGATGCTTACCTGCCAGATG
GAAGGTCTGGGGGAGGGGAAGGGACTTCAGAACACGCCGCTGGGGCATAAAATTATTTTGTGCAGGAGGC
ATTTGAGTTTCAGAAACCCCTTATCTGAATAAAAGCAGAGCCCCCC
```

hg38_chr19_58538891_58542950

Green compliments
Pink

```
>hg38_chr19_58538891_58542950
CCCACCCACCACGTTTTCAACGCGCTGATAGTCCCTAGGCGGGTTCGCCTCTACCTACAGACCCTGCTTCTCCCCTTAGACATGATTCCTCTCCCCC
AGATCCTGCGTCCCTCAGACAGAGTTCTTCTCTCACACACCCTGCTTCCCTCCTCAGACGGGATTCTCCCCCCACACACCCTGCTTCCCTCCTCAGA
CAGGATTCTCCCCACACACACCCTGCTTCCCTCCTCAGACAGGATTCTTCTCACACACACCCTGCTTCCCTCCTCAGACAGGATTCTCCCCACACAC
ACCCTGCTTCCCTCCTCAGACAGGATTCTCCCCACACAGAACCTGCTTCCCTCCTCAGACAGGATTCTCCCCACACACACCCTGCTTCCCTCCTCAG
ACAGGATTCTCCCCACACAGAACCTGCTTCCCTCCTCAGACAGGATTCTCCCCACACACACCCTGCTTCCCTCCTCAGACATGATTCTTCTCACACA
CACCCTGCTTCCCTCAGACAGGATTCTCCCCACACACACTCTGCTTCCCTCCTCAGACAGGATTCTCCCCACACAGAACCTGCTTCCCTCCTCAGAC
AGGATTCTCCCCACAAACACCCTGCTTCCCTCCTCAGACAGGATTCTCCCCACAGAGACCCTGCTTCCCTCCTCAGACAGGATGCTCCCCACACACA
CCCTGCTTCCCTCCTCAGACAGGATTCTTCTCACACACACCCTGCTTCCCTCCTCAGACAGGATTCTCCCCACACACACCCTGCTTCCCTCCTCAGA
CAGGATTCTCCCCACACACACCCCACTTCCCTCAGACAGGATTCTCCCCACACAGACCCTGCTTCCCTCCTCAGACAGGATTTTCCCCACACAGACC
CTGCTTCCCTCCTCAGACAGGATTCTCCCCACACAGACCCTGCTTCCCTCCTCAGACAGGGCTCCCCTCCCCACACACCCTGCTTCCCTCCTCAGAC
AGAGTTCCCCTCCCCACACAGACCCTCTTTCCCTCAGACAGGATTTTCCCCACACAAACCCTACTTTCCTCCTCAGACAGGATTCTCCCCACACAGA
CTCTGCTTCCCTGAGACAGGATTCTCCCCACACACACCCTGCTTCCCTCCTCAGACAGGATTCTCCCCACACACACCCTGCTTCCCTCCTCAGACAG
GATTCTCACCCCACACACCCTGCTTCCCTCCTCAGACAGGATTCTCCCCACACACACCCTGCTTCCTTCCTCAGACAGGATTCTCCCCCCACACACC
CTGCTTCCCTCCTCAGACAGGATTCTCCCCCCACACACCCTGCTTCCCTCCTCAGACAGGATTCTTCTCACACACACCCTGCTTCCCTCCTCATACA
GGATTCTCCCCACACACACCCTGCTTCCCTCCTCAGACAGGATTCTCCCCACACAGACCCTGCTTCCCTCCTCAGACAGGATTCTCCCCCCACACCC
TGCTTCCCTCCTCAGACAGGATTCTCCCCCCACACACCCTGCTTCCCTCCTCAGACAGGATTCTCCCCACACACACCCTGCTTCCCTCCTCAGACAG
GGCTCCCCCCACACACCCTGCTTCCCTCCTCAGACAGGATTCTCCCCACACAGACCCTACTTCCCTCCTCAGACAGGATGCTCCCCCCACACACCCT
GCTTCCCTCCTCAGACAGGATTCTCCCCCCACACACCCTGCTTCCCACCTCAGACAGGATTCTCCCCCCACACACCCTGCTTCCCTCCTCATACAGG
ATTCTCCCCACACACACCCTGCTTCCCTCCTCAGACAGGATTCTCCCCCCACACATCCTGCTTCCCTCCTCAGACAGGATTTTCCCCACACAAACCC
TACTTTCCTCCTCAGACAGGATTCTCTCCACACAGACCCTGCTTCCCTCAGACAGGATTCTCCCCACACAGACCCTGCTTCCCTCCTCAGGCAGGAT
TCTCCCCACACAGACCCTGCTTCCCTCCTCAGACAGGATTCTCCCCACACAGACCCTGCTTCCCTCCTCAGACAGGATTCTCCCCACACAGACCCTG
CTTCCCTCCTCAGACAGGATTCTCACCCCACACACCCTGCTTCCCTCCTCAGACAGGATTCTCCCCACACACACCCTGCTTCCCTCCTCAGACAGGA
TTCTCCCCCCACACACCCTGCTTCCCTCCTCAGACAGGATGCTCCCCACACACACCCTGCTTCCTTCCTCAGACAGGATTCTCCCCCCACACACCCT
GCTTCCCTCCTCAGACAGGATTCTCCCCCCACACACCCTGCTTCCCTCCTCAGACAGGAAGCTCCCCCCACACACCCTGCTTCCCTCCTCATACAGG
ATTCTCCCCACACACACCCTGCTTCCCTCCTCAGACAGGATTCTCCCCACACAGACCCTGCTTCCCTCCTCAGACAGGATTCTCCCCCCACACCCTG
CTTCCCTCCTCAGACAGGATTCTCCCCCCACACACCCTGCTTCCCTCCTCAGACAGGATTCTCCCCACACACACCCTGCTTCCCTCCTCAGACAGGG
CTCCCCCCACACACCCTGCTTCCCTCCTCAGACAGGATTCTCCCCCCACACACCCTGCTTCCCTCCTCAGACAGGATTCTCTCCACACACACCCTGC
TTCCCTCCTCAGACAGGATTCTCCCCACACACACCCTGCTTCCCTCCTCAGACAGGATTCTCCCCACAGAGACCCTGCTTCCCTCCTCAGACAGGAT
TCTCCTCACACACACCCTGCTTCCCTCCTCAGACAGGATTCTCCCCACACACACCCTGCTTCCCTCCTCAGACAGGATTCTCCCCACACACACCCTG
CTTCCCTCCTCAGACAGGATTCTCCCCACACACACCCCGCTTCCCTCAGACAGGATTCTCCCCACACAGACCCTGCTTCCCTCCTCAGACAGGATTT
TCCCCACACACACCCTGCTTCCCTCCTCAGACAGGATTCTCCCCACACAGACCCTGCTTCCCTCCTCAGACAGGGCTCCCCTCCCCACACAGACCCT
CTTTCCCTCAGACAGGATTTTCCCCACACAAACCCTACTTTCCTCCTCAGACAGGATTCTCCCCACACAGACCCTGCTTCCCTCAGACAGGATTCTC
CCCACACAGACCCTGCTTCCCTCCTCAGGCAGGATTCTCCCCACACAGACCCTGCTTCCCTCCTCAGACAGGATTCTCCCCACACAGACCCTGCTTC
CCTCCTCAGACAGGATTCTCCCCACACAGACCCTGCTTCCCTCCTCAGACAGGATTCTCACCCCACACACCCTGCTTCCCTCCTCAGACAGGATTCT
CCCCACACACACCCTGCTTCCCTCCTCAGACAGGATTCTCCCCACACAGACCCTGCTTCCCTCCTCAGACAGGGATTCCCCTCCCCACACAGACCCT
CTTTCCCTCAGACAGGATTTTCCCCCCACACACCCTGCTTCCCTCCTCAGACAGGATTCTCCCCACATACACCCTGCTTCCCTCCTCAGACAGGATT
CTCCCCACACACACCCTGCTTCCCTCCTCAGACAGGATTCTCCCCACACAGACCCTGCTTCCCTCCTCAGACAGGGCTCCCCTCCCCACACAGACCC
TCTTTCCCTCAGACAGGATTTTCCCCACACACACCCTACTTTCCTCCTCAGACAGGATTCTCCCCACACAGACCCTGCTTCCCTCAGACAGGATTCT
CCCCACTCAGACCCTGCTTCCCTCCTCAGGCAGGATTCTCCCCACACAGAACCTGCTTCCCTCCTCAGACAGGATTCTCCCCACACACACCCTGCTT
CCCTCCTCAGACAGGATTCTCCCCACACACACCCTGCTTCCCTCCTCAGACAGGATTCTCCCCACACAGACGCTGCTTCCCTCCTCAGACAGGATTC
TCCCCACACAGACCCTGCTTCCCTCCTCAGACAGGGTTCCCCTCCGCACACACACCCTCTTTCCCTCAGACAGGATTTTCCCCACACAAACCCTACT
TTCCACCTCAGACAGGATTCTCCCCACATGGACCTGCTTCCTTCCCTCCTTGGGATTCCCCACTGTTGAGGTTCAGTTTCCCC
```

hg38_chr10_133135341_133139883

Pink and Violet compliment
Yellow

Green compliments
Dark Blue

```
>hg38_chr10_133135341_133139883
GGGTGTGAGACAGCCTGGGCTGGTACCATAGGGGAGCTGGTCTCCAGGTCCCTGTTGGGGGGGTCTGGTCTCTGGGTCCCTGTGGGGGG
GGTCTGGTCTCTGGGTCCCTGTGGTGGGGGGGGGTCTCTGGGTCCCTGTGGGGGCGGCTGGTCTCTGGGTCCCTGTGGGGGTGTCTGGT
CTCTGGGTCCCTGTGGTGGGGGGGGTCTCTGGGTCCCTGTTGGGGGGTCTGGTCTCTGGGTCCCTGTAGGGGTTCTGGTCGCTGGGTCC
CTGTAGGCGGGTCTGGTCTCTGGGTCCCTGTGGGGGGGTTCTGGTCTCTGGGTCCCTGTGGTGGGGGGGGGGTCTCTGGGTCCCTGTTG
GGGGGTCTGGTCTCTGGGTCCCTGTGGGGGCGGCTGGTCTCTGGGTCCCTGTGGTGGGGGGGGTCTCTGGGTCCCTGTTGGGGGGTCTG
GTCTCTGGGTCCCTGTGGGGGCGGCTGGTCTCTGGGTCCCTGTGGTGGGTCTGGTCTCTGGGTCCCTGTGGGGGGGTTCTGGTCTCTGG
GTCCCTGTGGGGGCAGCTGGTCTCTGGGTCCCTGTGGGGGTTTCTGGTCTCTGGGTCCCTGTGGGGGGGTCTGGTCTCTGGGTCCCTGT
GGTGGGGGGGGTCTCTGGGTCCCTGTGGGGGGGTCTGGTCTCTGGGTACCTGTGGGGGCAGCTGGTCTCTGGGTCCCTGTGGGGGGGTT
CTGGTCTCTGGGTCCCTGTGGGGGCGGCTTGTCTCTGGGTCTCTGTGGGGGGGTCTGGTCTCTGGGTCCCTGTGGAGGCGGCTGGTCTC
TGGGTCCCTGTAGGGGTTCTGGTCTCTGGGTCCCTGTGGGCGGGTCTGGTCTCTGGGTCTGTTGGGGGGGTCTGGTCTCTGGGTCCCTG
TGGGGGGGGTCTGGTCTCTGGGTCCCTGTGGTGGGGGGGGGTCTCTGGGTCCCTGTGGGGGCGGCTGGTCTCTGGGTCCCTGTGGGGGT
GTCTGGTCTCTGGGTCCCTGTGGTGGGGGGGGTCTCTGGGTCCCTGTTGGGGGGTCTGGTCTCTGGGTCCCTGTAGGGGTTCTGGTCGC
TGGGTCCCTGTAGGCGGGTCTGGTCTCTGGGTCCCTGTGGGGGGGTTCTGGTCTCTGGGTCCCTGTGGGGGTGTCTGGTCTCTGGGTCC
CTGTGGTGGGGGGGGTCTCTGGGTCCCTGTTGGGGGGTCTGGTCTCTGGGTCCCTGTAGGGGTTCTGGTCTCTGGGTCCCTGTGGGCGG
GTCTGGTCTCTGGGTCCCTTTGGGGGGGTCCTGGTCTCTGGGTCCCTGTGGGGGCGGCTGGTCTCTGGGTCCCTGTGGGGGGTTCTGGT
CTCTGGGTCCCTGTGGGGGCGGCTGATCTCTGGGTCCCTGTGGGGGGGTCTGGTCTCTGGGTCCCTGTTGGGGGGTCTGGTCTCTGGGT
CTCTGTGGGGGTTCTGGTCTCTGGGTCCCTGGGGGGGCGGCTGGTCTCTGGGTCCCTGTGGGGGGTTCTGGTCTCTGGGTCCCTGTGGG
GGCGGCTGGTCTCTGGGTCTCTGTGGGGGGGTCTGGTCTCTGGGTCCCTGTGGGCGGGTCTGGTCTCTGGGTCCCTGTGGAGGCGGCTG
GTCTCTGGGTCCCTGTGGGCGGGTCTGGTCTCTGGGTCCCTGTTGGGGGGTCTGGTCTCTGGGTCCCTGTGGGGGGGTTCTGGTCTCTG
GGTCCCTGTGGGGGCAACTGGTCTCTGGGTCCCTGTGAGAGGTTCTGGTCTCTGGGTCCCTGTGGGGGGGTCTGGTCTCTGGGTCCCTG
TGGGGGGATCTGGTCTCTGGGTCCCTGTGGTGGGGGGGGTCTCTGGGTCCCTGTGGGGGGGTCTGGTCTCTGGGTACCTGTGGGGGCAG
CTGGTCTCTGGGTCCCTGTGGGGGGGTTCTGGTCTCTGGGTCCCTGTGGGGGCGGCTTGTCTCTGGGTCTCTGTGGGGGGGTCTGGTCT
CTGGGTCCCTGTGGAGGCGGCTGGTCTCTGGGTCCCTGTAGGGGTTCTGGTCTCTGGGTCCCTGTGGGCGGGTCTGGTCTCTGGGTCCC
TGTTGGGGGGTCTGGTCTCTGGGTACCTGTGGGGGTGTCTGGTCTCTGGGTCCCTGTGGTGGGGGGGTCTCTGGGTCCCTGTTGGGGGG
TCTGGTCTCTGGGTCCCTGTAGGGGTTCTGGTCTCTGGGTCCCTGTGGGCGGGTCTGGTCTCTGGGTCCCTGTGGGGGGGTCCTGGTCT
CTGGGTCCCTGTGGGGGCGGCTGGTCTCTGGGTCCCTGTGAGGGGTTCTGGTCTCTGGGTCCCTGTGGGGGCGGCTGATCTCTGGGTCC
CTGTGGGGGGGTCTGGTCTCTGGGTCCCTGTAGGGGCGGCTGGTCTCTGGGTCCCTGTTGGGGGGTCTTGTCTCTGGGTCCCTGTAGGG
GTTCTGGTCCCTGGGTCCCTGTAGGCGGGTCTGGTCTCTGGGTCCCTGTTGGGGGTTCTGGTCTCTGGGTCCCTGTGGGGGCGGCTGGT
CTCTGGGTCCCTGTGGGGGTTCTGGTCCCTGGGTCCCTGTGGGGGCGGCTGGTCTCTGGGTCCCTGTGGGGGTTCTGGTCCCTGGGTCC
CTGTGGGGGCGGCTGGTCTCTGGGTCCCTGTGGGGGCGGCTGGTCTCTGGGTCCCTGTGGGGGGTTCTGGTCCCTGGGTCCCTGTGGGC
GGGTCTGGTCTCTGCATCCCTGTGTGGGGGTGGCTGGTCTCTGGGTCCCTGTGGGCGGGTCTGGTCTCTGGGTCCCTGTGTGGGGGTGG
CTGGTCTCTGGGTCCCTGTGGGGGCGGCTGGTCTCTGGGTCCCTGTGGGGGCGGCTGGTCTCTGGGTCCCTGTGGGGGCGGCTGGTCTC
TGGGTCCCTGTGGGGGGGTCTGGTCTCTGGGTCCCTGTGGGGGCGGCTGGTCTCTGGGTCCCTGTGGGGGGTCTGGTCTCTGGGTCC
GTGTGGGGGTGGCTGGTCTCTGGGTCCCTGTGGGGGCGGCTGGTCTCTGGGTCCCTGTGGGCGGGTCTGGTCTCTGGGTCCCTGTGGGG
GGGGTCTGGTCTCTGGGTCCCTGTGGGGGCGGCTGGTCTCTGGGTCCCTGTGGGGGGGTCTGGTCTCTGGGTCCCTGTGGGGGGCCTG
GTCTCTGGGTCCCTGTGGGCGGGTCTGGTCTCTGGGTCCCTGTGGGCGGGTCTGGTCTCTGGGTCCCTGTTGGGGGGGTCTGGTCTCTG
GGTCCCTGTGGGGGTGTCTGGTCTCTGGGTCCCTGTGGTGGGGGGGGGGTCTCTGGGTCCCTGTTGGGGGGTCTGGTCTCTGGGTCCCT
GTGGGGGCGGCTGGTCTCTGGGTCCCTGTGGTGGGGGGGGTCTCTGGGTCCCTGTTGGGGGGTCTGGTCTCTGGGTCCCTGTGGGGGCG
GCTGGTCTCTGGGTCCCTGTGGTGGGTCTGGTCTCTGGGTCCCTGTGGGGGGGTTCTGGTCTCTGGGTCCCTGTGGGGGCAGCTGGTCT
CTGGGTCCCTGTGGGGGTTTCTGGTCTCTGGGTCCCTGTGGGGGCAGCTGGTCTCTGGGTCCCTGTGGGGGGTCCTGGTCTCTGGGTC
CCTGTGGGGGCGGCTGGTCTCTGGGTCCCTGTGGGGGGTTCTGGTCTCTGGGTCCCTGTGGGGGGTTCTGGTCTCTGGGTCCCTGTGAG
GTGGCTGGTCTCTGGGTCCCTGTGGGGGGGTCTGGTCCCTGGGTCCCTGTGGGGGGGTCTGGTCTCTGGGTCCCTGTGGGGGCGGCTCA
TCTCTGGGTCCCTGTTGGGGGCTCTGGTCTCTGGGTACCTGTGGCGGGGGGGGTCTCTGGGTCCCTGTTGGGGGGTCTGGTCTCTGGGT
CCCTGTTGGGGCGGCTGGTCTCTGGGTCCCTGTGGGGGTGTCTGGTCTCTGGGTCCCTGTGGTTGGGGGGGTCTCTGGGTCCCTGTTGG
GGGGTCTGGTCTCTGGGTCCCTGTGGGGGGGTCTGGTCTCTGGGTCCCTGTGTGCGGGTGGCTGGTCTCTGGGTCCCTGTGGGGGGGT
CTGGTCTCTGGGCCCCTGTGTGGGGGTGGCTGGTCTCTGGGTCCCTGTTGGGGGGTCTGGTCTCTGGGTCCCTGTGGGGGGTCTGGTC
TCTGGGTCCCTGTGGGGGGGCCTGGTCTCTGGGTCCCTGTGGGCGGGTCTGGTCTCTGGGTCCCTGTGGGGGTGGGTGGTCTCTGGGTC
CCTGTGGGGGGTCTGGTCTCTGGGTCCCTGTGGGGGAGTCTGGTCTCTGGGTCCCTGTGGGGGCGGCTGGTCTCCAGGGCTCTGTCGG
GGGCAGCAGGATAGTCAGTCAGTGGTGGGATTCTCACCCTGACTTGCTCTTTGGACCAAGGTGGGCTCATTGGTCAACTGGGGAGGAAG
GAAGAGGCGGCAGCAAACCTCAGCAGGCAGCTGGGAAGCCACAGCGGGG
```

hg38_chr14_105185853_105187262

Pink compliments Violet

Green compliments Teal

Gray compliments Yellow

```
>hg38_chr14_105185853_105187262
GGGGGTGCGGGGGGTGGGGAGGACGTGGGGGAGTCAGGGGCGGGAGGGGTGCAGGAAAGCCAGGGGCGGGGATTCCG
AGGTAAGTGCAGAGGGAGTGTCCCGAGAGAGGCTGAGTTGAGGGGAAGCTGGAGGAGGATCAGGATGGGGGGTCAGG
AGGTCGGGGGTGTGGGGCACAGAAGTCCACTGGGTGTGGGCAGGAGTTGAGTGCAGAGTGCTCATAAGTGAGCAGAG
TGGTGGGCTCCAGGACTGGCAGTGGGGTGCTGGGGGGATACAGCAGAGACCACACCCGGAGGAGCAGCAGGGGGCTG
GGCCGTGTGTGTGTGTGTGTGTGTGTGTGTGTATCTGTGCATCTCTGTGTGTCTGTGTGTGTAATGGAGAGAAGC
AGTATGTGTGTGTGTGGAGGTGTGTGTGGGGGGCTGTGTGTGGGGGGTGTACATGAGTGTGTGGGGGGTATGTGTGT
GAGTGTGGGGGAGTATGTGGGGGGGTGTGTGGGAGTGAATGTGGGGGTATGTGTGTGGGGGGGTGTGTGTGTATGGG
GTGTGTGTGTGGGGTATGTGAGTGGGTGTGGGGGTATGTGTGTGGGGGGGTATGTGTGTGAGTGTGGGGGAGTATGT
GGGGGGGTGTGTGGGAGTGAATGTGGGGGTATGTGTGTGGGGGGGTGTGTGTGTATGGGGTGTGTGTGTGGGGTATG
TGAGTGGGTGTGGGGGTATGTGTGTGGGGGGGTATGTGTGTGAGTGTGGGGGAGTATGTGGGGGGGTGTGTGGGAGT
GAATGTGGGGGTATGTGTGTGGGGGGGTGTGTGTGTATGGGGTGTGTGTGTGGGGTATGTGAGTGGGTGTGGGGGTA
TGTGTGGGTGTGTATAGGGGGTGTGTGGGAGGTGTGTGTGTGGGCTGTGTGTGTGGGGGTATCTTTGTGGGGGGTAT
GGGGGGTGTGGGGGGTGTGTGTGTGTGAGTGTGGGGGGTATGTGTGTGGGGCGTATGTGTATGGGGGTATGTGTGTG
TGAGTGTGGGGGGTATGTGTGTGGGGTGTATGTGTATGGGTGTGTGTGTGAGAGTGTGGGGGTATGTGTGTAGGGGG
TATGTGTGTGGGGGTGAGTGTGTGGGTGTGTGTGGGTGTGTATAGGGGGTGTGTGGGAGGTGTGTGTGTGTGTGTGG
GTATGTGTGTGGGGGTATGTGTGGGGGGTATGTTTGTGGGGGGGTGGGGGGTATGTGTGTGGGGGGATATGTATATG
TGGGGGTGTGTATAGGGGGTGTGTGTGTGGGGGGTATGTGTGCGTGTGTGTGAGTGTGGGGGGGTATGTGTGGGGGGT
GTGTGTGTGAGTGTGTGTGCAGCCAAGCTTCTGCACTGAGTAGGAATAGGGCGGCTTCACTAAGCAAAGGGG
```

Supplementary Information 5. Nondenaturing G4 gel electrophoresis and sequential staining. (**A**) G4 formation of G4-capable oligonucleotides with third loop lengths of 3, 10, and 20 nts. (**B**) G4 formation of oligonucleotides with third loop sequences based on known HCV kissing loops. (**C**) G4 formation of oligonucleotides consisting of a tandem of minimal G4 capable sequences confirmed to form G4 in ((A), Ctg20loop) and ((B), HCV20loops). (**D**) G4 formation of oligonucleotides consisting of a tandem of minimal G4 capable sequences taken from the LG4 detailed in Figure 7A.

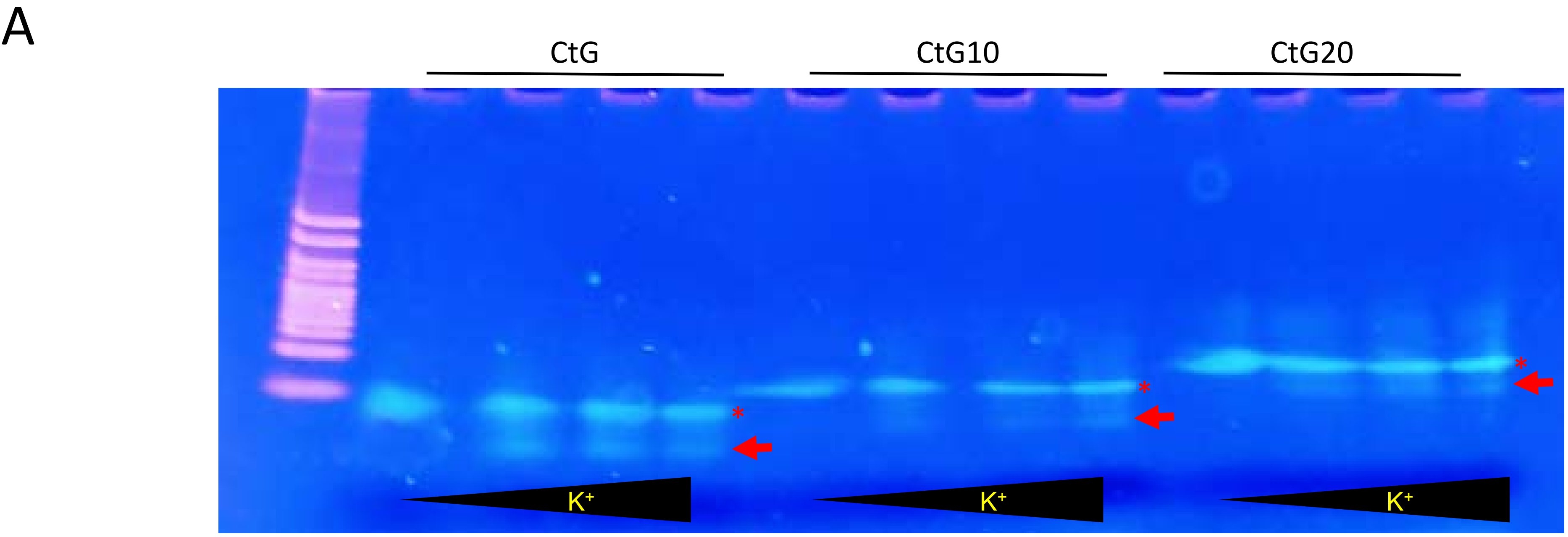

*	ssDNA Oligonucleotide
(red arrow)	Intramolecular G4 bands
K^+	Relative Potassium Concentration

CtG

GGGGAAAGGGGAAAGGGGAAAGGGG

Ctg10 loop

GGGGAAAGGGGAAAGGGGAAAAAAAAAAGGGG

Ctg20 loop

GGGGAAAGGGGAAAGGGGAAAAAAAAAAAAAAAAAAAAGGGG

B

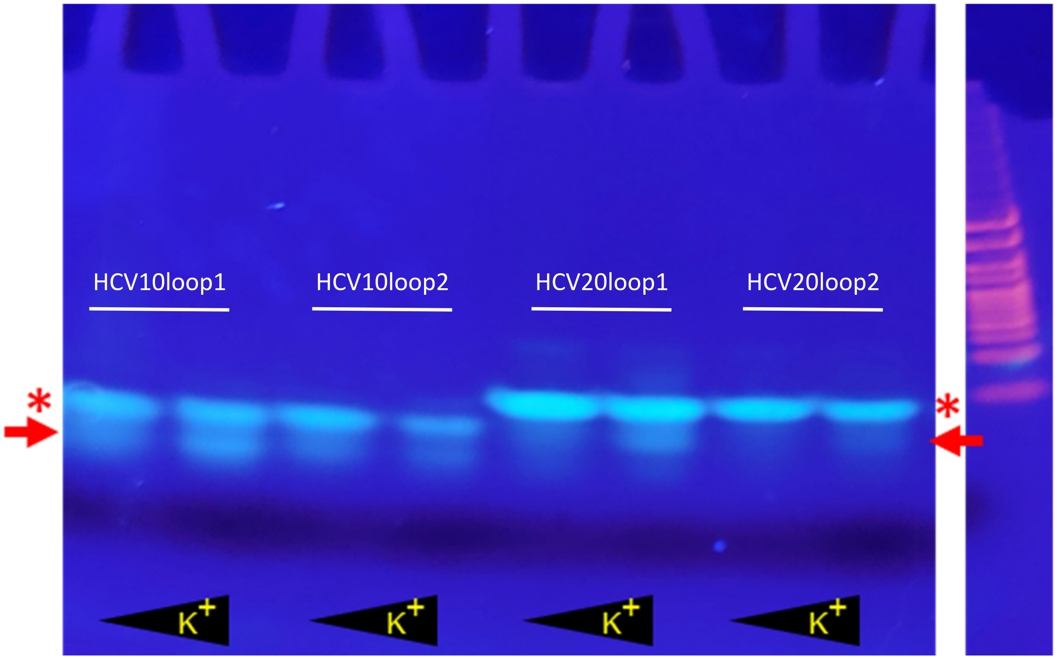

*	ssDNA Oligonucleotide
→	Intramolecular G4 bands
K+	Relative Potassium Concentration
BLUE FONT (right)	Loop sequences based on known HCV kissing loop (Friebe et al.)

HCV10loop1
GGGGAAAGGGGAAAGGGGAATCGCAGCAGGGG
HCV10loop2
GGGGAAAGGGGAAAGGGGAGCTGCGATAGGGG
HCV20loop1
GGGGAAAGGGGAAAGGGGAAAAAAATCGCAGCAAAAAAGGGG
HCV20loop2
GGGGAAAGGGGAAAGGGGAAAAAAGCTGCGATAAAAAAGGGG

Friebe P, Boudet J, Simorre JP, Bartenschlager R. Kissing-loop interaction in the 3' end of the hepatitis C virus genome essential for RNA replication. *J Virol.* 2005 Jan;79(1):380-92. PMID: 15596831

C

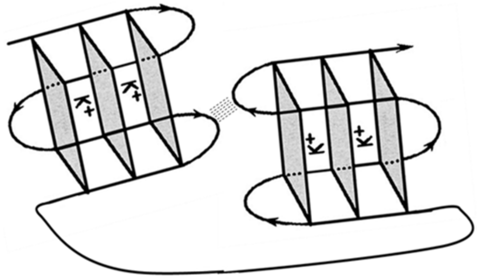

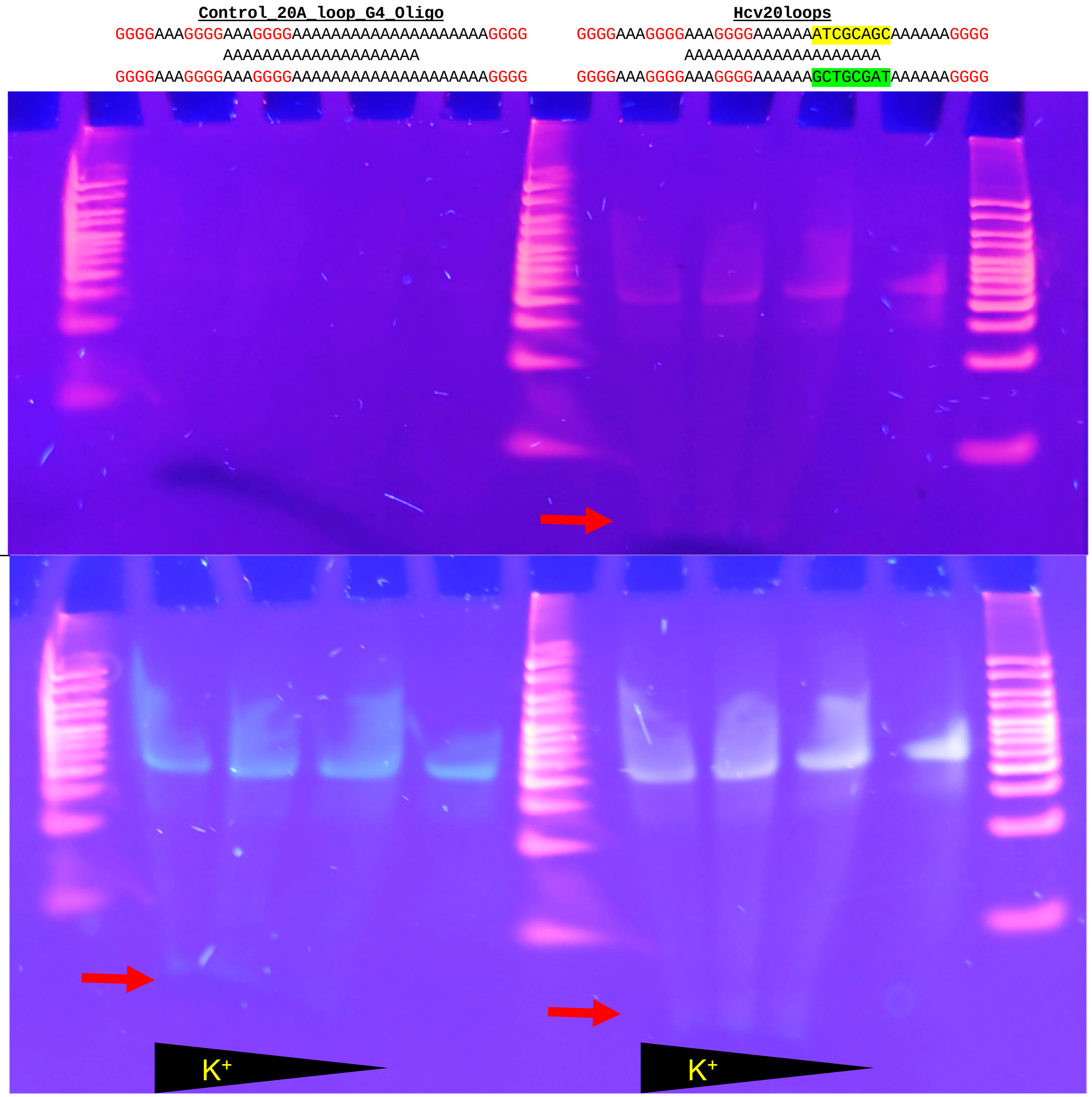

Upper Gel: EtBr stain (orange) only. The affinity for EtBr binding of dsDNA is ~25x its affinity for ssDNA.

Lower Gel: EtBr (orange) and Thioflavin (blue) stains. Thioflavin specifically binds ssDNA and G4 DNA.

Intramolecular G4 bands.

Relative Potassium Concentration.

D

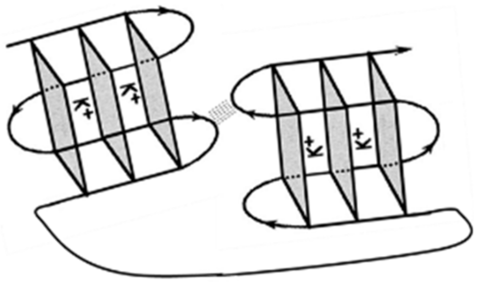

Upper Gel: EtBr stain (orange) only. The affinity for EtBr binding of dsDNA is ~25x its affinity for ssDNA.

Lower Gel: EtBr (orange) and Thioflavin (blue) stains. Thioflavin specifically binds ssDNA and G4 DNA.

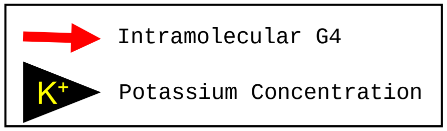

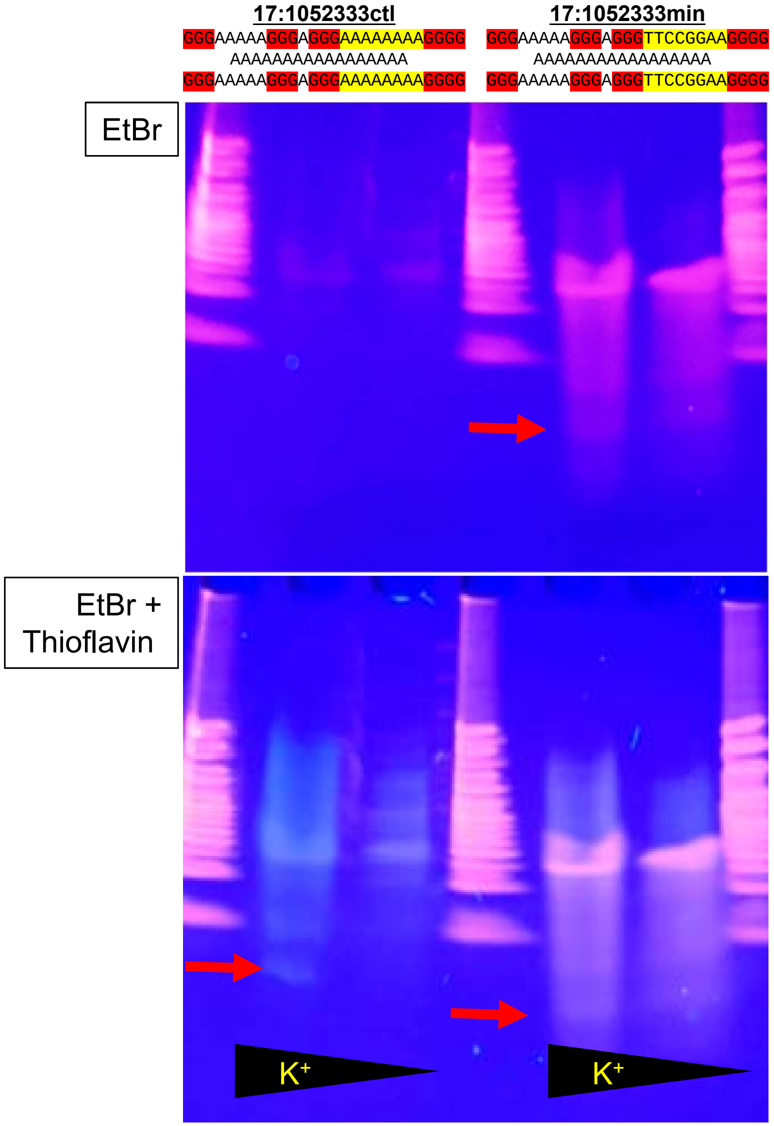

Supplement: gkaa357_Supplemental_Files [file gkaa357_supplemental_files.zip › Supplementary Files.pdf]
